# Ring Strain‐Promoted Activation of Pyridines by a Saturated BSi_2_ Cycle

**DOI:** 10.1002/anie.202517462

**Published:** 2025-11-23

**Authors:** Nasrina Parvin, Ankur, Philipp Willmes, Bernd Morgenstern, Cem B. Yildiz, David Scheschkewitz

**Affiliations:** ^1^ Chair in General and Inorganic Chemistry Saarland University 66123 Saarbrücken Germany; ^2^ Service Center X‐ray diffraction Saarland University 66123 Saarbrücken Germany; ^3^ Department of Basic Sciences Faculty of Engineering Architecture and Design Bartin University Bartin 74100 Turkey

**Keywords:** Bond activation, Boron, Ring strain, Silicon, Small rings

## Abstract

Ring strain is a well‐established strategy to increase reactivity. Employing an elusive saturated BSi_2_ ring motif, we here exploit the size mismatch between boron and silicon to this end. The reaction of disilenide Tip_2_Si = SiTipLi with BH_3_·SMe_2_ selectively affords the lithium salt of anionic boratadisilirane *c*‐SiTip_2_SiHTipBH_2_^−^ (Tip = 2,4,6‐triisopropylphenyl), which according to DFT calculations on the parent system BSi_2_H_6_^−^ is much more strained than isoelectronic analogues such as cyclopropane (C_3_H_6_), boratirane (BC_2_H_6_^−^), and cyclotrisilane (Si_3_H_6_). Indeed, it spontaneously and selectively activates a range of pyridine derivatives: pyridine itself undergoes *ortho*‐CH activation under dearomatization of a second equivalent; 2 equivalents of *para*‐dimethylaminopyridine (DMAP) are C─C‐coupled in *ortho*‐ and *meta*‐position; and pentafluoropyridine (PFP) is CF‐activated in *para*‐position.

## Graphical abstract

We report a highly strained boratadisilirane with the elusive anionic BSi_2_ ring motif. Cleavage of the Si─Si bond during reactions with pyridine derivatives activates them in three different ways: 1) for pyridine itself selective *ortho*‐CH activation and concomitant hydrogen transfer to a second pyridine molecule occurs, 2) with dimethylaminopyridine, unprecedented C─C bond formation happens between the two DMAP equivalents, and 3) in pentafluoropyridine the *para*‐CF bond is activated under formation of regioisomers.

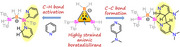

## Introduction

Due to their considerable ring strain and the ensuing high reactivity, cyclopropanes are invaluable synthons in all areas of organic chemistry.^[^ ^1^, ^2^, ^3^ ^]^ In addition, they find diverse applications beyond synthesis, for example, in biologically active natural products,^[^ ^4^, ^5^, ^6^ ^]^ pharmaceuticals,^[^ ^7^, ^8^, ^9^ ^]^ and as polymerization precursors.^[^ ^10^, ^11^ ^]^ Heterocycles allow for the differentiation of the endocyclic bonds (including donor–acceptor systems) and thus further increase the scope of three‐membered rings.^[^ ^12^, ^13^, ^14^, ^15^, ^16^ ^]^ Heavier homologues add to the toolbox due to their inherently weaker bonding,^[^ ^17^, ^18^, ^19^, ^20^, ^21^, ^22^ ^]^ with small silacycles having enjoyed particular attention:^[^ ^23^, ^24^, ^25^ ^]^ cyclotrisilanes and related unsaturated examples exhibit unique reactivities such as, for instance, the photolytic cleavage into transient disilenes and silylene,^[^ ^26^, ^27^, ^28^ ^]^ expansion to larger rings and clusters,^[^ ^29^, ^30^, ^31^ ^]^ or anionic ring‐opening polymerization to polysilanes.^[^ ^32^, ^33^, ^34^ ^]^

According to very recent theoretical calculations by Espinosa Ferao,^[^ ^35^ ^]^ the incorporation of boron into small ring systems exerts a considerable influence on ring strain. While the saturated borirane exhibits larger ring strain compared to cyclopropane, the unsaturated borirene is less strained than cyclopropene due to the 2π‐aromatic stabilization present in the former.^[^ ^36^ ^]^ We recently confirmed the facile carbonylative ring expansion of a diborirane.^[^ ^37^, ^38^ ^]^

**Scheme 1:**
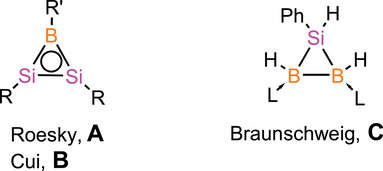
Reported examples of three‐membered rings with boron and silicon as heteroatoms (**A**: R = PhC(N*t*Bu)_2_, R’ = Tip; **B**: R = boryl, R’ = NPh_2_; **C**: L = 1,3‐bis(2,6‐diethylphenyl)‐4,5‐dihydroimidazol‐2‐ylidene).

As noted above, the inclusion of heavier atoms can be expected to further increase strain and thus, weaken the endocyclic bonds.^[^ ^35^, ^39^, ^40^ ^]^ There are, however, only three examples of B/Si three‐membered rings documented in the literature: the unsaturated disilaborirenes **A** and **B** from the Roesky and Cui groups, respectively,^[^ ^41^, ^42^ ^]^ and the NHC‐adduct of saturated siladiborirane **C**, reported by Braunschweig and coworkers (Scheme 1).^[^ ^43^ ^]^ Such highly strained compounds hold significant promise in terms of reactivity, although so far remain largely unexplored apart from the ring expansion of **A** with trimethylsilyl azide as nitrene source.^[^ ^41^ ^]^

In our recent study on AlSi_3_ ring formation from the reaction of lithium disilenide **1** with H_2_AlCl,^[^ ^26^, ^44^, ^45^, ^46^, ^47^, ^48^, ^49^, ^50^, ^51^ ^]^ we proposed the intermediacy of an AlSi_2_ ring, considered to be among the most strained ring systems according to computational studies by Pannels and Espinosa–Ferao.^[^ ^40^ ^]^ Extending our approach to the Si/B manifold, we now report the synthesis and characterization of an unprecedented anionic saturated BSi_2_ ring. DFT computations confirm the competitively high strain of this three‐membered ring and we show that it readily reacts with a range of pyridines under ring opening to give rise to distinct activation products in a highly selective and case‐sensitive manner.

## Results and Discussion

Disilenides are well‐established precursors for small ring compounds such as ESi_2_, E_2_Si_2_ or ESi_3_ (E = Si, Ge, P, and Al), through their reaction with suitable electrophiles.^[^ ^26^, ^44^, ^45^, ^46^, ^47^, ^48^, ^49^, ^50^, ^51^ ^]^ Although reactions of a disilenide with monohaloboranes have been reported to yield boryl disilenes,^[^ ^52^, ^53^ ^]^ the residual substituents of the boryl group apparently prevented the cyclization to three‐membered rings. Recently, Cui et al. disclosed the synthesis of disilaborirene **B** from the reaction of a dilithiodisilene with a dihaloborane.^[^ ^42^ ^]^

**Scheme 2:**
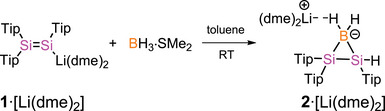
Synthesis of disilaboratirane **2**·[Li(dme)_2_] from **1**·[Li(dme)_2_] (Tip = 2,4,6‐*i*Pr_3_C_6_H_2_, dme = 1,2‐dimethoxyethane).

Reaction of lithium disilenide **1**·[Li(dme)_2_]^[^ ^54^ ^]^ with BH_3_·SMe_2_ in toluene at room temperature leads to selective and complete conversion to a new product, which is isolated as a yellow solid in 71% yield (Scheme 2). In the solid state, the product is stable at room temperature but in hexane solution slowly decomposes to a mixture of unidentified products. A diagnostic ^11^B NMR triplet at *δ* =−37.9 ppm (*^1^J_B,H_* = 99.1 Hz) suggests a tetracoordinate boron center with only two directly attached hydrogen atoms. The shift of the third hydrogen atom to an adjacent silicon center is confirmed by the ^1^H NMR signal at 3.95 ppm with ^29^Si satellites (*^1^J_Si,H _* = 178.9 Hz). The two ^29^Si NMR signals at − 82.1 and − 102.8 ppm are both broadened by coupling to the quadrupolar ^11^B nucleus, but only the latter exhibits a cross‐peak in the 2D ^1^H/^29^Si correlation NMR spectrum. The considerable upfield shift of the hetero nuclei is in line with the formation of a three‐membered ring.^[^ ^26^, ^45^, ^46^, ^47^, ^48^, ^49^, ^50^, ^51^ ^]^ In concert, the NMR spectroscopic data strongly support the formation of the boratadisilirane **2**·[Li(dme)_2_].

**Figure 1:**
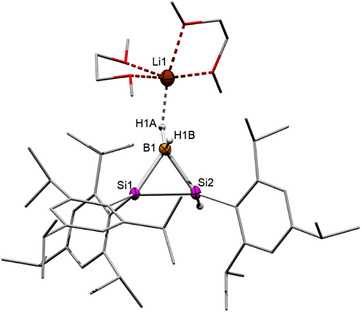
Molecular structure of **2**·[Li(dme)_2_] in the solid state. Hydrogen atoms are omitted for clarity. Thermal ellipsoids at 50% probability. Selected bond lengths (Å) and bond angles (°) of **2**·[Li(dme)_2_]: B1‐Si1 2.027(2), B1‐Si2 2.012(2), Si1‐Si2 2.325(5), H1A···Li1 2.035(2), H1B···Li 2.525(2), B1···Li1 2.667(3); Si1‐B1‐Si2 70.27(6), B1‐Si1‐Si2 54.56(5), B1‐Si2‐Si1 55.17(5).

Single crystals were obtained from a saturated hexane solution at − 30 °C.^[^ ^55^ ^]^ A x‐ray diffraction study confirmed the constitution of **2**·[Li(dme)_2_] in the solid state as three‐membered ring system with an anionic BH_2_ borate bridging the two silicon centers (Figure 1). The Li^+^ counter cation is coordinated by one B─H bond of the borate center as well as by two molecules of 1,2‐dimethoxyethane. The distance between Si1 and Si2 of 2.325(5) Å is in the range of Si─Si single bonds.^[^ ^56^, ^57^, ^58^ ^]^ The B─Si bonds of 2.027(2) and 2.012(2) Å in **2**·[Li(dme)_2_] are significantly elongated compared to the unsaturated boron doped silacycles **A**, **B** (1.911(7) to 1.952(3) Å) and similar to the saturated silicon ring in **C** (2.007(3) to 2.008(3) Å).^[^ ^41^, ^42^, ^43^, ^59^, ^60^, ^61^, ^62^, ^63^, ^64^, ^65^ ^]^ The distance between B1 and Li1 of 2.667(3) Å matches well those of acyclic lithium hydridoborates.^[^ ^66^, ^67^, ^68^, ^69^ ^]^

**Figure 2:**
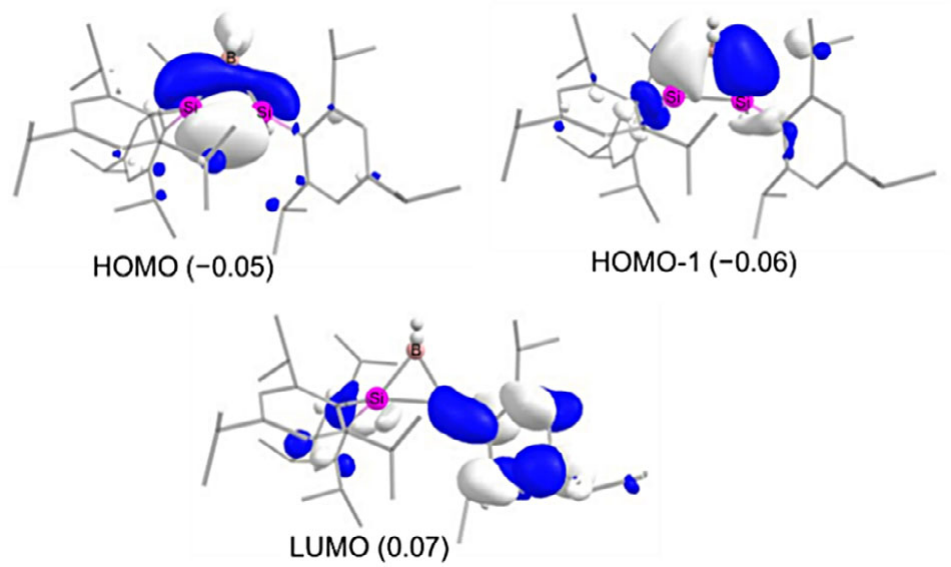
Selected frontier orbitals of **2** (energy in eV, contour value = 0.04).

**Figure 3:**
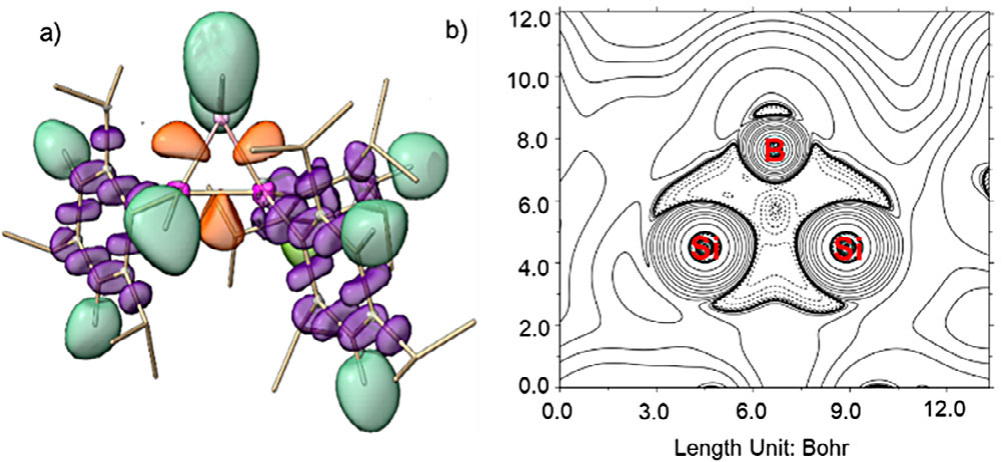
a) ELF isosurface of **2** (Orange: B─Si and Si─Si bonds; Purple: C─C bonds; Green: H atoms); b) Laplacian of electron density contour plot in the plane of the BSi_2_ subunit of **2**. Hydrogen atoms except for Si–H and B–H are omitted for clarity.

In order to understand the electronic structure of anionic **2** we optimized its structure at the B3LYP‐D3BJ/def2SVP level of theory resulting in good agreement in bond distances and bond angles of the BSi_2_ ring with experimental results. The highest occupied molecular orbital (HOMO) of **2** is primarily composed of a Si─Si σ‐bond, with a minor contribution from hydrogen orbitals of B–H bond due to simple symmetry considerations. According to NBO calculations, the Si─Si bond exhibits a strong p‐character of 81.2% and 83.5%. In comparison, the HOMO− 1 corresponds to the σ‐bonds of two B─Si bonds, also illustrating notable p‐character: 69.9% and 66.6% for the silicon atoms, and 78.4% and 78.9% for the boron atoms of Si─B bonds. The predominant p‐character in both HOMO and HOMO−1 exemplifies the typical “banana bonds” found in three‐membered rings. The LUMO mainly corresponds to the π*‐antibonding orbital of the Tip group attached to the silicon center (Figure 2). The Si─B bonds are decidedly nonpolar as manifest in near‐equal electron distribution across Si (49.9% and 50.0%) and B (50.5% and 49.5%). The Wiberg bond indices (WBI of Si─Si: 0.91; B─Si: 0.95 and 1.00) are expectedly in line with Si─Si and B─Si single bonds. Bader's quantum theory of atoms in molecules (QTAIM) confirms that the Si─Si bond in **2** is weaker than the B─Si bonds (Table S7). Plots of the electron localization function (ELF) and Laplacian of electron density expectedly show that the electron densities are depleted along the internuclear axes. The increased off‐axis electron density confirms bent σ bonding as familiar in three‐membered ring systems (Figure 3).

**Scheme 3:**
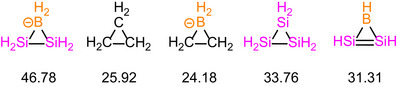
Ring strain energy (RSE) of **2H** (BSi_2_H_6_^−^) in comparison to isoelectronic species C_3_H_6_, BC_2_H_6_^−^, Si_3_H_6_, and unsaturated BSi_2_H_3_ [kcal mol^−1^].

Drawing inspiration from the DFT studies on the ring strain of three‐membered rings by Espinosa–Ferao et al.,^[^ ^35^, ^39^, ^40^ ^]^ we investigated **2H**, the hydrogen‐substituted parent species of **2**, and compared it to its isoelectronic analogues C_3_H_6_, Si_3_H_6_, and BC_2_H_6_^−^ as well as the unsaturated version BSi_2_H_3_. The calculations were performed at the B3LYP/def2SVP level of theory using a homodesmotic reaction (Table S8). The ring strain remains approximately the same upon replacement of one of the CH_2_ groups in cyclopropane by BH_2_^−^ (Scheme 3, C_3_H_6_ 25.92 vs. BC_2_H_6_^−^ 24.18 kcal mol^−1^) due to the similar sizes of carbon and boron. In contrast, a significant increase in ring strain is observed when one of the SiH_2_ in trisilacyclopropane is replaced by a much smaller BH_2_^−^ unit (Si_3_H_6_ 33.76 vs. **2H** 46.78 kcal mol^−1^), highlighting the importance of the size differences between boron and silicon. Remarkably, the unsaturated BSi_2_H_3_ ring as hydrogen‐substituted parent of **A** and **B** ^[^ ^41^, ^42^ ^]^ (31.31 kcal mol^−1^) exhibits greater stability compared to **2H**, which can be explained by the stabilizing effect of the 2π aromatic system—as previously noted for the corresponding BC_2_ system.^[^ ^36^, ^39^ ^]^

**Scheme 4:**
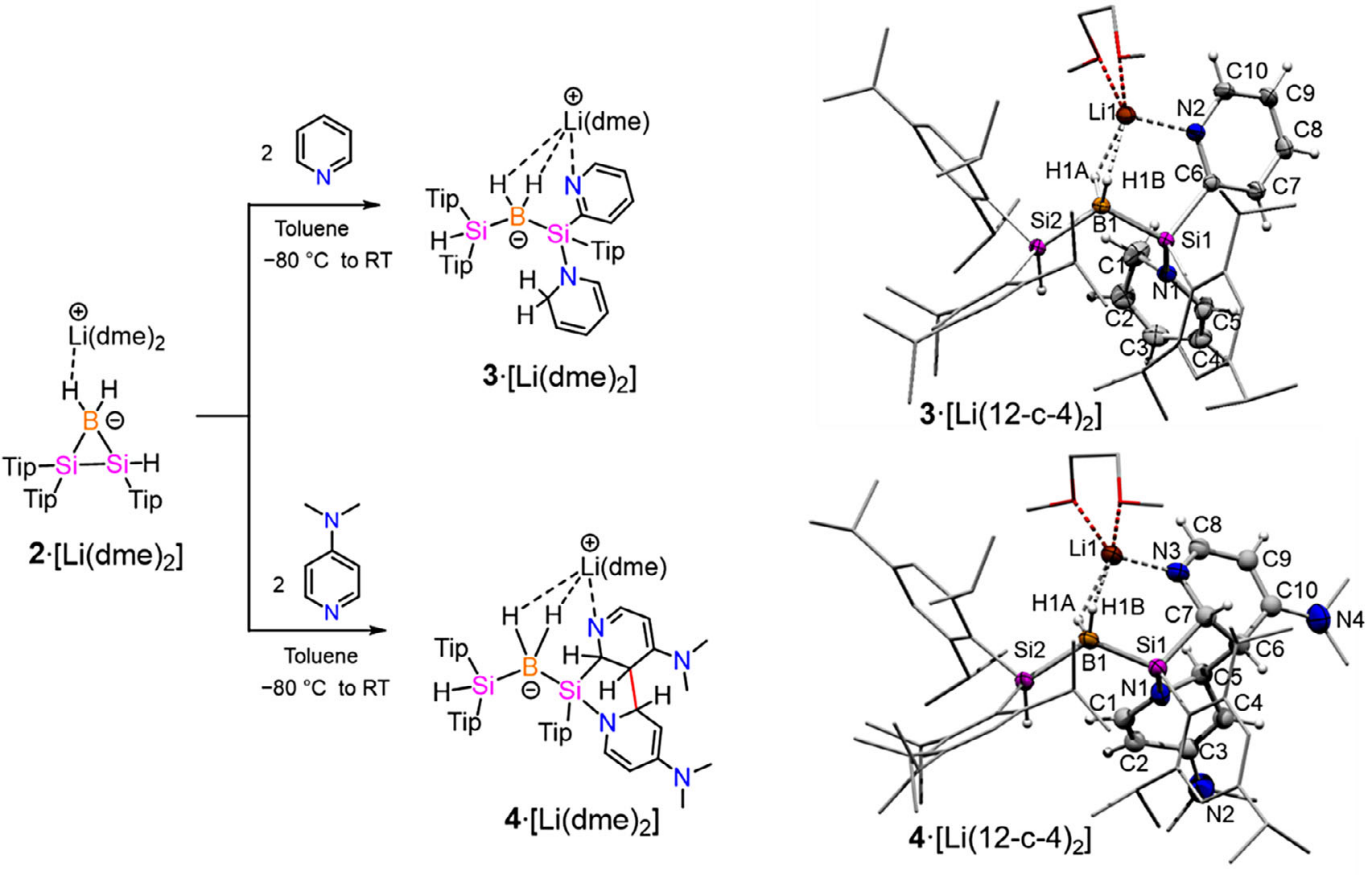
Reactivity of **2**·[Li(dme)_2_] with pyridine and DMAP (left) and molecular structure of **3**·[Li(dme)_2_] and **4**·[Li(dme)_2_] in the solid state (right). Hydrogen atoms and co‐crystallized solvent molecules (hexane for **4**·[Li(dme)_2_]) are omitted for clarity. Thermal ellipsoids at 50% probability. Selected bond lengths (Å) and bond angles (°) of **3**·[Li(dme)_2_]: B1‐Si1 2.001(2), B1‐Si2 2.014(2), Si1‐N1 1.778(2), Si1‐C6 1.935(2), N2‐C1 1.448(3), C1‐C2 1.465(3), C2‐C3 1.345(3), C3‐C4 1.419(3), C4‐C5 1.365(3), C5‐N1 1.389(2), Si1···Si2 3.504(7), H1A···Li1 1.92(2), H1B···Li1 2.01(2), B1···Li1 2.361(4); Si1‐B1‐Si2 121.6(1), C5‐N1‐C1 113.9(2), N1‐C1‐C2 112.4(2), C1‐C2‐C3 119.1(2), C2‐C3‐C4 119.4(2), C3‐C4‐C5 118.6(2), C4‐C5‐N1 121.9(2)). Selected bond lengths (Å) and bond angles (°) of **4**·[Li(dme)_2_]: B1‐Si1 1.989(3), B1‐Si2 2.006(3), S1‐C7 1.939(3), Si1‐N1 1.777(2), C5‐C6 1.547(4), N1‐C1 1.362(3), C1‐C2 1.353(4), C2‐C3 1.456(5), C3‐C4 1.362(4), C4‐C5 1.500(4), C5‐N1 1.478(4), C6‐C10 1.520(5), C10‐C9 1.366(5), C9‐C8 1.417(5), C8‐N3 1.294(4), N3‐C7 1.464(3), C6‐C7 1.516(4), Si1···Si2 3.544(1), H1A···Li1 2.10(3), H1B···Li1 1.94(2), B1···Li1 2.376(5); Si1‐B1‐Si2 125.0(1), N1‐C5‐C4 108.5(2), C1‐N1‐C5 110.6(2), C7‐C6‐C10 110.1(2), N3‐C8‐C9 126.4(3), N1‐C1‐C2 122.7(3).

All our attempt to address the neutral three‐membered ring corresponding to anionic **2**·[Li(dme)_2_] by adding conventional hydride scavengers such as BH_3_·SMe_2_, MeI, Ph_3_C^+^BF_4_^−^, and Me_3_SiCl resulted in intractable product mixtures. Recently, however, Wagner et al. reported that hydride abstraction from silylborates with Me_3_SiCl is facilitated by the presence of pyridine, which exerts a stabilizing effect through coordination to the free borane.^[^ ^70^ ^]^ Although the application of this protocol to **2**·[Li(dme)_2_] did not result in the anticipated hydride abstraction either, it revealed the surprising formation of pyridine activation products as substantial part of the obtained mixture. Indeed, the reaction of **2**·[Li(dme)_2_] with excess pyridine (in the absence of Me_3_SiCl) at −80 °C, followed by overnight stirring at room temperature led to a clean conversion, resulting in the yellow solid **3**·[Li(dme)_2_] in 64% yield (Scheme 4). The ^11^B NMR spectrum shows a triplet at −41.9 ppm (t, *^1^J_B,H _* = 76.4 Hz), indicating that the two hydrogen atoms remain at a tetracoordinate boron center. The ^29^Si NMR of **3**·[Li(dme)_2_] features two multiplets at 15.0 ppm and −40.4 ppm, suggesting that both silicon atoms remain bonded to the quadrupolar boron nucleus based on their broadening. The considerable downfield shift compared to **2**·[Li(dme)_2_] suggested the opening of the three‐membered ring in line with the high ring strain calculated for **2H**. 2D ^1^H/^29^Si correlation NMR data support the existence of an Si─H functionality by a cross‐peak between the signal at −40.4 ppm and a ^1^H NMR resonance at 5.82 ppm with ^29^Si satellites (*^1^J_Si,H_* = 129.6 Hz). The ^1^H and ^13^C NMR data provide clear evidence for the incorporation of two equivalents of pyridine. ^1^H NMR signals at 8.12, 7.97, 7.60, and 6.61 ppm correspond to four H atoms of a pyridine moiety with retained aromaticity, which based on the loss of one proton must be attached to the B_2_Si scaffold. In contrast, two upfield‐shifted ^1^H NMR doublets at 4.27 and 4.15 ppm (*^2^J_H,H_* = 13.5 Hz) are in line with the transfer of this H atom to the second pyridine equivalent thus transforming it into a nonaromatic dihydropyridine motif with additional vinylic signals at 6.79, 5.96, 5.07, and 4.97 ppm.

Single crystals of **3**·[Li(dme)_2_] suitable for x‐ray analysis were obtained from a concentrated hexane solution at 0 °C. The product **3**·[Li(dme)_2_] crystallizes in the triclinic space group *P2_1_/n* and its molecular structure in the solid state confirms the opening of the BSi_2_ ring of **2**·[Li(dme)_2_] to a linear bis(silyl)borate (Scheme 4). In line with the conclusions drawn from the NMR data, the activation of an *ortho*‐CH bond in one pyridine moiety resulted in the formation of a new Si–C bond to the less encumbered silicon center (Si2─C46 1.935(2) Å). The activated hydrogen is indeed found in the *ortho*‐position of the second pyridine equivalent. The nonplanar ring of the second pyridine with a C2–C1–N1–C5 dihedral angle of 43.57(2)° and the observed bond alternation (N1–C1 1.448(3), C1–C2 1.465(3), C2–C3 1.345(3), C3–C4 1.419(3), C4–C5 1.365(3), C5–N1 1.389(2) Å) convincingly confirm its de‐aromatization. The two B─Si bond lengths of 2.014(2) and 2.001(2) Å are similar to **2**·[Li(dme)_2_] and fall within the range of B─Si single bond lengths.^[^ ^56^, ^57^, ^58^ ^]^ The lithium counter cation of **3**·[Li(dme)_2_] is coordinated to both H atoms of the BH_2_ moiety with Li─H bond distance of 1.92(2) and 2.01(2) Å, matching well with known lithium hydridoborates.^[^ ^67^, ^68^ ^]^

**Figure 4:**
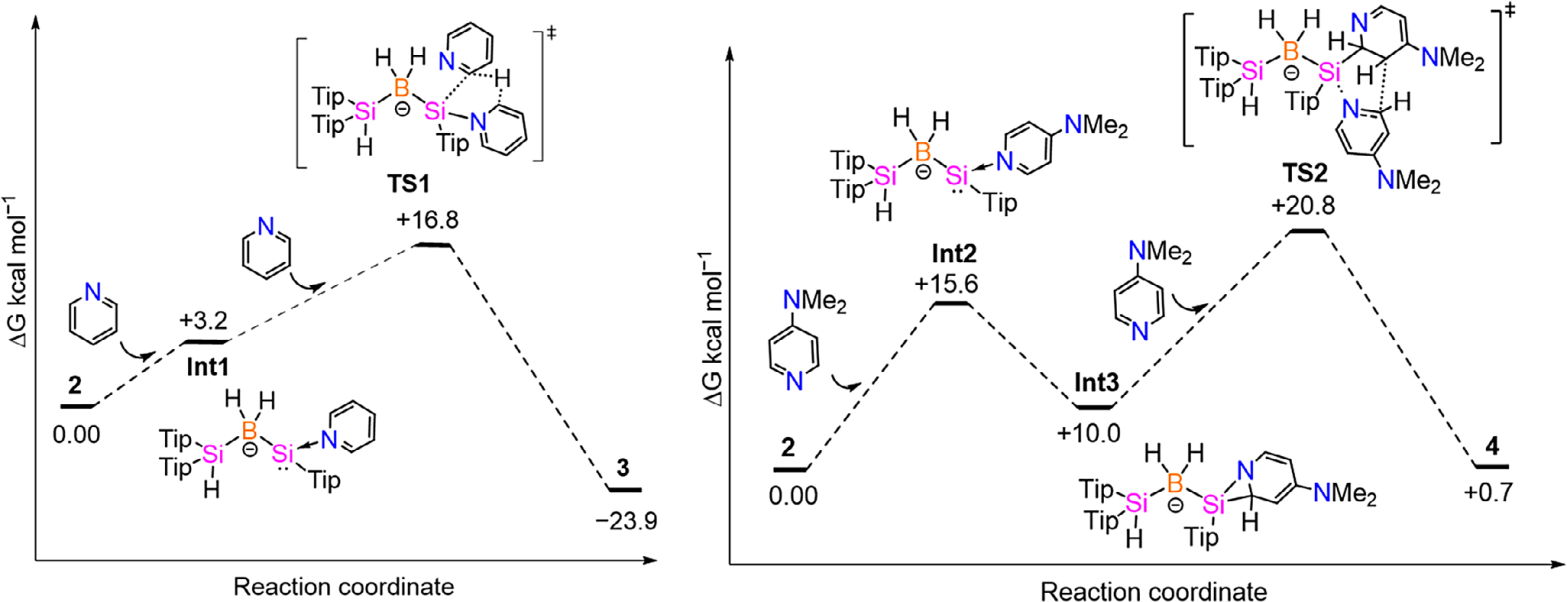
Gibbs free energy (Δ*G* in kcal mol^−1^) reaction coordinate diagrams calculated at the B3LYP‐D3BJ/def2SVP level of theory: Proposed mechanisms for the formation of **3** (left) and **4** (right) from **2** and pyridine and DMAP, respectively.

To gain deeper insight into the reaction mechanism, a deuterated analogue, **3**‐*d*_10_ (Section 1.4), was synthesized with a yield of 30% using pyridine‐*d*_5_. The ^1^H NMR spectrum indicates the complete disappearance of the signals for pyridine and dihydropyridine protons in **3**‐*d*_10_, unambiguously proving hydrogen transfer between the two pyridine moieties without involvement of hydrogen atoms from the BH_2_ or SiH units. The ^2^H NMR spectrum of **3**‐*d*_10_ reveals broad signals within the range of 3.57–7.89 ppm. To further elucidate the formation mechanism of **3**, we carried out DFT studies at the B3LYP‐D3BJ/def2SVP level of theory (Figure 4, left). The reaction is plausibly initiated by pyridine coordination to the less sterically hindered silicon center. The opening of strained three‐membered rings by Lewis bases is well‐established^[^ ^71^, ^72^, ^73^, ^74^, ^75^ ^]^ and would result in a silylene–pyridine adduct in case of **2**. Indeed, **Int1** is at Δ*G* = +3.2 kcal mol^−1^ only marginally higher in free enthalpy. The introduction of another molecule of pyridine initiates the hydrogen transfer from the *ortho* position of first pyridine to the *ortho* position of second pyridine via the transition state **TS1** with an activation barrier of ΔΔ*G* ^#^ = +16.8 kcal mol^−1^. As a result, the second pyridine is dearomatized to form dihydropyridine and ultimately yielding the final product **3**. The overall reaction is exergonic by −23.9 kcal mol^−1^. Selective *ortho*‐CH activation and functionalization of pyridine derivatives are generally facilitated by metal catalysts,^[^ ^76^, ^77^, ^78^, ^79^, ^80^, ^81^, ^82^ ^]^ yet there has been noteworthy progress in metal‐free systems specially with boranes over the past decade.^[^ ^83^ ^]^ Silylene‐triggered C─H bond activation of pyridine in the absence of transition metals, however, has not been reported so far. Tobita et al. described the *ortho*‐metalation of DMAP by a bis(silyl) tungsten complex.^[^ ^84^ ^]^ The Kato and Roesky groups have demonstrated the de‐aromatization of the pyridine ring using low‐valent species.^[^ ^85^, ^86^ ^]^

In view of the initial pyridine coordination in the silylene intermediate **Int1**, we became curious about the effect of a more basic pyridine derivative and hence treated **2**·[Li(dme)_2_] with 2 equivalents of dimethylaminopyridine (DMAP) in toluene at − 80 °C. After stirring at room temperature overnight, product **4**·[Li(dme)_2_] was afforded as a pale‐yellow solid in 61% yield (Scheme 4). ^1^H NMR spectroscopy reveals distinct *N*‐methyl signals at 2.50 and 2.19 ppm corresponding to six hydrogen atoms, confirming the incorporation of two equivalents of the employed pyridine into **4**·[Li(dme)_2_] as in case of **3**·[Li(dme)_2_]. Eight distinct ^1^H NMR signals for CH protons with broadly dispersed chemical shifts at 8.02, 7.78, 7.47, 6.00, 5.30, 4.55, 3.90, and 3.75 ppm (assigned by 2D ^1^H/^13^C correlation spectra) suggested that in contrast to **3**·[Li(dme)_2_] the de‐aromatization of both pyridine moieties had taken place.

The ^11^B NMR shows a pronounced triplet signal at −39.1 ppm (*^1^J_B,H_* = 78.9 Hz) for the two hydrogens at the boron center. The two broad ^29^Si multiplets at 25.4 ppm and − 40.2 ppm are reminiscent of the situation in **3**·[Li(dme)_2_] and in agreement with the retention of both Si─B bonds. The peak at −40.2 ppm indicates a chemical environment that is similar to the Tip_2_SiH moiety in **3**·[Li(dme)_2_] (−40.4 ppm), suggesting the presence of a Si─H moiety in compound **4**·[Li(dme)_2_]. This is confirmed by the 2D ^1^H/^29^Si correlation NMR and the corresponding ^1^H resonance with ^29^Si satellites at 6.15 ppm (*^1^J_Si,H_* = 188.9 Hz).

Single crystals of **4**·[Li(dme)_2_] were grown from a toluene–hexane (1:1) mixture at room temperature and crystallized in the triclinic space group *P* $\bar{1}$ with one molecule of hexane as crystal solvent (Scheme 4). The molecular structure in the solid state confirms the cleavage of the Si─Si bond of the Si_2_B ring just as in the case of **3**·[Li(dme)_2_]. The two B─Si bond lengths of 1.989(3) and 2.006(3) Å are similar to those found in compounds **2**·[Li(dme)_2_] and **3**·[Li(dme)_2_]. The presence of two DMAP‐derived moieties in the crystal structure is in line with the conclusions drawn from the NMR data. Both DMAP rings are dearomatized, as indicated by the endocyclic bonds (C4–C5 1.500(4) Å, C5–N1 1.478(4) Å, N3–C7 1.464(3) Å, C7–C6 1.516(4) Å, and C6–C10 1.520(5) Å), longer than typical aromatic C─C and C─N bonds. The lithium cation is penta‐coordinated by two BH bonds of the borate center, two molecules of 1,2‐dimethoxyethane and one nitrogen from the DMAP moiety. Notably, a new C─C bond is formed between the *ortho*‐ and *meta*‐positions of the two DMAP‐derived moieties with a bond distance of 1.547(4) Å in the typical range for C─C single bonds. Although C─C bond formation between pyridine derivatives is well established in metal‐mediated catalytic^[^ ^87^, ^88^, ^89^, ^90^ ^]^ and stoichiometric^[^ ^91^, ^92^, ^93^, ^94^, ^95^, ^96^, ^97^, ^98^, ^99^, ^100^, ^101^, ^102^, ^103^, ^104^ ^]^ reactions, there are only a few stoichiometric reactions with metal‐free systems.^[^ ^105^, ^106^, ^107^, ^108^, ^109^ ^]^ *Ortho*‐*meta* coupling of two pyridine rings is challenging even for transition metal systems and requires the prefunctionalization of pyridine without exception.^[^ ^87^, ^88^, ^89^, ^90^ ^]^

The formation of compound **4** can be explained by a mechanism that proceeds through **Int2** with relative free energy of Δ*G* = +15.6 kcal mol^−1^ (Figure 4, right), analogous to the formation of **Int1** in case of **3**. The subsequent [1 + 2] cycloaddition between the Si(II) center and the C═N bond of pyridine leads to **Int3,** releasing ΔΔ*G* = −5.6 kcal mol^−1^ in free enthalpy. The highly strained CSiN ring can reasonably be expected to undergo ring‐opening via Si─N bond dissociation due to the introduction of another molecule of DMAP, allowing for a nucleophilic attack from the *meta*‐carbon of the first DMAP on the electrophilic *ortho*‐carbon of the second DMAP. This process can occur through a concerted mechanism, leading to transition state **TS2** by ΔΔ*G* ^#^ = +10.8 kcal mol^−1^ higher in free enthalpy than **Int3**. In this case, a new C─C bond is established between the *ortho*‐ and *meta*‐positions of the two former DMAP moieties in **4**. Despite the dearomatization of both DMAP entities, the overall reaction is only slightly endergonic by +0.7 kcal mol^−1^.

In order to complete the reactivity series with a less Lewis‐basic donor, **2**·[Li(dme)_2_] was treated with pentafluoropyridine (PFP) in toluene at −80 °C. Crystallization from 1:1 toluene‐hexane mixture afforded **5**·[Li(dme)_2_] and an unidentified side product as an inseparable mixture of solids. The ^11^B NMR shows two signals at −33.2 (d, ^1^ *J*_B,H _= 83.9 Hz) and −38.8 (t, ^1^ *J*_B,H _= 89.4 Hz) ppm, indicating the presence of two different isomers containing BH and BH_2_ moieties, respectively.

**Scheme 5:**
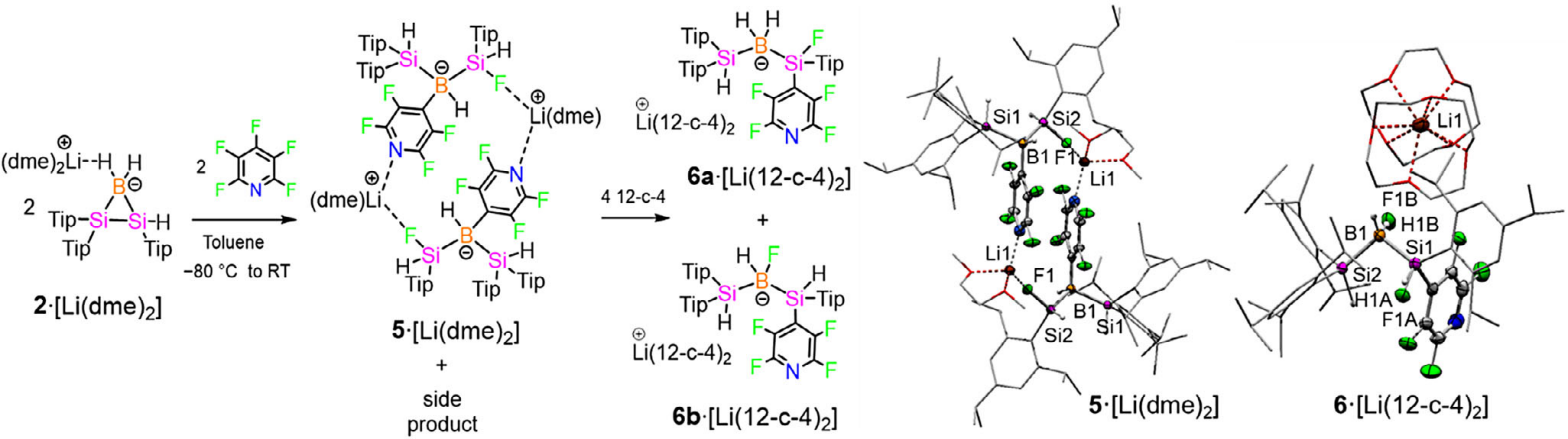
Reactivity of **2**·[Li(dme)_2_] with pentafluoropyridine and molecular structure of **5**·[Li(dme)_2_] and **6**·[Li(12‐c‐4)_2_] in the solid state. Hydrogen atoms and co‐crystallized solvent molecules (toluene and hexane for **6**·[Li(12‐c‐4)_2_]) are omitted for clarity. Thermal ellipsoids at 50% probability. Selected bond lengths (Å) and bond angles (°) of **5**·[Li(dme)_2_]: B1‐Si1 2.048(2), B1‐Si2 1.998(2), Si2‐F1 1.674(1), B1‐C46 1.608(2), F1···Li1 1.826(3), N1···Li1 2.066(3); Si1‐B1‐Si2 108.4(8), B1‐Si2‐F1 107.65(6), Si2‐F1‐Li1 157.2(1), F1···Li1···N1 121.2(1)). Selected bond lengths (Å) and bond angles (°) of **6**·[Li(12‐c‐4)_2_]: B1‐Si1 2.009(3), B1‐Si2 2.037(3), Si1‐F1A 1.606(3), B1‐F1B 1.441(4), Si1‐C16 1.931(3), Li1···F1B 5.611(6), Li1···F1A 9.594(5), Si1···Si2 3.255(1); Si1‐B1‐Si2 107.1(1), Si1‐B1‐F1B 110.4(2), Si1‐B1‐F1B 108.2(2), B1‐Si1‐F1A 112.6(1), B1‐Si1‐C16 104.6(1).

The PFP activation product **5**·[Li(dme)_2_] crystallizes in the monoclinic space group *P*2_1_/*n*. The molecular structure in the solid state (Scheme 5) reveals the opening of the Si_2_B ring through Si─Si bond cleavage, in analogy to **3**·[Li(dme)_2_] and **4**·[Li(dme)_2_]. The activation of the *para*‐CF bond of PFP gives rise to the formation of a B‐C bond under fluorine transfer to the sterically less hindered Si2. The CF‐activation product **5**·[Li(dme)_2_] forms a dimer through intermolecular contacts of Li1···F1 (1.826(3) Å) and Li1···N_PFP_ (2.066(3) Å). Examples of C–F bond activation by low‐valent boron and silicon species are quite well‐known.^[^ ^110^, ^111^, ^112^, ^113^, ^114^, ^115^ ^]^ The Si‐F bond (1.674(1) Å) is slightly elongated compared to reported Si‐F bonds,^[^ ^112^, ^113^, ^114^ ^]^ likely due to the interaction with the Li cation. The two Si‐B bond lengths of 2.048(2) and 1.998(2) Å are matching well with those of **2**·[Li(dme)_2_], **3**·[Li(dme)_2_] and **4**·[Li(dme)_2_].

To address the uncharacterized isomer of the product mixture, we added 12‐crown‐4 (12‐c‐4), expecting the formation of solvent‐separated ion pairs, thus facilitating separation. The ^11^B NMR spectrum displays two signals at − 32.7 (br d, *^1^J_B,H_* = 81.5 Hz) and − 37.8 (t, *^1^J_B,H_* = 86.9 Hz) ppm, once more confirming the presence of BH and BH_2_ units. The ^29^Si NMR spectrum shows four resonances at 30.9,−39.8,−47.2 and −48.8 ppm. The 2D ^1^H‐^29^Si correlation experiments assign the signals at −39.8, −47.2 and −48.8 ppm to the Si─H moieties, which are corroborated in the ^1^H NMR spectrum by resonances with ^29^Si satellites at 5.20 (*^1^J_Si,H_ *= 150.3 Hz), 5.15 (*^1^J_Si,H_ *= 140.3 Hz), and 5.09 (*^1^J_Si,H_ *= 164.4 Hz) ppm. The broad doublet at 30.9 ppm in the ^29^Si spectrum corresponds to the Si─F unit (*^1^J_Si,F_* = 339.4 Hz). The ^19^F NMR spectrum of **6a**·[Li(12‐c‐4)_2_] and **6b**·[Li(12‐c‐4)_2_] shows multiplets at − 98.8 ppm (four aromatic C–F of PFP) and at −127.0 and −131.1 ppm (two aromatic C─F unit of PFP for each), along with a broad singlet at −154.7 ppm, assigned to the Si─F group.

Colorless single crystals suitable for x‐ray analysis were obtained from a 1:1 toluene–hexane mixture. Two regio‐isomers, **6a**·[Li(12‐c‐4)_2_] and **6b**·[Li(12‐c‐4)_2_], co‐crystallize in the monoclinic space group *C*2/*c* as manifest in a positional disorder of the F atom between the boron and the less encumbered silicon center. Furthermore, the tetrafluoropyridinyl group migrated to the latter as well. The lithium counter‐cation is coordinated by two molecules of 12‐c‐4, breaking up the previously dimeric structure. Fluorine and nitrogen centers are well separated from Li1 by 5.611(6) to 9.594(5) Å and 7.995(5) Å, respectively.

The observation of positional changes for both the PFP moiety and the fluorine atom suggests a relatively shallow potential energy surface with facile migratory isomerizations. Due to the large number of involved connectivity changes, we decided against a computational investigation of the multiple mechanistic scenarios but speculate that the initial step may again be the ring opening of **2** by PFP under formation of a base‐stabilized silylene intermediate analogous to **Int1** and **Int2** (Figure 4).

## Conclusion

In conclusion, we have synthesized and fully characterized the first boratadisilirane **2** as its lithium salt. Due to the size mismatch of boron and silicon, the parent species BSi_2_H_6_^−^ is much more strained than its isoelectronic analogues, cyclopropane (C_3_H_6_), boratirane (BC_2_H_6_^−^), and trisilacyclopropane (Si_3_H_6_) as demonstrated by density functional theory (DFT) calculations. The strain imparts a strong propensity for ring opening, paving the way for unprecedented activation modes of pyridine derivatives. In pyridine, the *ortho*‐CH bond is selectively activated by **2**·[Li(dme)_2_] under hydrogen transfer to a second pyridine equivalent, resulting in the formation of a 1,2‐dihydropyridine moiety. In case of DMAP, a new C─C bond is established between the former *ortho* and *meta* positions of two pyridine motifs. For pentafluoropyridine, the *para*‐CF bond is activated selectively, and the fluorine is transferred to the Si and B centers, respectively, giving at least three interconvertible regio‐isomers. The enormous ring strain of the BSi_2_ ring presents significant potential for future investigations into the activation of small molecules and may inspire similar studies with other size‐mismatched small ring systems.

## Supporting Information

The authors have cited additional references within the Supporting Information.^[^ ^54^, ^116^, ^117^, ^118^, ^119^, ^120^, ^121^, ^122^, ^123^, ^124^, ^125^, ^126^, ^127^, ^128^, ^129^, ^130^, ^131^, ^132^, ^133^ ^]^

## Conflict of Interests

The authors declare no conflict of interest.

## Supporting information



Supporting information

## Data Availability

The data that support the findings of this study are available in the supplementary material of this article.
