# Supplementary material for: Ring Strain‐Promoted Activation of Pyridines by a Saturated BSi2 Cycle
Source: Angew Chem Int Ed Engl. 2025 Nov 23;65(1):e17462. doi: 10.1002/anie.202517462 (PMC12759225; doi:10.1002/anie.202517462)
Supplement: Supplementary file 1 — Supporting information [file ANIE-65-e17462-s001.pdf]

Supporting Information

Ring Strain-Promoted Activation of Pyridines by a Saturated BSi_2 Cycle

Nasrina Parvin,[a] Ankur,[a] Philipp Willmes,[a] Bernd Morgenstern,[b] Cem B. Yildiz*[c] and David Scheschkewitz*[a]

[a] Dr. N. Parvin, Ankur, Dr. P. Willmes, Prof. Dr. D. Scheschlewitz - Chair in General and Inorganic Chemistry, Saarland University, 66123 Saarbrücken, Germany

[b] Dr. B. Morgenstern – Service Center X-ray Diffraction, Saarland University, 66123 Saarbrücken, Germany

[c] Prof. Cem Burak Yildiz – Department of Basic Sciences, Faculty of Engineering, Architecture and Design, Bartin University, 74100 Bartin, Turkey

E-Mails: scheschkewitz@mx.uni-saarland.de; cbyildiz@bartin.edu.tr

Table of contents

1. **Synthetic methods, analytical data and spectra** **3**
   1.1 General considerations 3
   1.2 Synthesis and characterization of **2**·[Li(dme)$_2$] 4
   1.3 Synthesis and characterization of **3**·[Li(dme)$_2$] 9
   1.4 Synthesis and characterization of **3**-d_{10} 13
   1.5 Synthesis and characterization of **4**·[Li(dme)$_2$] 17
   1.6 Synthesis and characterization of **5**·[Li(dme)$_2$] 20
   1.7 Synthesis and characterization of **6a**·[Li(dme)$_2$] and **6b**·[Li(dme)$_2$] 21

2. **Crystallographic data** **27**
   2.1 Crystal structure of **2**·[Li(dme)$_2$] 27
   2.2 Crystal structure of **3**·[Li(dme)$_2$] 29
   2.3 Crystal structure of **4**·[Li(dme)$_2$] 31
   2.4 Synthesis and characterization of **5**·[Li(dme)$_2$] 33
   2.5 Synthesis and characterization of **6a**·[Li(dme)$_2$] and **6b**·[Li(dme)$_2$] 35

3. **Computational Details** **37**
   3.1 Optimization of **2** 37
   3.2 Selected molecular orbitals of **2** 41
   3.3 Natural bond orbital (NBO) analysis of **2** 41
   3.4 Topological Study of **2** 43
   3.5 Ring Strain Energy Calculation 44
   3.6 Mechanism for the formation of **3** and IRC Plot 51
   3.7 Optimization of **3** 52
   3.8 Optimization of **Int1** 55
   3.9 Optimization of **TS1** 59
   3.10 Mechanism for the formation of **4** 64
   3.11 Optimization of **4** 64
   3.12 Optimization of **Int2** 67
   3.13 Optimization of **Int3** 70
   3.14 Optimization of **TS2** 73

4. **References** 76

1. Synthetic methods, analytic data and spectra

1.1 General considerations

All manipulations were carried out under a protective atmosphere of argon applying standard Schlenk or glovebox techniques. The glassware was pre-dried in oven at 125 °C and heated in vacuo prior to use. Solvents were dried and degassed by reflux over sodium/benzophenone under argon (thf, toluene) or taken from a solvent purification system (Innovative technology PureSolv MD7; diethylether, hexane, pentane). C_6D_6 was dried over potassium mirror and distilled under argon prior to use. **1**·[Li(dme)$_2$][S1] was prepared according to the published procedures. All other chemicals were obtained commercially and used as received. The solution NMR spectra were recorded on a Bruker Avance III HD 400 spectrometer at 300K (^{1}H: 400.13 MHz, ^{13}C: 100.61 MHz, ^{29}Si: 79.49, ^{7}Li: 155.50 MHz). The ^{1}H and ^{13}C{^{1}H} NMR spectra were referenced to the residual proton and natural abundance. ^{13}C resonances of the deuterated solvent and chemical shifts were reported relative to $SiMe_4$ (thf-d_8: δ ^{1}H = 1.72, 3.58 ppm and δ ^{13}C = 67.21, 25.31 ppm).[S2] The ^{29}Si NMR chemical shifts were referenced to external $SiMe_4$. The following abbreviations were used for the multiplicities: s – singlet, d – doublet, t – triplet, sept – septet, m – multiplet, br s – broad singlet. UV/Vis spectra were recorded on a Shimdazu UV-2600 spectrometer in quartz cells with a path length of 0.1 cm. Melting points were determined under argon in sealed NMR tubes and are uncorrected. The molten samples were examined by NMR spectroscopy to confirm whether decomposition had occurred upon melting. Elemental analyses were performed in triplicate for each sample using Leco CHN900 analyzer and mean values are given below for each compound. Crystallographic data of the structures reported in this paper have been deposited with the Cambridge Crystallographic Data Centre, CCDC, 12 Union Road, Cambridge CB21EZ, UK. Copies of the data can be obtained free of charge on quoting the depository numbers CCDC - 2474811 (**2**·[Li(dme)$_2$), CCDC - 2474816 (**3**·[Li(dme)$_2$), CCDC - 2474821 (**4**·[Li(dme)$_2$]), CCDC – 2489523 (**5**·[Li(dme)$_2$]), CCDC – 2489526 (**6**·[Li(dme)$_2$]). (Fax: +44-1223-336-033; E-Mail: deposit@ccdc.cam.ac.uk, http://www.ccdc.cam.ac.uk). (Fax: +44-1223-336-033; E-Mail: deposit@ccdc.cam.ac.uk, http://www.ccdc.cam.ac.uk).

1.2 Synthesis of 2·[Li(dme)$_2$]:

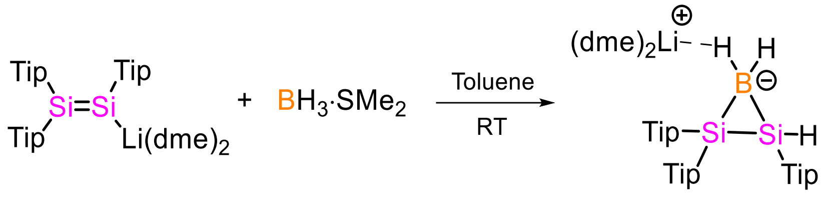

1·[Li(dme)$_2$] **2**·[Li(dme)$_2$]

853.38 g/mol 75.96 g/mol 867.23 g/mol

BH_3·SMe_2 (57 µL, 0.6 mmol) is added dropwise to the solution of **1**·[Li(dme)$_2$] (512 mg, 0.6 mmol) in toluene at room temperature with vigorous stirring. The color of the reaction mixture changes from orange to yellow immediately. The reaction is stirred for 1 hour, evaporated to dryness and extracted in hexane. After filtration, the solution is concentrated to saturation at reduced pressure and kept at -30 °C for three days to yield yellow-colored crystals of **2**·[Li(dme)$_2$] (370 mg, 71%).

^{1}H NMR (400.13 MHz, C_6D_6, 300 K): δ = 7.20 (s, 2H, Ph-*H*), 7.14 (s, 2H, Ph-*H*), 7.02 (s, 2H, Ph-*H*), 4.77 to 4.86 (m, 4H, C*H*(CH$_3$)$_2$), 4.20 to 4.27 (m, 2H, C*H*(CH$_3$)$_2$), 3.95 (m, 1H, $^1J_{SiH}$ = 178.9 Hz, Si*H*), 2.73 to 2.91 (m, 3H, C*H*(CH$_3$)$_2$), 2.71 (s, 12H, O-C*H*$_{3,DME}$), 2.54 (s, 8H, C*H*$_{2,DME}$), 1.53 (d, 6H, $^3J_{HH}$ = 6.7 Hz, CH(C*H*$_3$)$_2$), 1.47 (d, 6H, $^3J_{HH}$ = 6.8 Hz, CH(C*H*$_3$)$_2$), 1.36 (d, 6H, $^3J_{HH}$ = 6.6 Hz, CH(C*H*$_3$)$_2$), 1.32 (d, 6H, $^3J_{HH}$ = 6.7 Hz, CH(C*H*$_3$)$_2$), 1.30 (d, 3H, $^3J_{HH}$ = 6.8 Hz, CH(C*H*$_3$)$_2$), 1.29 (d, 3H, $^3J_{HH}$ = 6.9 Hz, CH(C*H*$_3$)$_2$), 1.26 (d, 3H, $^3J_{HH}$ = 6.8 Hz, CH(C*H*$_3$)$_2$), 1.25 (d, 3H, $^3J_{HH}$ = 6.8 Hz, CH(C*H*$_3$)$_2$), 1.19 (d, 3H, $^3J_{HH}$ = 6.9 Hz, CH(C*H*$_3$)$_2$), 1.18 (d, 3H, $^3J_{HH}$ = 6.9 Hz, CH(C*H*$_3$)$_2$), 1.16 (d, 3H, $^3J_{HH}$ = 6.5 Hz, CH(C*H*$_3$)$_2$), 0.78 (d, 6H, $^3J_{HH}$ = 6.6 Hz, CH(C*H*$_3$)$_2$), ppm. Note: The signals for BH_2 moiety could not be located, presumably due to the broadening caused by coupling to the quadrupolar boron nuclei.

^{13}C{^{1}H} NMR (100.61 MHz, C_6D_6, 300 K): δ = 156.8, 155.6, 155.2, 148.4, 147.8, 147.8, 139.4, 138.3, 132.5 (each s, Ph-*C*), 120.7, 120.0 (each s, Ph-CH), 69.3 (*C*H$_{2,DME}$), 58.8 (*C*H$_{3,DME}$), 35.4, 34.5, 34.3, 34.3, 34.1, 33.9 (each s, *C*H(CH$_3$)$_2$), 25.6, 25.4, 25.2, 24.7, 24.7, 24.5, 24.1, 24.0, 24.0, 23.9, (each *s*, CH(*C*H$_3$)$_2$) ppm.

^{11}B NMR (128.38 MHz, C_6D_6, 300 K): δ = −37.9 (t, *J* = 99.1 Hz) ppm.

^{29}Si{^{1}H} NMR (79.49 MHz, C_6D_6, 300 K): δ = −82.1 (br), −102.8 (br) ppm.

^{7}Li NMR (155.50 MHz, C_6D_6, 300K): δ = 0.4 ppm.

Elemental Analysis: Calcd. for $C_{59}H_{106}BSi_2LiO_4$, [953.41] (including hexane): C, 74.33; H, 11.21. Found: C, 75.14; H, 10.32.

Mp: 138 - 140 °C (decomposed).

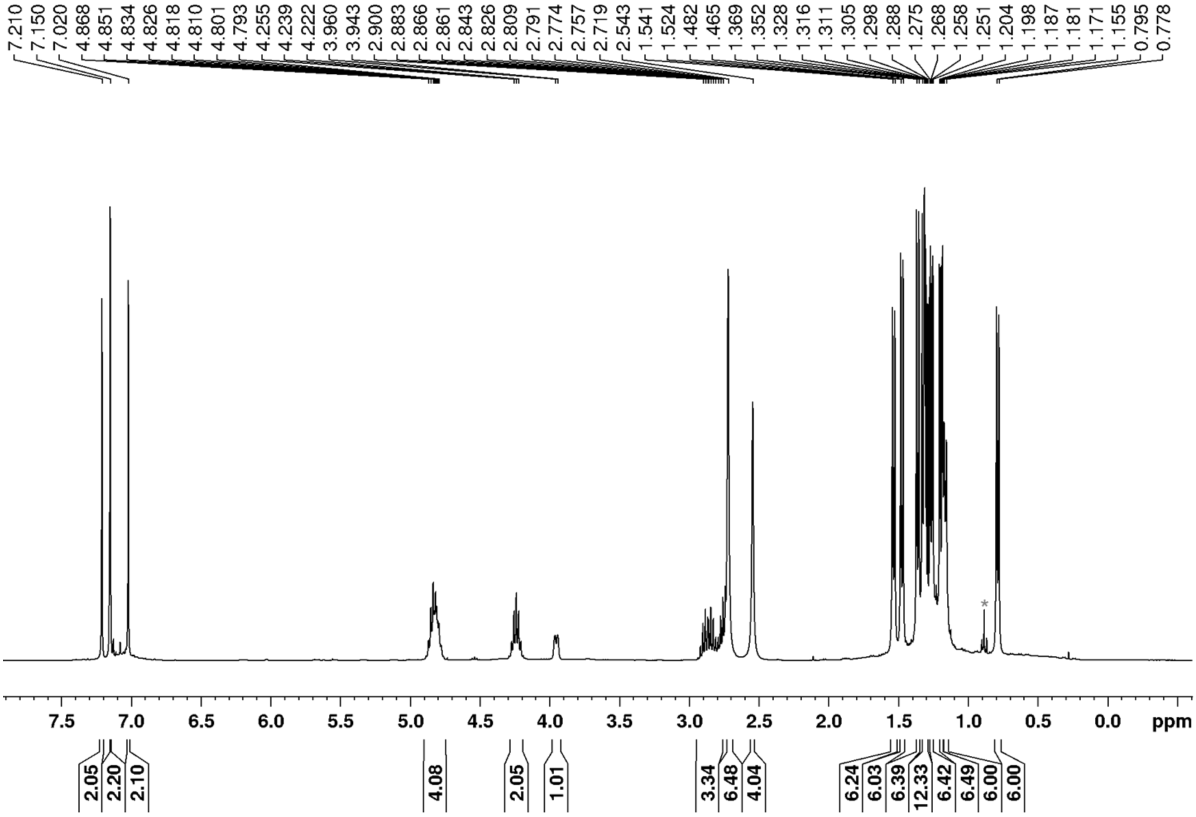

Fig S1: ^{1}H NMR of **2**·[Li(dme)$_2$] in C_6D_6 at 300K (* = hexane).

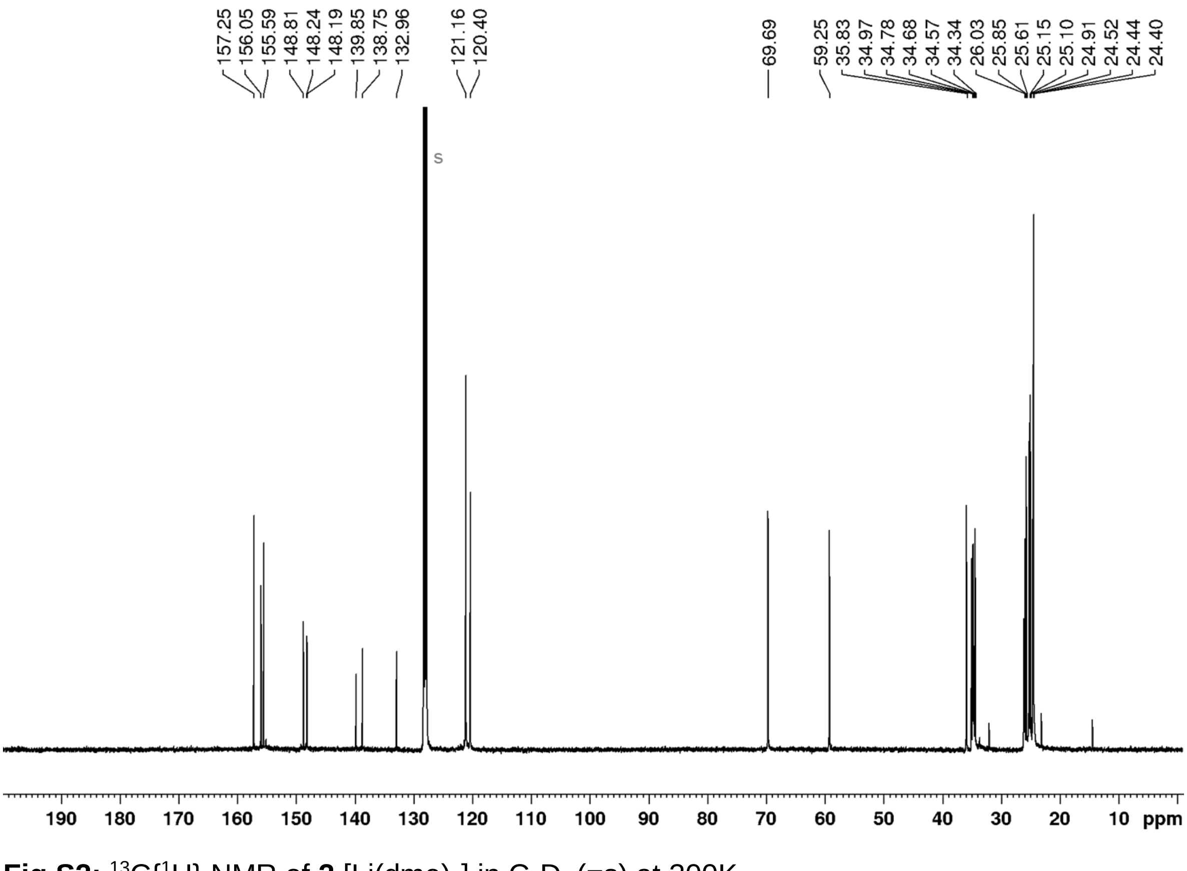

Fig S2: ^{13}C{^{1}H} NMR of **2**·[Li(dme)$_2$] in C_6D_6 (=s) at 300K.

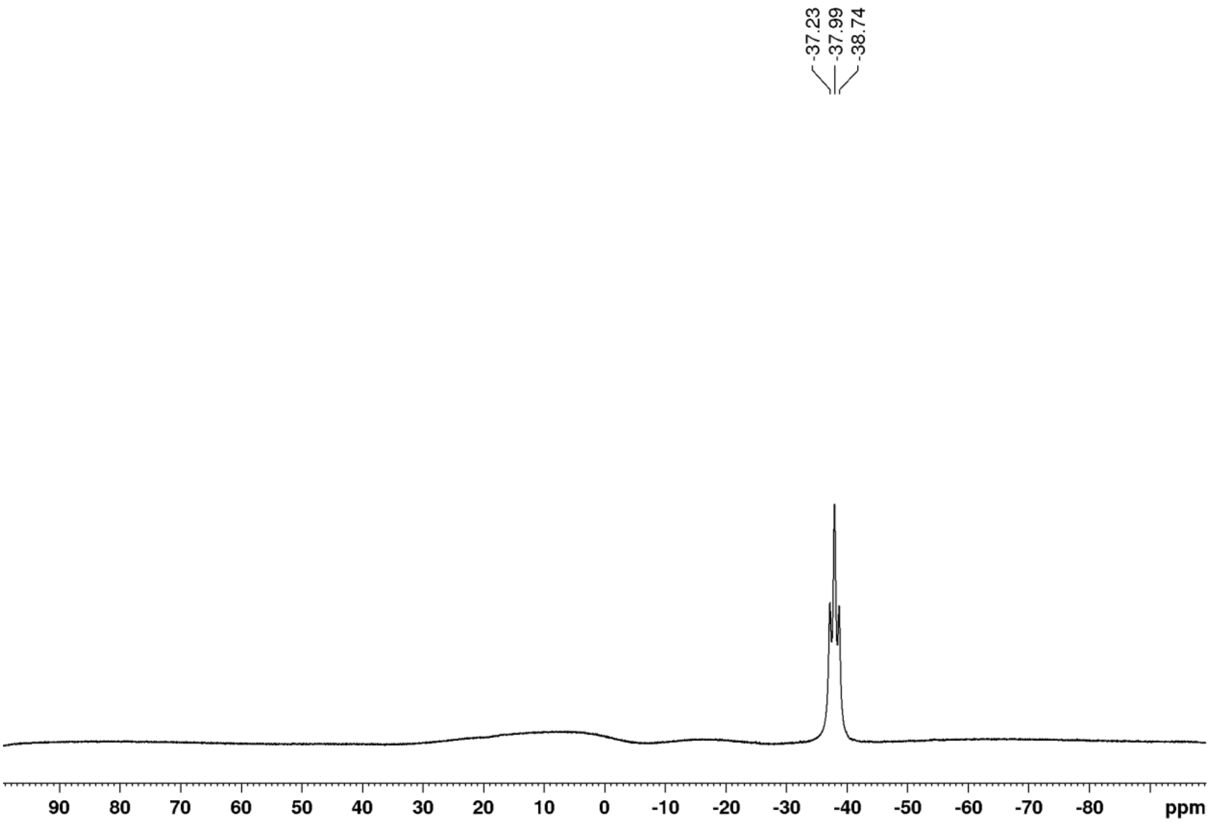

Fig S3: ^{11}B NMR of **2**·[Li(dme)$_2$] in C_6D_6 at 300K

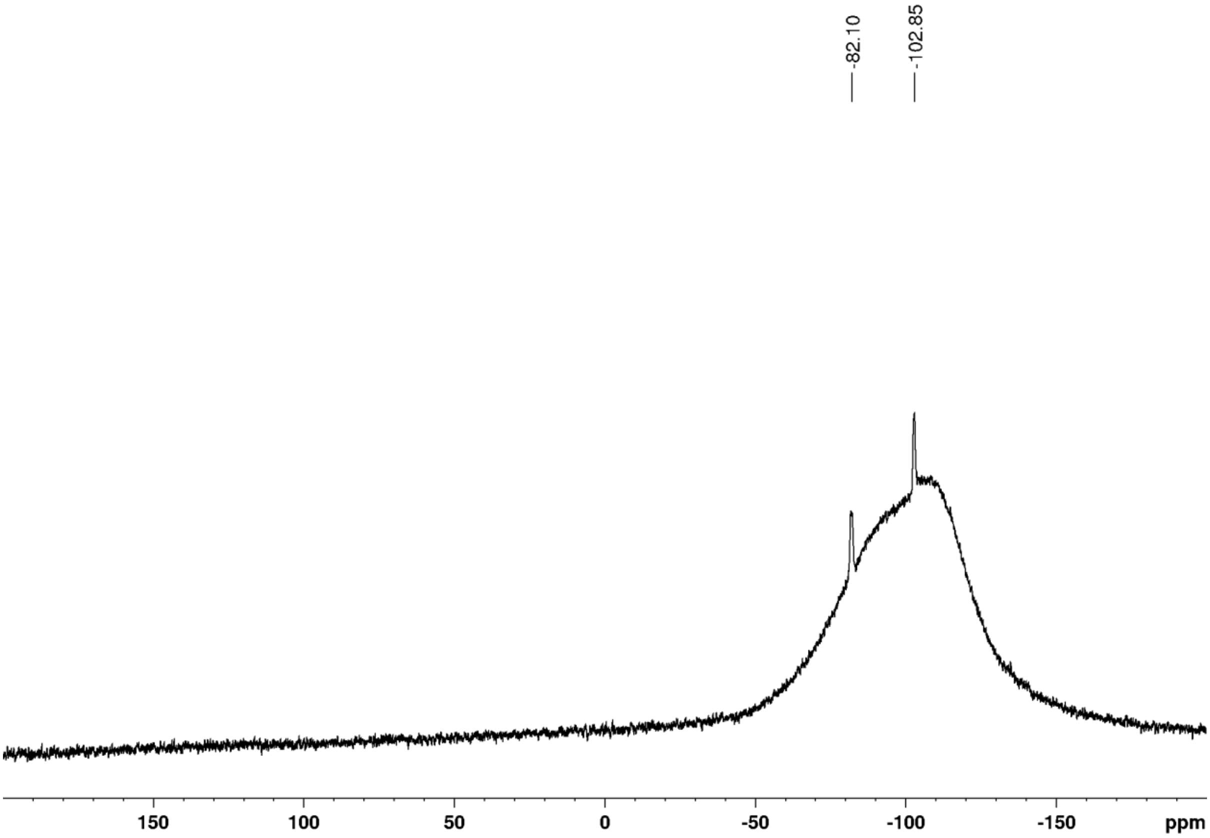

Fig S4: $^{29}Si\{^1H\}$ NMR of **2**·[Li(dme)$_2$] in C_6D_6 at 300K.

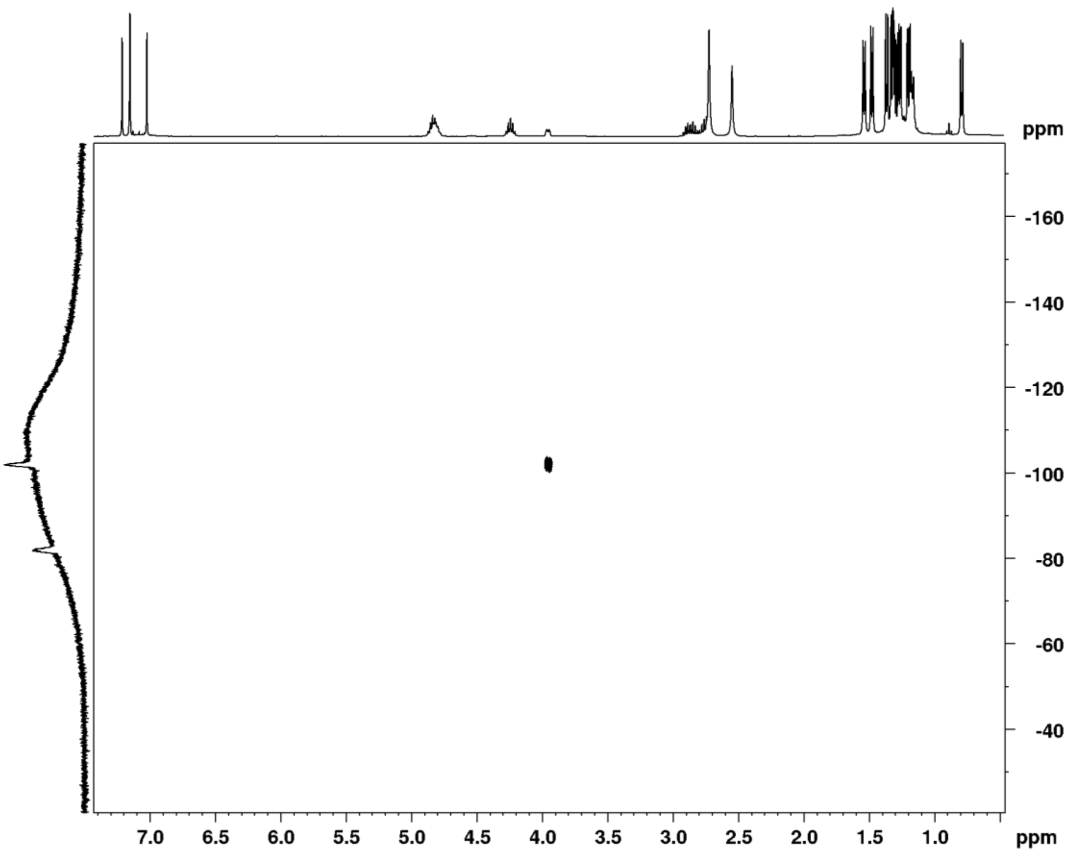

Fig S5: ^{1}H- ^{29}Si NMR of **2**·[Li(dme)$_2$] in C_6D_6 at 300K.

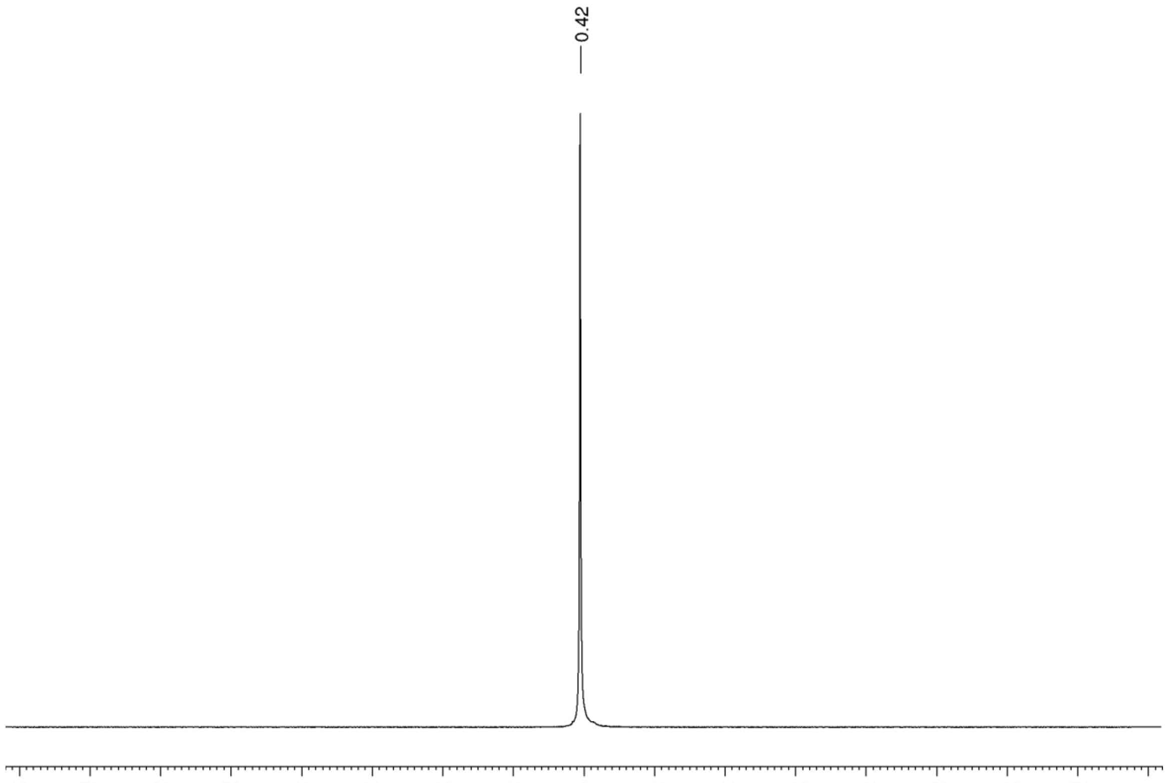

Fig S6: ^{7}Li NMR of **2**·[Li(dme)$_2$] in C_6D_6 at 300K.

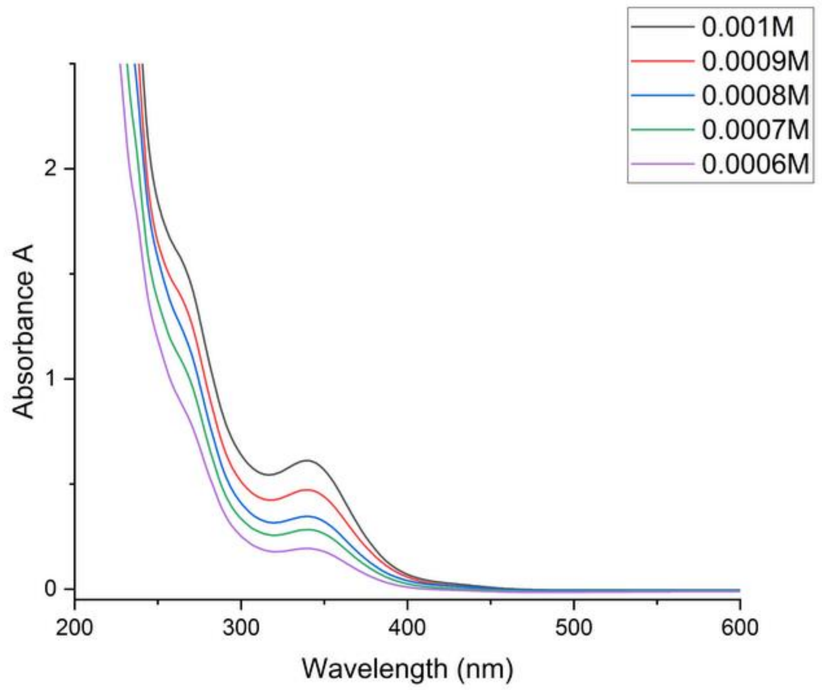

Figure S7. UV-Vis spectra of **2**·[Li(dme)$_2$] in hexane at different concentrations.

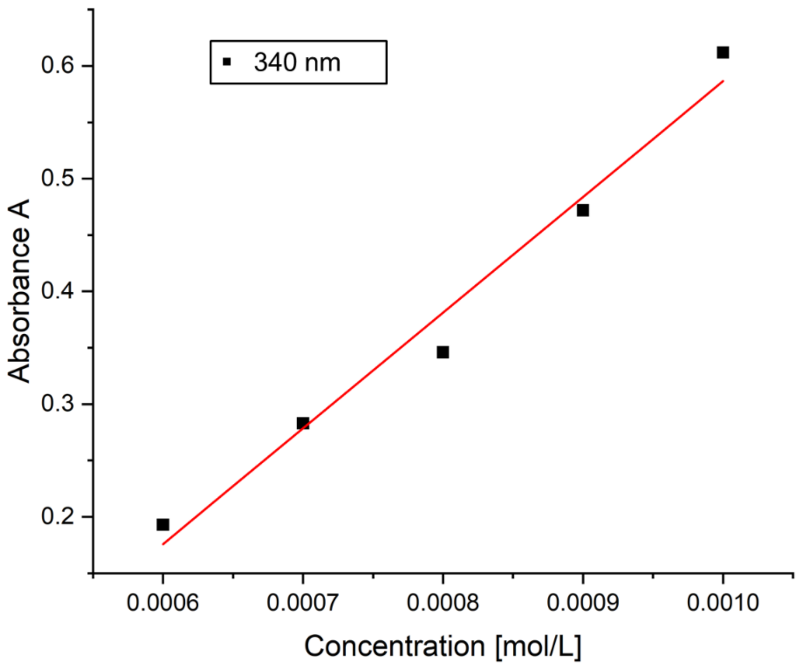

Figure S8. Determination of the extinction coefficient ε = 10270 L mol^{-1} cm^{-1} of **2**·[Li(dme)$_2$] by linear regression at λ_{max} = 340 nm

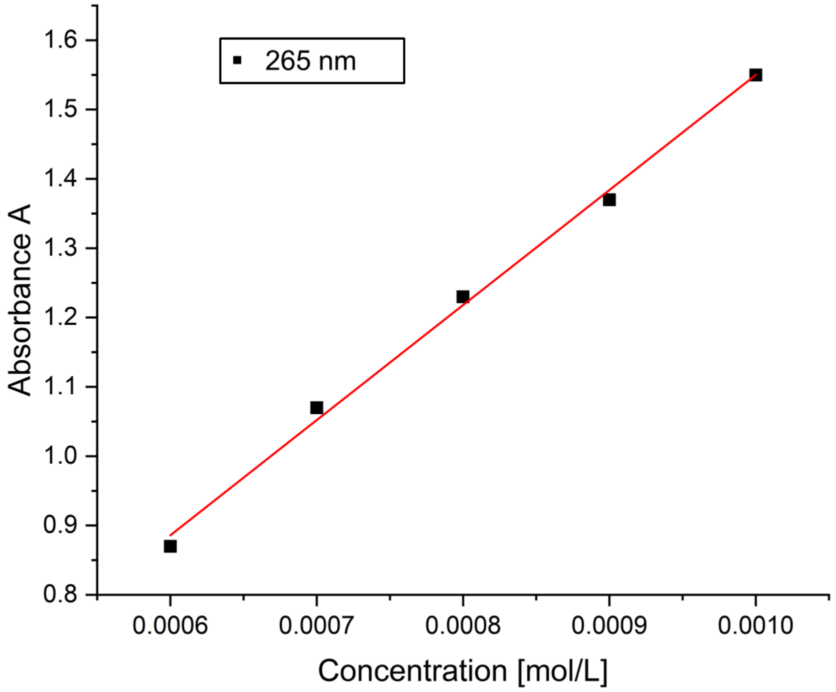

Figure S9. Determination of the extinction coefficient ε = 16600 L mol^{-1} cm^{-1} of **2**·[Li(dme)$_2$] by linear regression at λ_{max} = 265 nm

1.3 Synthesis of 3·[Li(dme)$_2$]:

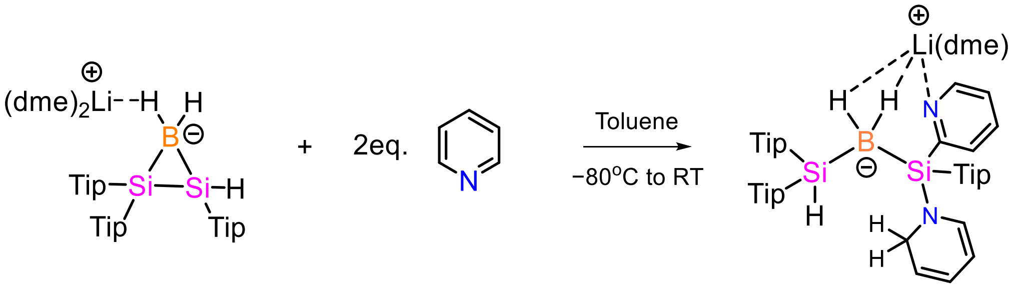

2·[Li(dme)$_2$]
867.23 g/mol

79.1 g/mol

3·[Li(dme)$_2$]
935.32 g/mol

2·[Li(dme)$_2$] (434 mg, 0.5 mmol) is dissolved in toluene and pyridine (89 µL, 2.1 mmol) is added at −80 °C with vigorous stirring. The reaction mixture is stirred overnight at room temperature. After filtration, the toluene solution is evaporated to dryness and a minimum amount of hexane is added to dissolve the product. Pale yellow crystals of **3**·[Li(dme)$_2$] (300 mg, 64%) are obtained at 0 °C after 2 days.

^{1}H NMR (400.13 MHz, C_6D_6, 300 K): δ = 8.12 (br s, 1H, $CH_{pyridine}$), 7.97 (d, 1H, $^3J_{HH}$ = 5.1 Hz, $CH_{pyridine}$), 7.60 (br, 1H, $CH_{pyridine}$), 7.20 (s, 2H, CH_{Ph}), 7.08 (*s*, 2H, CH_{Ph}), 7.06 (s, 2H, CH_{Ph}), 6.79 (d, 1H, $^3J_{HH}$ = 6.9 Hz, $CH_{dihydropyridine}$), 6.61 (ddd, 1H, J = 1.3, 5.0 and 7.5 Hz, $CH_{pyridine}$), 5.96 (dd, 1H, J = 5.2 and 9.1 Hz, $CH_{dihydropyridine}$), 5.82 (br s, 1H, SiH), 5.07 (t, 1H, J = 6.2 Hz,

C$H_{dihydropyridine}$), 4.97 (br s, 1H, C$H_{dihydropyridine}$), 4.27 (br d, 1H, $^{2}J_{HH}$ = 13.5 Hz, C$H_{2,dihydropyridine}$), 4.15 (br d, 1H, $^{2}J_{HH}$ = 13.5 Hz, C$H_{2,dihydropyridine}$, merged), 4.14 (br, 1H, C*H*(CH_3)$_2$, merged), 3.91 (br, 2H, C*H*(CH_3)$_2$), 3.67 to 3.62 (m, 2H, C*H*(CH_3)$_2$), 2.86 to 2.75 (m, 4H, C*H*(CH_3)$_2$), 2.71 (s, 4H, C$H_{2,DME}$, merged), 2.70 (s, 6H, C$H_{3,DME}$, merged), 1.43 (br s, 6H, CH(CH_3)$_2$), 1.26, 1.25 (d, 21H, $^{3}J_{HH}$ = 6.8 Hz, CH(CH_3)$_2$, merged), 1.22 (d, 12H, $^{3}J_{HH}$ = 6.8 Hz, CH(CH_3)$_2$), 1.15 to 1.13 (br d, $^{3}J_{HH}$ = 6.6 Hz, 6H, CH(CH_3)$_2$), 1.10 (br d, $^{3}J_{HH}$ = 6.2 Hz, 6H, CH(CH_3)$_2$), 0.95 (br s, 3H, CH(CH_3)$_2$), ppm. Note: The signals for BH_2 moiety could not be identified due to broadening.

^{13}C{^{1}H} NMR (100.61 MHz, C_6D_6, 300 K): δ = 177.9 (*s*, $C_{pyridine}$), 156.9, 155.0, 154.6, 149.8, 149.3, 147.9, 147.5 (each *s*, CH_{Ph}), 147.1 (*s*, $CH_{pyridine}$), 140.7 (*s*, $CH_{dihydropyridine}$), 139.2, 136.7 (*s*, C_{Ph}), 134.6 (*s*, $CH_{pyridine}$), 131.4 (*s*, $CH_{pyridine}$), 125.5 (*s*, $CH_{dihydropyridine}$), 124.1 (*s*, $CH_{pyridine}$), 122.1, 121.4, 121.4, 121.1 (each s, CH_{Ph}), 109.1 (*s*, $CH_{dihydropyridine}$), 99.7 (*s*, $CH_{dihydropyridine}$), 69.9 (*s*, $CH_{2,DME}$), 58.8 (*s*, $CH_{3,DME}$), 45.6 (*s*, $CH_{2,dihydropyridine}$), 34.7, 34.6, 33.2 (each *s*, *C*H(CH_3)$_2$), 31.9 ($CH_{2,hexane}$), 26.3, 26.1, 25.4, 25.2, 24.9, 24.4, 24.4, 24.3, 24.3, 24.2, 24.2, 24.1 (each *s*, CH(CH_3)$_2$), 23.0 ($CH_{2,hexane}$), 14.3 ($CH_{3,hexane}$) ppm.

^{11}B NMR (128.38 MHz, C_6D_6, 300 K): δ = −41.9 (*t*, $^{1}J_{BH}$ = 76.4 Hz) ppm.

^{29}Si{^{1}H} NMR (79.49 MHz, C_6D_6, 300 K): δ = 16.0 to 13.0 (m), −39.6 to −42.2 (m) ppm.

^{7}Li NMR (155.50 MHz, C_6D_6, 300K): δ = 2.8 (*s*) ppm.

Elemental Analysis: Calcd. for $C_{69}H_{116}BSi_2LiN_2O_4$, [1111.62] (including hexane): C, 74.55; H, 10.52; N, 2.52. Found: C, 75.02; H, 8.59; N, 3.13.

Mp: 192 - 195 °C (decomposed).

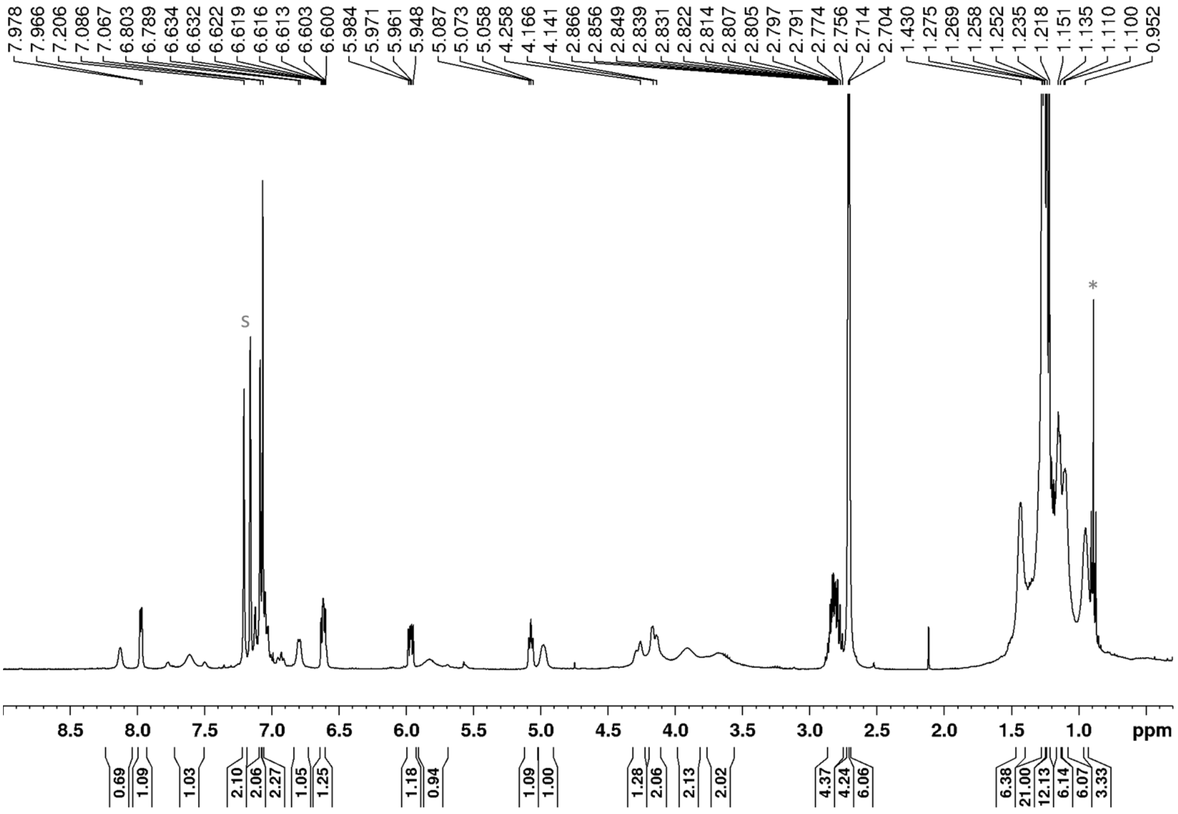

Fig S10: ^{1}H NMR of **3**·[Li(dme)$_2$] in C_6D_6 (= s) at 300K (* = hexane).

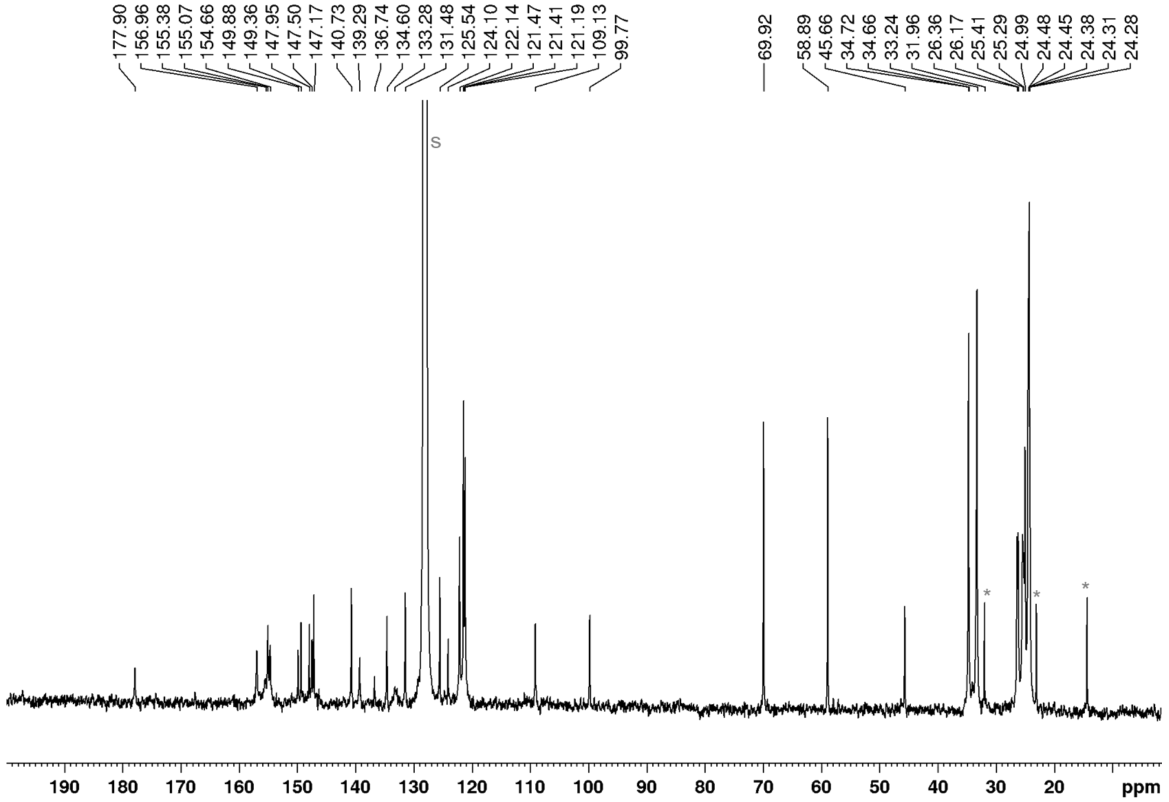

Fig 11: ^{13}C NMR of **3**·[Li(dme)$_2$] in C_6D_6 (= s) at 300K (* = hexane).

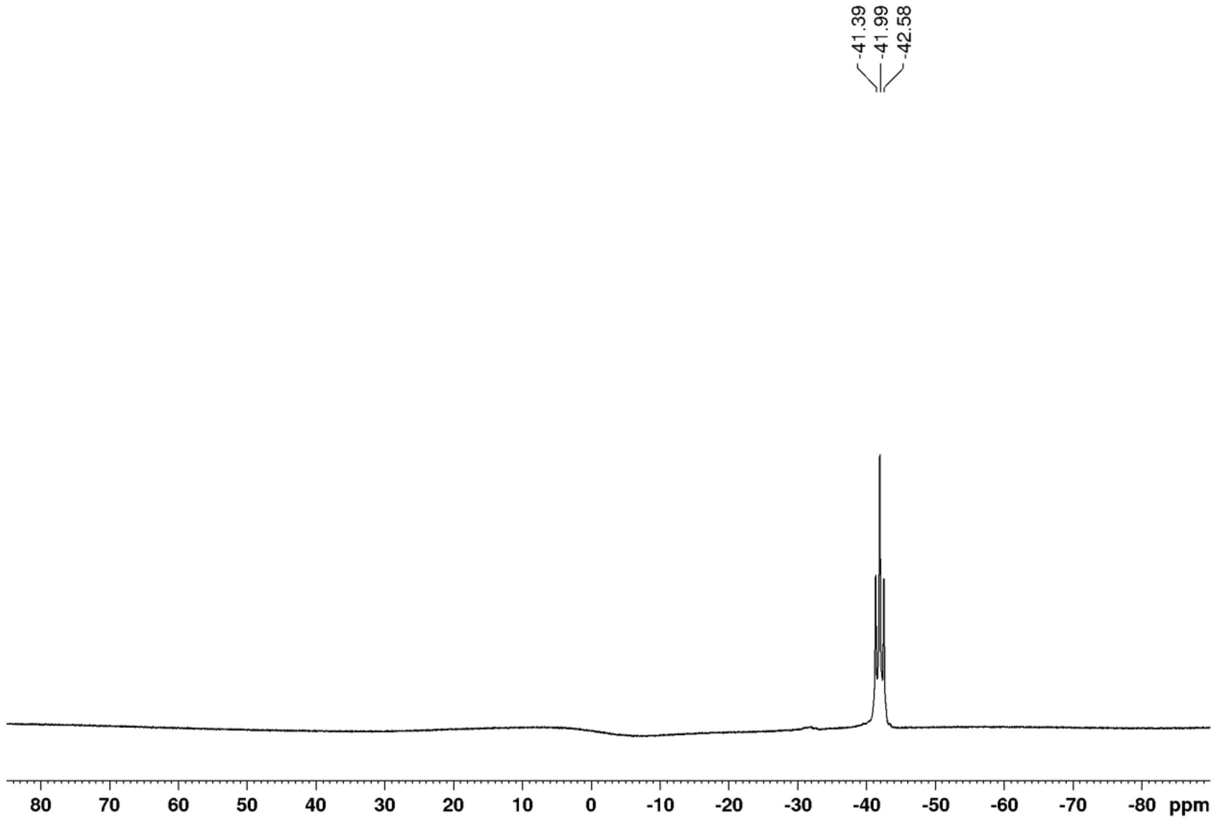

Fig S12: ^{11}B NMR of **3**·[Li(dme)$_2$] in C_6D_6 at 300K

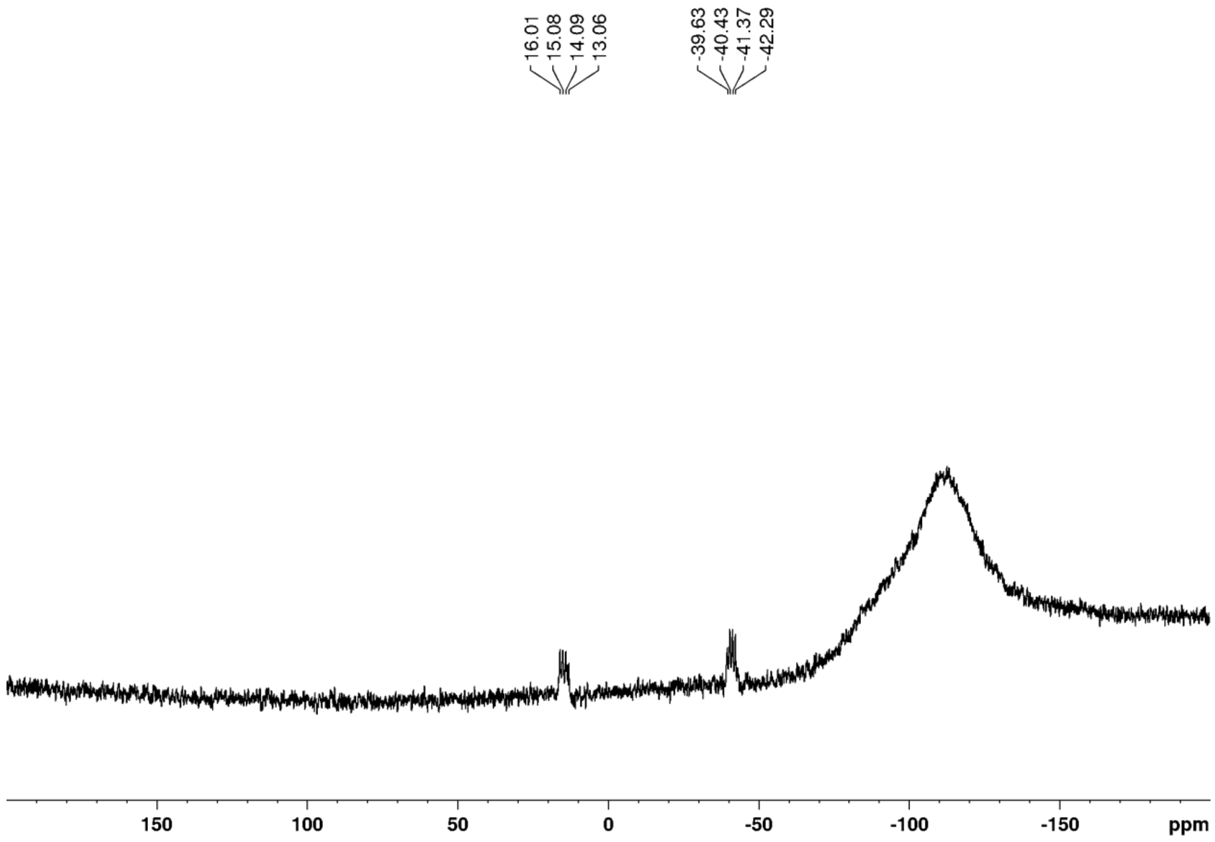

Fig S13: ^{29}Si NMR of **3**·[Li(dme)$_2$] in C_6D_6 at 300K

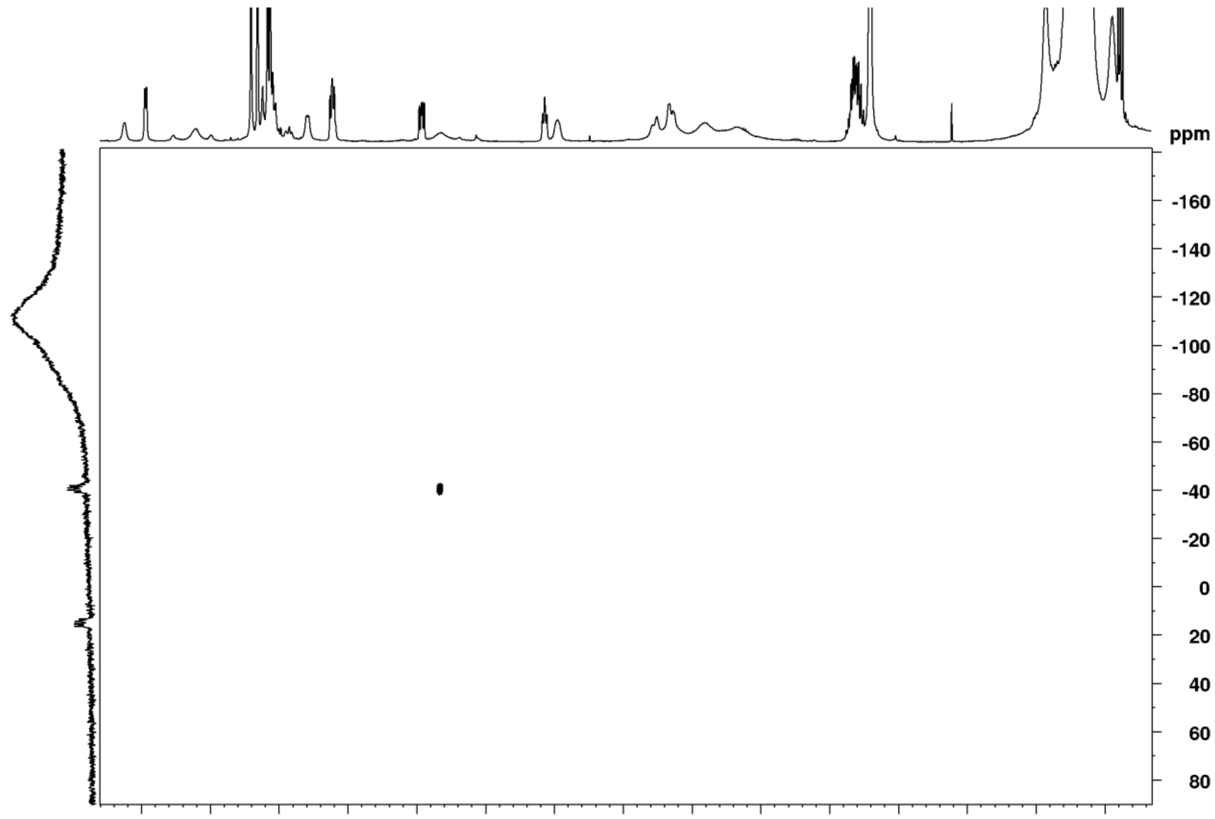

Fig S14: ^{29}Si-^{1}H HMQC NMR of **3**·[Li(dme)$_2$] in C_6D_6 at 300K

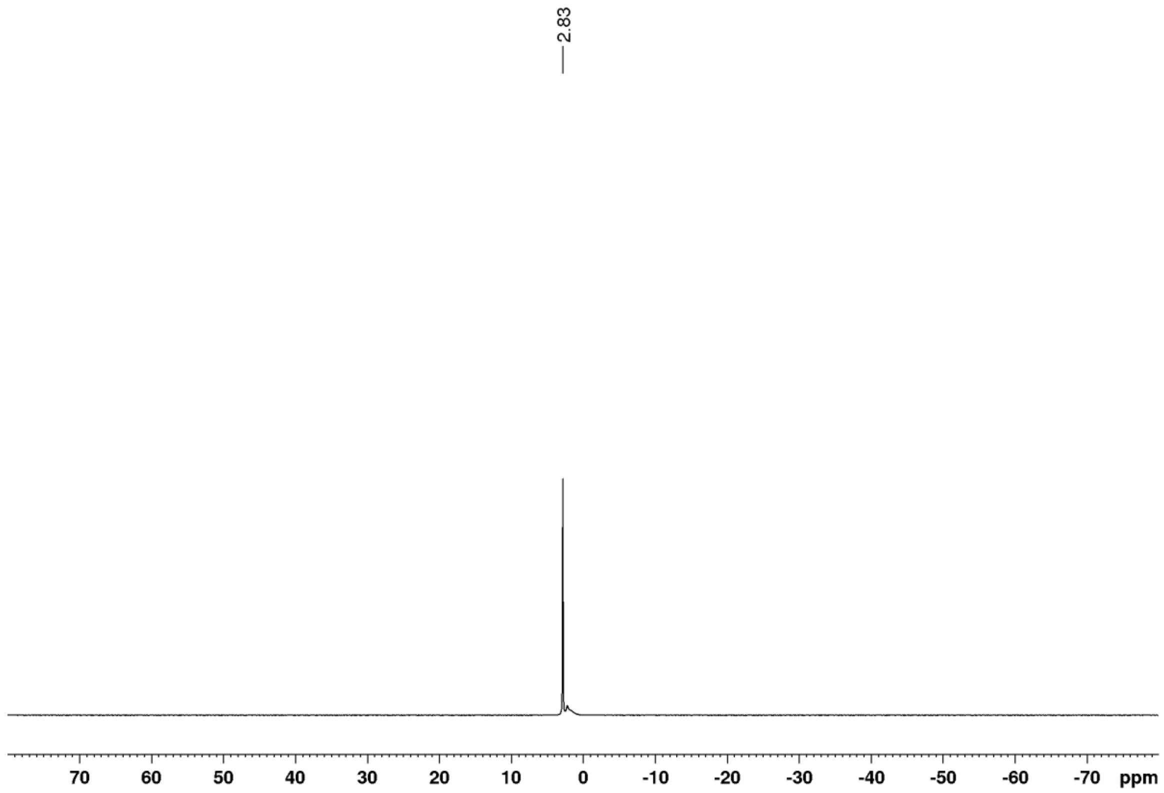

Fig S15: ^{7}Li NMR of **3**·[Li(dme)$_2$] in C_6D_6 at 300K

1.4 Synthesis of 3-*d*$_{10}$:

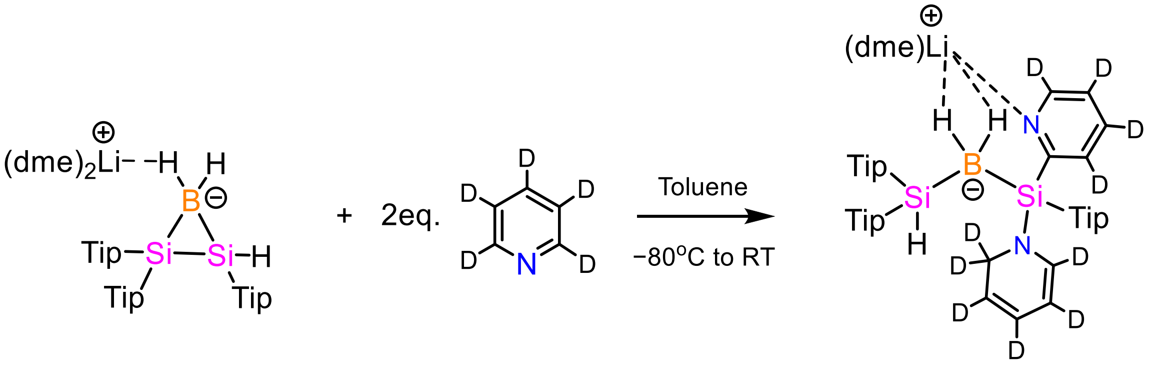

2·[Li(dme)$_2$] (300 mg, 0.345 mmol) is dissolved in toluene and pyridine-*d*$_5$ (58 µL, 2.1 mmol) is added at −80 °C with vigorous stirring. The reaction mixture is stirred overnight at room temperature. After filtration, all volatiles are evaporated in vacuum and a minimum amount of hexane is added to solubilize the product. Pale yellow crystals of **3**-*d*$_{10}$ (100 mg, 30%) are obtained at 0 °C after 2 days.

^{1}H NMR (400.13 MHz, C_6D_6, 300 K): δ = 7.20 (s, 2H, CH_{Ph}), 7.08 (*s*, 2H, CH_{Ph}), 7.06 (s, 2H, CH_{Ph}), 5.82 (br s, 1H, Si*H*), 4.15 (br, 1H, C*H*(CH$_3$)$_2$, merged), 3.91 (br, 2H, C*H*(CH$_3$)$_2$), 3.72 to 3.56 (m, 2H, C*H*(CH$_3$)$_2$), 2.87 to 2.76 (m, 4H, C*H*(CH$_3$)$_2$), 2.72 (s, 4H, C$H_{2,DME}$, merged), 2.71 (s, 6H, C$H_{3,DME}$, merged), 1.43 (br d, 6H, $^3J_{HH}$ = 6.1 Hz, CH(CH_3)$_2$), 1.26, 1.25 (d, 21H,

$^3J_{HH}$ = 6.9 Hz, CH(CH_3)$_2$, merged), 1.22 (d, 12H, $^3J_{HH}$ = 6.8 Hz, CH(CH_3)$_2$), 1.15 to 1.15 (br d, $^3J_{HH}$ = 6.4 Hz, 6H, CH(CH_3)$_2$), 1.10 (br s, 6H, CH(CH_3)$_2$), 0.94 (br s, 3H, CH(CH_3)$_2$), ppm. Note: The signals for BH_2 moiety could not be identified due to broadening.

^{2}H NMR (61.42 MHz, C_6H_6, 300 K): δ = 7.89 (br), 7.12 (br), 6.65 (br), 6.28 (br), 6.01 (br), 5.42 (br), 4.42 (br), 3.57 (br) ppm.

^{11}B NMR (128.38 MHz, C_6D_6, 300 K): δ = −41.9 (*t*, $^1J_{BH}$ = 76.6 Hz) ppm.

^{29}Si{^{1}H} NMR (79.49 MHz, C_6D_6, 300 K): δ = 16.7 to 13.8 (m), −39.4 to −42.2 (m) ppm.

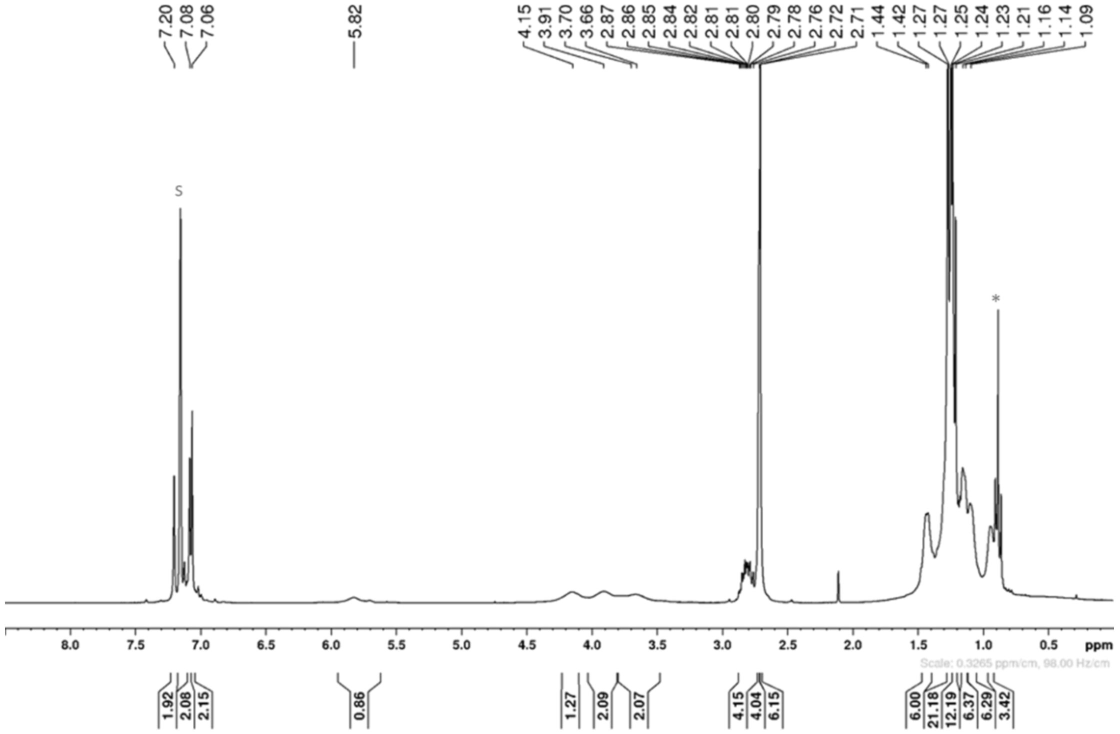

Figure S16. ^{1}H NMR of **3**-*d*$_{10}$ in C_6D_6 at 300K

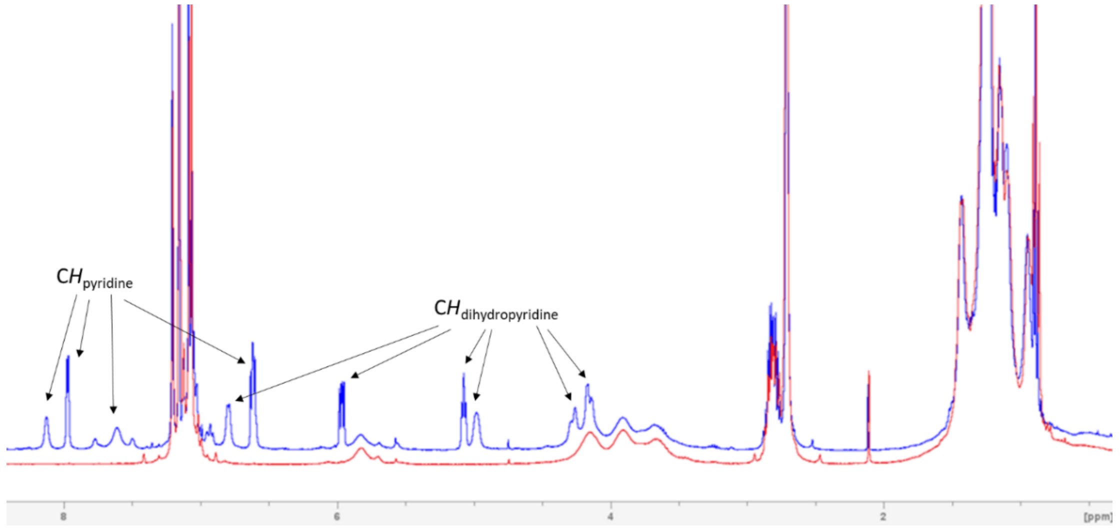

Figure S17. Stacked ^{1}H NMR spectra of **3**·[Li(dme)$_2$] (blue) and **3**-*d*$_{10}$ (red) in C_6D_6 at 300K. C*H*$_{dihydropyridine}$ and C*H*$_{pyridine}$ peaks of **3**·[Li(dme)$_2$] (blue) are not observed in **3**-*d*$_{10}$ (red).

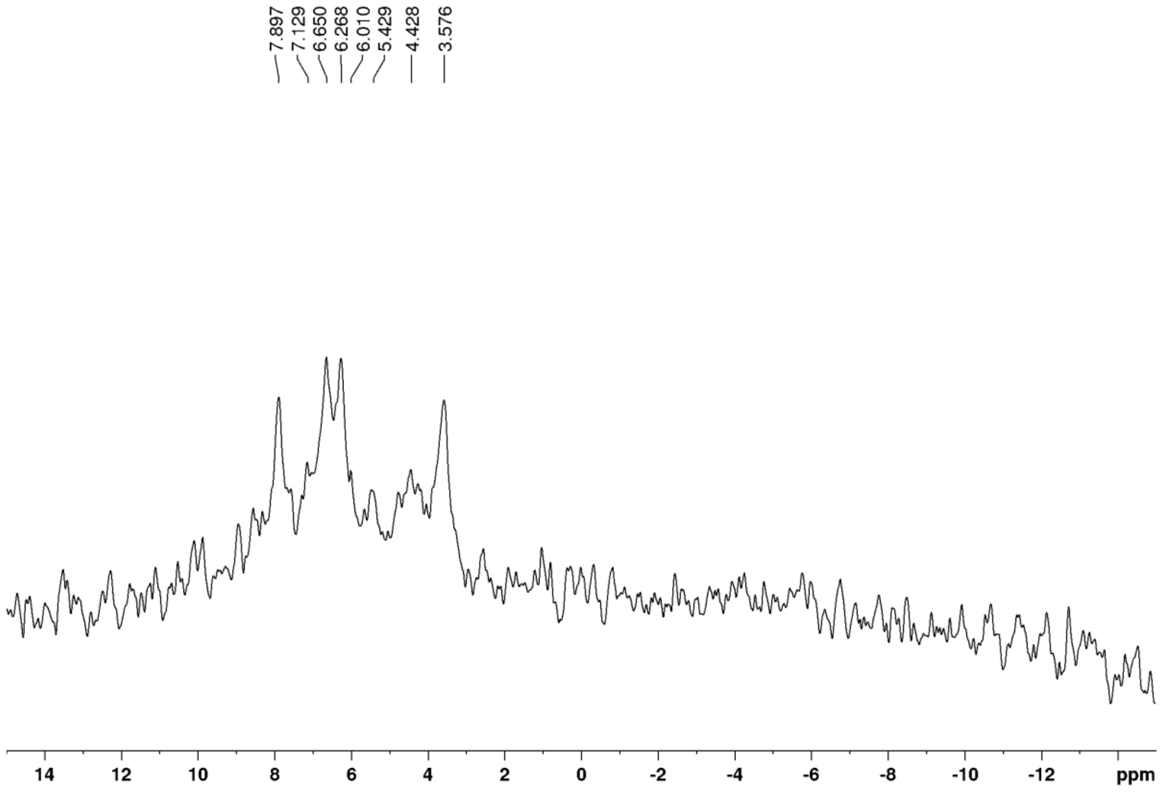

Figure S18. ^{2}H NMR spectra of **3**-d_{10} in C_6H_6 at 300K.

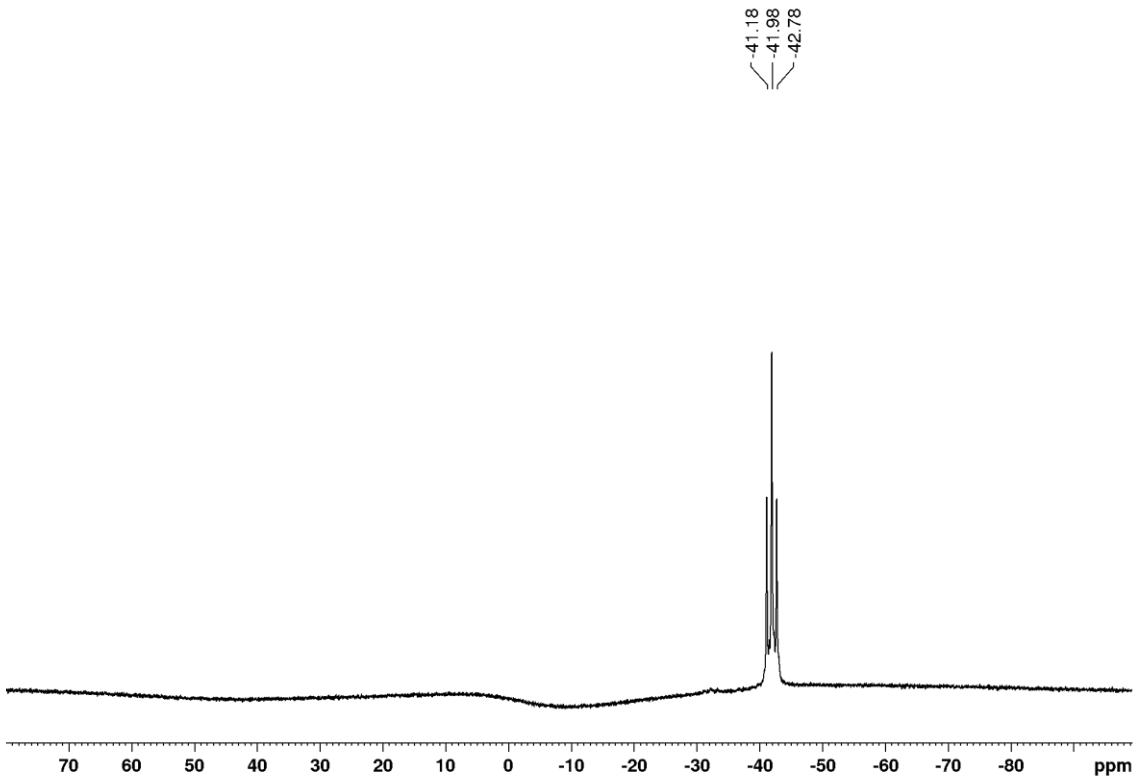

Figure S19. ^{11}B NMR of **3**-d_{10} in C_6D_6 at 300K

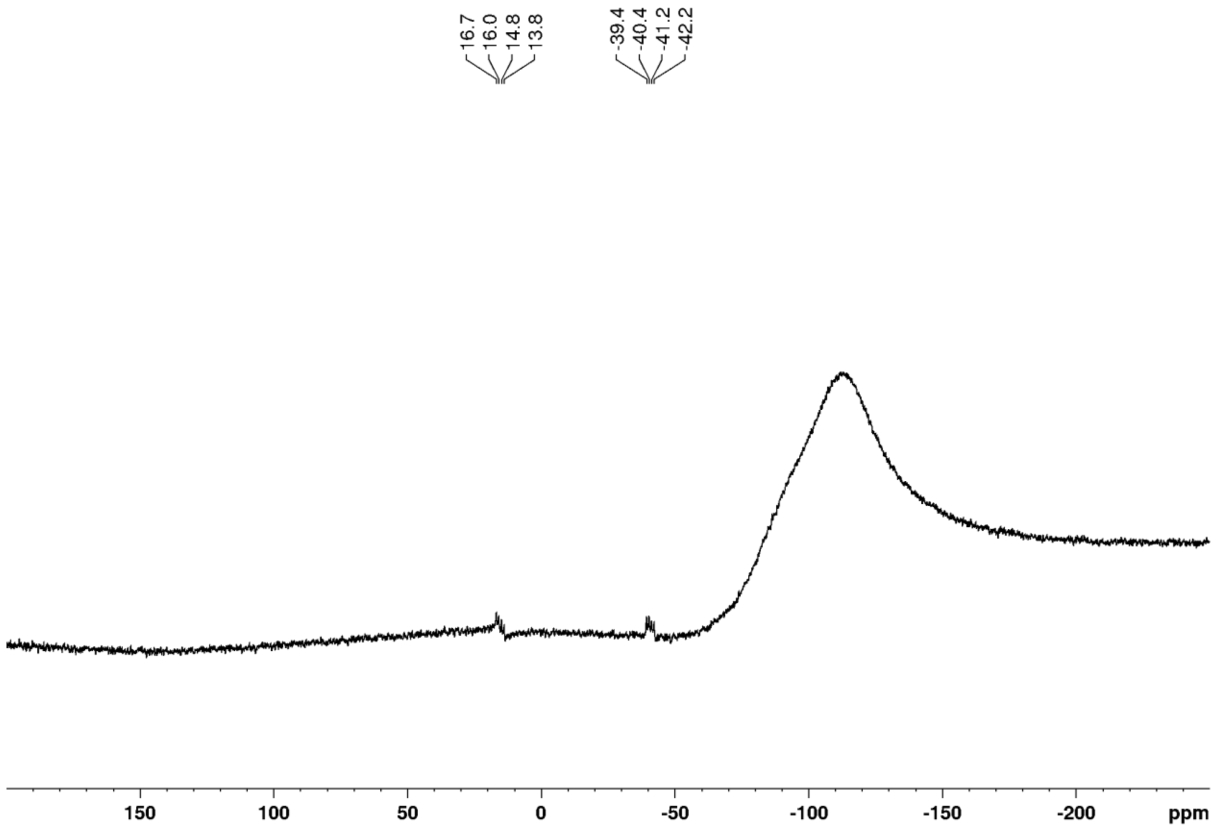

Figure S20. ^{29}Si NMR of **3**-d_{10} in C_6D_6 at 300K

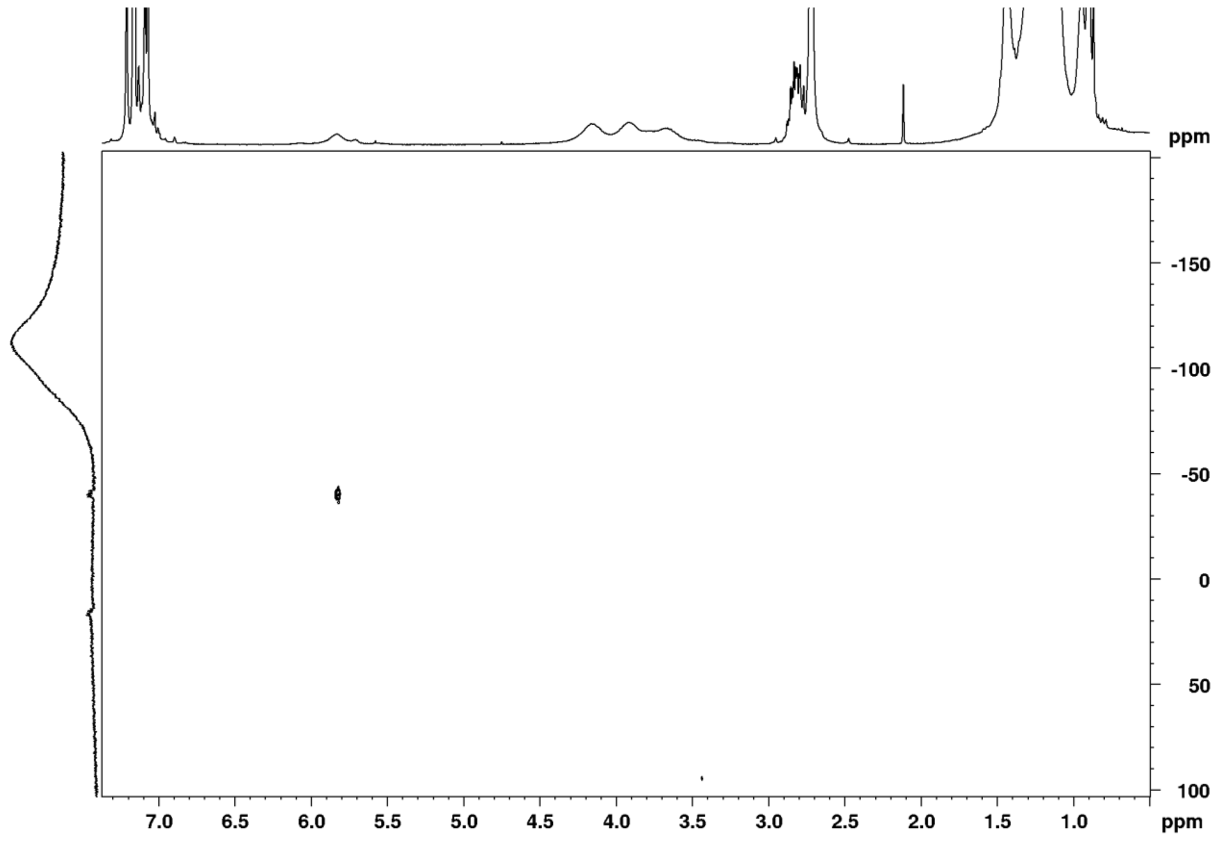

Fig S21: ^{29}Si-^{1}H HMQC NMR of **3**-d_{10} in C_6D_6 at 300K.

1.5 Synthesis of **4**·[Li(dme)$_2$]:

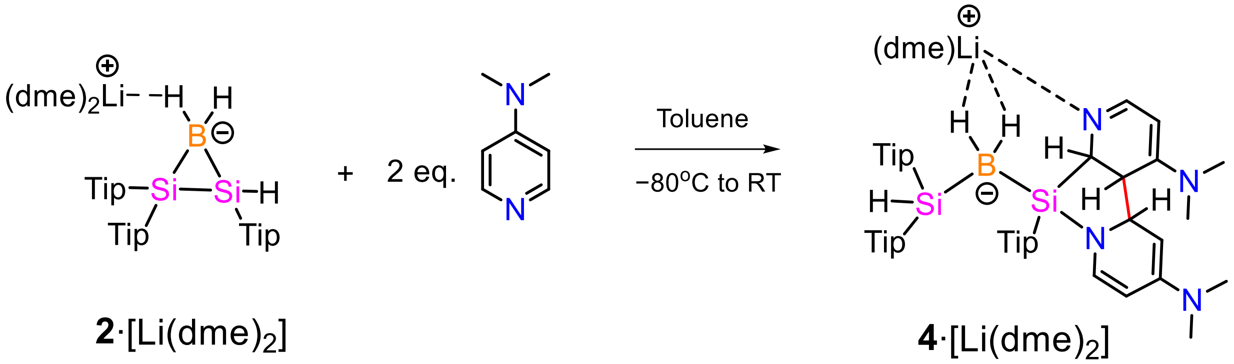

2·[Li(dme)$_2$] 867.23 g/mol | 122.17 g/mol | **4**·[Li(dme)$_2$] 1021.46 g/mol

4-Dimethylaminopyridine (122 mg, 1 mmol) and **2**·[Li(dme)$_2$] (434 mg, 0.5 mmol) are combined is a 50 mL Schlenk flask. Toluene (10 mL) is added to the solid mixture at −80 °C with vigorous stirring. The reaction mixture is stirred overnight at room temperature. After filtration, the toluene solution is concentrated to dryness and crystallized from a toluene-hexane (1:1) mixture. Colorless crystals of **4**·[Li(dme)$_2$] (310 mg, 61%) are obtained at room temperature after 24 h.

^{1}H NMR (400.13 MHz, C_6D_6, 300 K): δ = 8.02 (br s, 1H, CH_{DMAP}), 7.78 (d, 1H, $^3J_{HH}$ = 7.3 Hz, CH_{DMAP}), 7.47 (dd, 1H, $^3J_{HH}$ = 4.3 and $^4J_{HH}$ =2.9 Hz, CH_{DMAP}), 7.15 (s, 2H, CH_{Ph}, merged with C_6D_6), 7.13 (s, 2H, CH_{Ph}), 7.08 (s, 2H, CH_{Ph}), 6.15 (br s, 1H, $^1J_{SiH}$ = 188.9 Hz, Si*H*), 6.00 (d, 1H, $^3J_{HH}$ = 5.9 Hz, CH_{DMAP}), 5.30 (dd, 1H, $^3J_{HH}$ = 7.3 and $^4J_{HH}$ = 2.2 Hz, CH_{DMAP}), 4.55 to 4.57 (m, 1H, CH_{DMAP}), 4.38 to 4.32 (m, 2H, C*H*(CH_3)$_2$), 4.03-3.93 (m, 2H, C*H*(CH_3)$_2$), 3.90 (dd, 1H, $^4J_{HH}$ = 2.2 Hz, CH_{DMAP}, merged), 3.75 (dd, 1H, $^3J_{HH}$ = 5.8 and $^4J_{HH}$ = 3.0 Hz, CH_{DMAP}), 3.05 to 3.01 (m, 1H, C*H*(CH_3)$_2$), 2.92 (s, 6H, $CH_{3,DME}$, merged), 2.90 (s, 4H, $CH_{2,DME}$, merged), 2.87-2.77 (m, 4H, C*H*(CH_3)$_2$), 2.50 (s, 6H, N(CH_3)$_2$), 2.19 (s, 6H, N(CH_3)$_2$), 1.36 (d, 6H, $^3J_{HH}$ = 6.5 Hz, CH(CH_3)$_2$), 1.29 (d, 6H, $^3J_{HH}$ = 6.8 Hz, CH(CH_3)$_2$, merged), 1.28 (d, 6H, $^3J_{HH}$ = 6.8 Hz, CH(CH_3)$_2$, merged), 1.26 (d, 6H, $^3J_{HH}$ = 6.8 Hz, CH(CH_3)$_2$, merged), 1.25 (d, 9H, $^3J_{HH}$ = 6.8 Hz, CH(CH_3)$_2$, merged), 1.22 (d, 9H, $^3J_{HH}$ = 6.8 Hz, CH(CH_3)$_2$), 1.19 (d, 6H, $^3J_{HH}$ = 6.5 Hz, CH(CH_3)$_2$), 1.08 (d, 6H, $^3J_{HH}$ = 6.6 Hz, CH(CH_3)$_2$), ppm. Note: The signals for BH_2 moiety could not be identified due to broadening.

^{13}C{^{1}H} NMR (100.61 MHz, C_6D_6, 300 K): δ = 158.0, 157.8, 154.8, 154.6, 154.2 (Tip-*C*), 149.4 (*s*, CH_{DMAP}), 148.8, 147.8, 147.2, 146.8 (Tip-*C*), 142.6 (*s*, *C*H_{DMAP}), 121.5, 121.2, 120.9, 120.8, 120.7, 120.6, 120.3, 119.7 (Tip-*C*H), 106.3 (*s*, *C*H_{DMAP}), 98.1 (*s*, *C*H_{DMAP}), 87.6 (*s*, *C*H_{DMAP}), 84.7 (*s*, *C*H_{DMAP}), 70.2 (*s*, *C*$H_{2,DME}$), 63.1 (*s*, *C*H_{DMAP}), 58.5 (*s*, *C*$H_{3,DME}$), 57.6 (*s*, *C*H_{DMAP}), 40.6 (*s*, N(*C*H_3)$_3$), 40.5 (*s*, N(CH_3)$_3$), 40.1 (*s*, *C*H(CH_3)$_2$), 38.4 (*s*, N(*C*H_3)$_3$), 37.8 (*s*, N(*C*H_3)$_3$), 34.4, 34.3, 34.3, 33.1, 32.9, 32.7, 32.5, 31.5 (*s*, *C*H(CH_3)$_2$), 25.8, 25.7, 25.4, 25.1, 24.9, 24.8, 24.7, 24.1, 24.1, 24.0, 24.0, 23.9, 23.9 (*s*, CH(*C*H3)2) ppm.

^{29}Si{^{1}H} NMR (79.49 MHz, C_6D_6, 300 K): δ = −39.2 to −41.9 (m), 23.4 to 26.3 (m) ppm.

^{11}B NMR (128.38 MHz, C_6D_6, 300 K): δ = −39.1 (t, J= 78.9 Hz)

Elemental Analysis: Calcd. for $C_{63}H_{102}BSi_2LiN_4O_2$, [1021.46]: C, 74.08; H, 10.07; N, 5.49. Found: C, 73.77; H, 9.15; N, 4.03.

Mp: 130 -132 °C (decomposed).

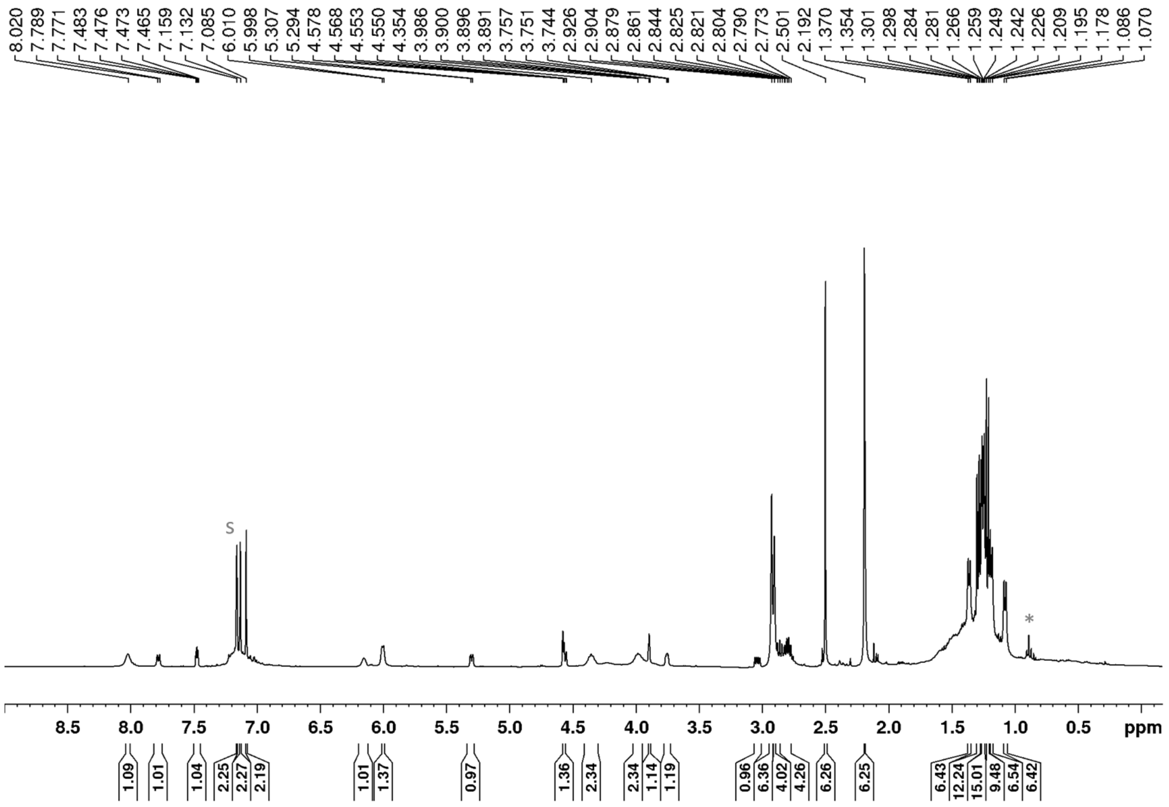

Fig S22: ^{1}H NMR of **4**·[Li(dme)$_2$] in C_6D_6 (= s) at 300K (* = hexane).

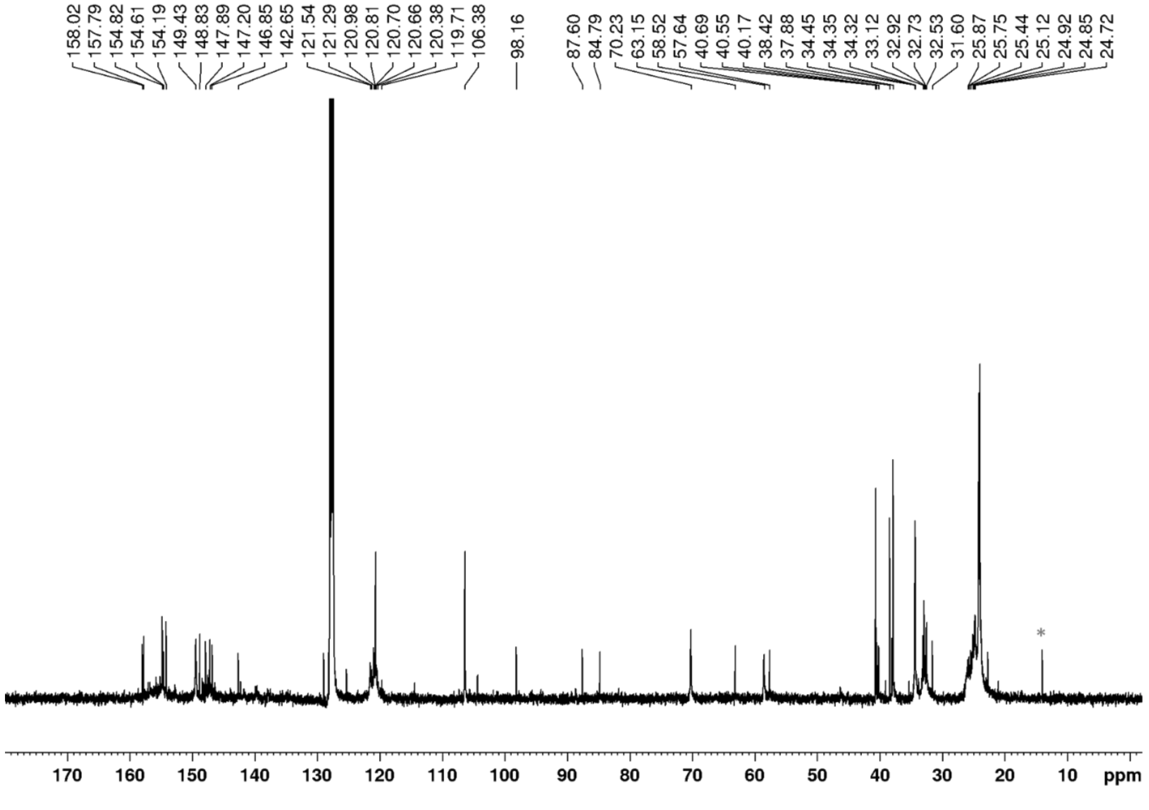

Fig S23: ^{13}C NMR of **4**·[Li(dme)$_2$] in C_6D_6 (=s) at 300K (*= hexane).

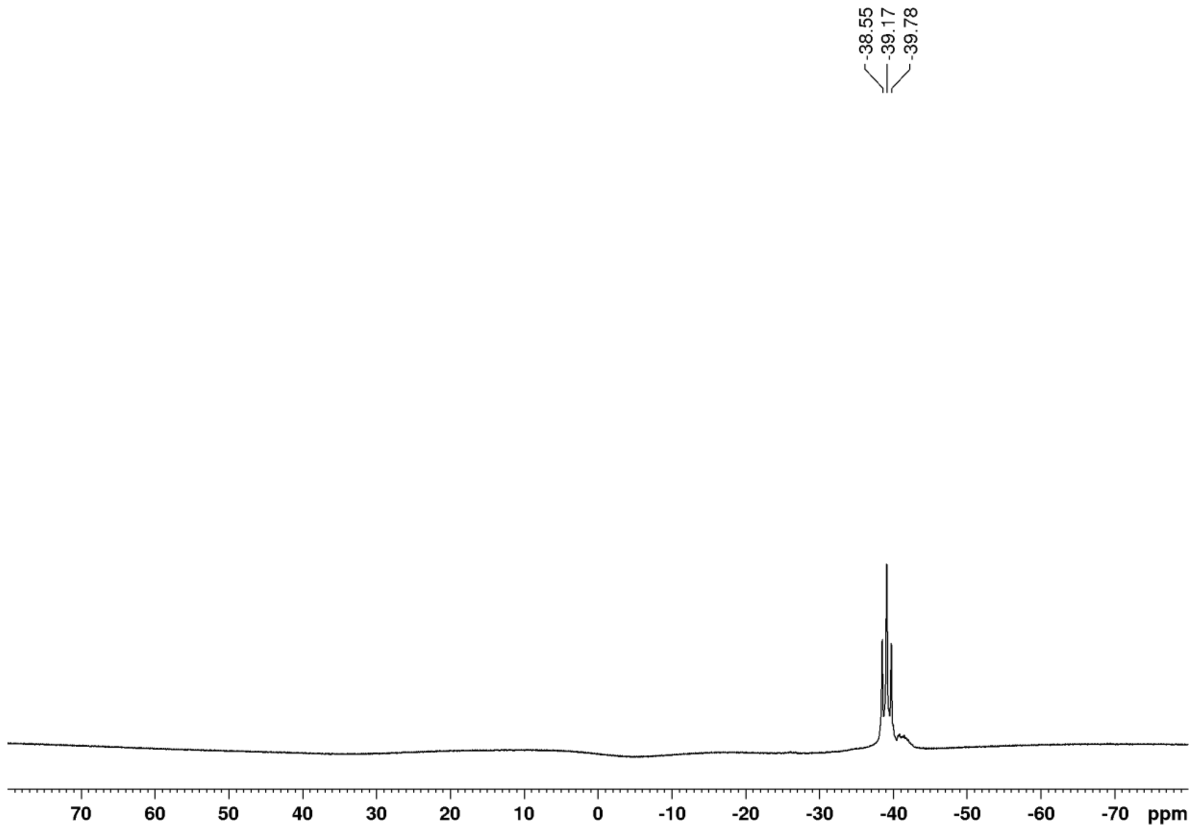

Fig S24: ^{11}B NMR of **4**·[Li(dme)$_2$] in C_6D_6 at 300K.

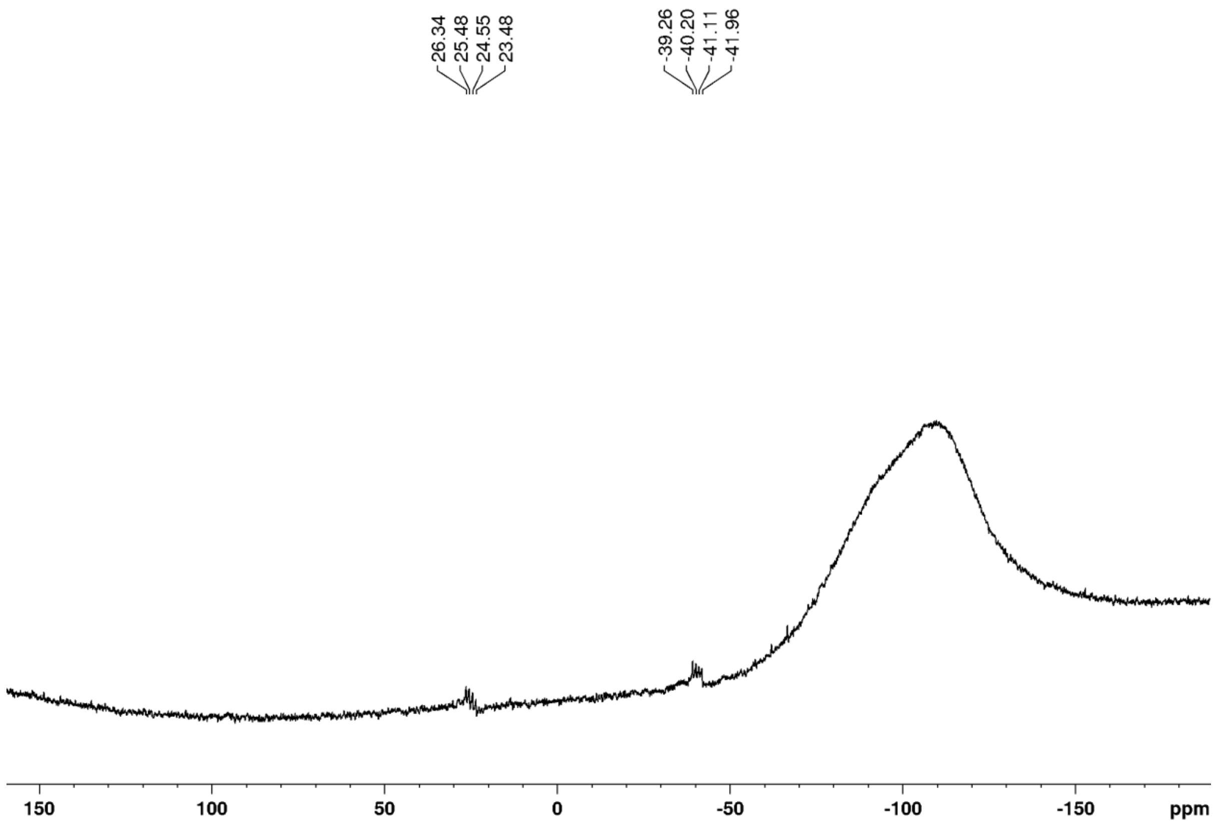

Fig S25: ^{29}Si NMR of **4**·[Li(dme)$_2$] in C_6D_6 at 300K

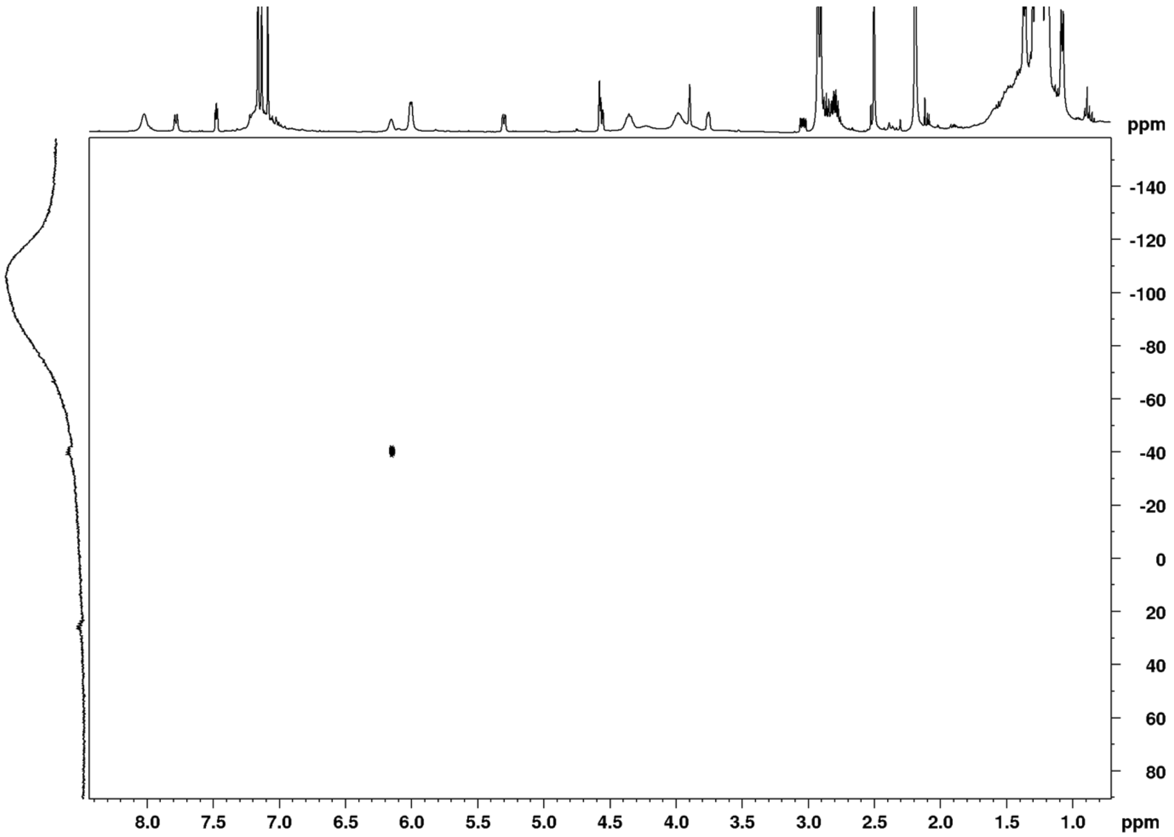

Fig S26: ^{29}Si-^{1}H HMQC NMR of **4**·[Li(dme)$_2$] in C_6D_6 at 300K.

1.6 Synthesis of 5·[Li(dme)$_2$]**:**

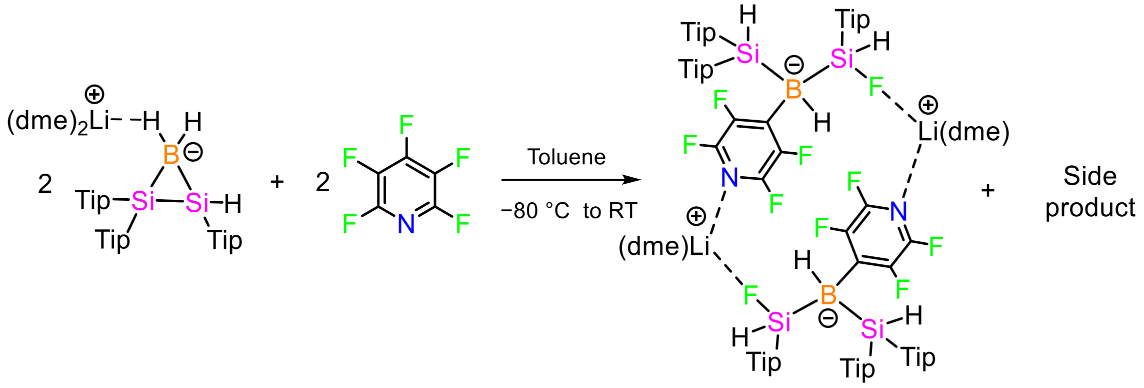

2·[Li(dme)$_2$] **5**·[Li(dme)$_2$]

867.23 g/mol 169.05 g/mol 1892.33 g/mol

Pentafluoropyridine (55 µL, 0.5 mmol) is added dropwise to the solution of **2**·[Li(dme)$_2$] (434 mg, 0.5 mmol) in toluene at −80 °C with vigorous stirring in a 50 mL Schlenk flask. The reaction mixture is stirred overnight at room temperature. After filtration, the toluene solution is concentrated to dryness and crystallized from a toluene-hexane (1:1) mixture. Colorless crystals of **5**·[Li(dme)$_2$] along with another side product are obtained at room temperature after 24 h. Several attempts to further purify **5**·[Li(dme)$_2$] from the mixture were unsuccessful.

^{11}B NMR (128.38 MHz, C_6D_6, 300 K): δ = −33.2 (d, $^1J_{B,H}$ = 83.9 Hz) and −38.8 (t, $^1J_{B,H}$ = 89.4 Hz) ppm.

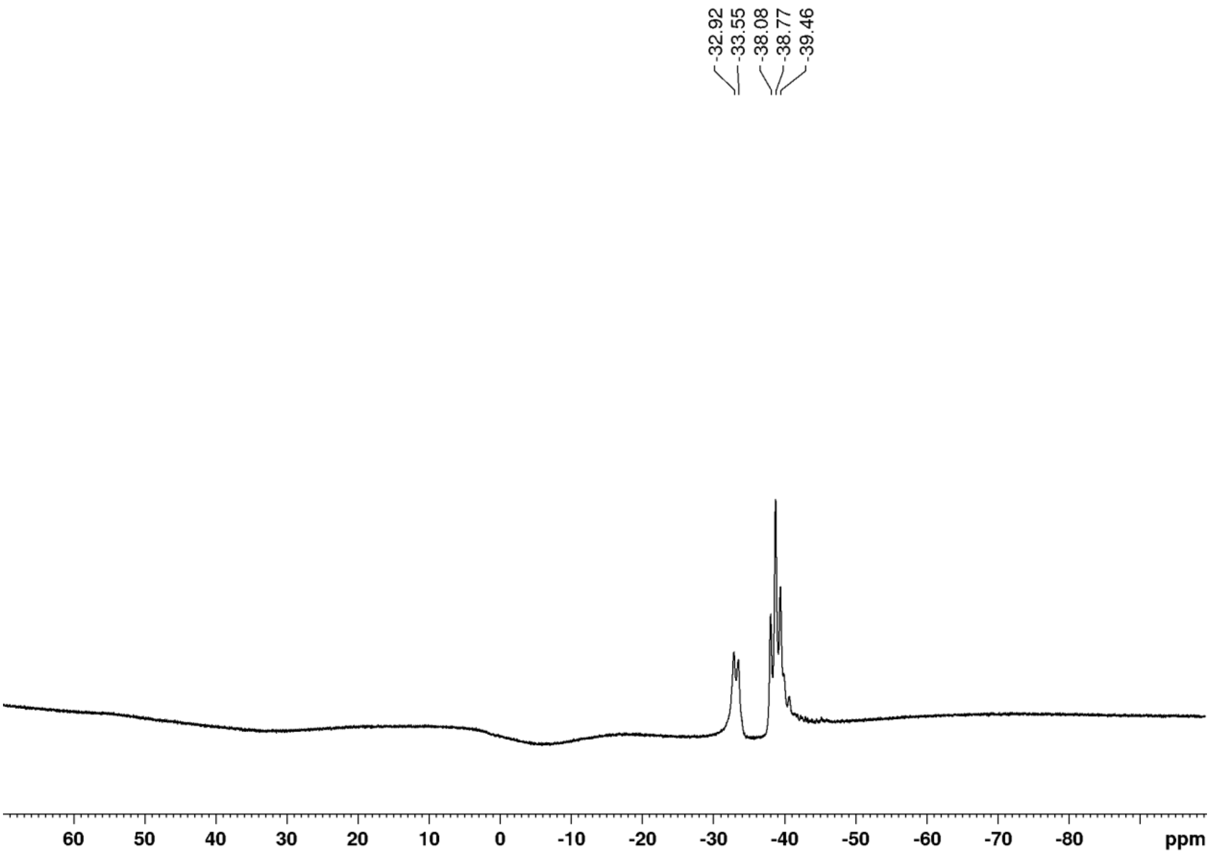

Fig S27: ^{11}B NMR of **5**·[Li(dme)$_2$] in C_6D_6 at 300K.

1.7 Synthesis of 6a·[Li(12-c-4)$_2$] and 6b·[Li(12-c-4)$_2$]:

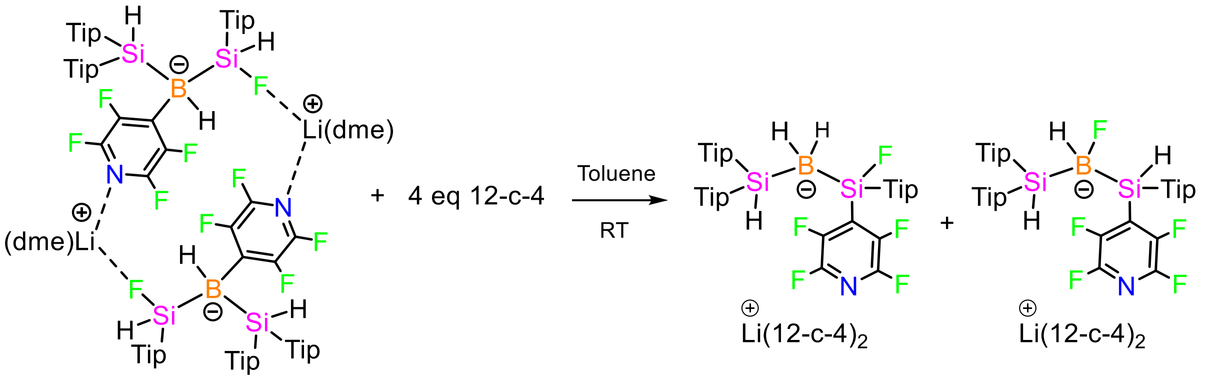

5·[Li(dme)$_2$] 1892.33 g/mol; 176.21 g/mol; **6a**·[Li(12-c-4)$_2$] 1208.47 g/mol; **6b**·[Li(12-c-4)$_2$] 1208.47 g/mol

12-c-4 (0.33 mL, 2 mmol) is added into the reaction mixture of **5**·[Li(dme)$_2$] and the side product (mentioned in section 1.6) at RT. After overnight stirring at RT, the resulting suspension is filtered, concentrated to dryness and crystallized from a toluene-hexane (1:1) mixture. Colorless crystals of **6a**·[Li(12-c-4)$_2$] and **6b**·[Li(12-c-4)$_2$] (750 mg, 62%) are obtained at room

temperature after 24 h. **6a**·[Li(12-c-4)$_2$] and **6b**·[Li(12-c-4)$_2$] crystallized together with a positional disorder of the F atom. Hence, it is difficult to separate the diastereomers.

^{1}H NMR (400.13 MHz, thf-d$_8$, 300 K): δ = 6.98 (s, 4H, CH_{Ph}), 6.85 (s, 2H, CH_{Ph}), 6.84 (s, 2H, CH_{Ph}), 6.83 (s, 2H, CH_{Ph}), 6.77 (s, 2H, CH_{Ph}), 5.20 (m, 1H, $^{1}J_{SiH}$ = 150.3 Hz, Si*H*), 5.15 (m, 1H, $^{1}J_{SiH}$ = 140.3 Hz, Si*H*), 5.09 (m, 1H, $^{1}J_{SiH}$ = 164.4 Hz, Si*H*), 4.23 to 4.08 (m, 7H, C*H*(CH$_3$)$_2$), 3.61 to 3.58 (m, 2H, C*H*(CH$_3$)$_2$), 3.67 (s, 64H, C$H_{2,12\text{-}c\text{-}4}$), 3.53 to 3.47 (m, 2H, C*H*(CH$_3$)$_2$), 2.89 to 2.72 (m, 7H, C*H*(CH$_3$)$_2$), 1.28 (d, 12H, $^{3}J_{HH}$ = 6.9 Hz, CH(CH_3)$_2$), 1.19, 1.20,1.21, 1.23 (d, 24H, $^{3}J_{HH}$ = 6.8 Hz, CH(CH_3)$_2$), 1.16, 1.17 (d, 18H, $^{3}J_{HH}$ = 6.7 Hz, CH(CH_3)$_2$, merged), 1.09 (d, 6H, $^{3}J_{HH}$ = 6.7 Hz, CH(CH_3)$_2$, merged), 1.03, 1.03, 1.04, 1.05 (d, 24H, $^{3}J_{HH}$ = 6.3 Hz, CH(CH_3)$_2$, merged), 0.87 (d, 6H, $^{3}J_{HH}$ = 6.5 Hz, CH(CH_3)$_2$), 0.83 (d, 6H, $^{3}J_{HH}$ = 6.5 Hz, CH(CH_3)$_2$), 0.75 (d, 6H, $^{3}J_{HH}$ = 6.6 Hz, CH(CH_3)$_2$), 0.72 (d, 6H, $^{3}J_{HH}$ = 6.6 Hz, CH(CH_3)$_2$), ppm. Note: The signals for BH$_2$ moiety could not be identified due to broadening.

^{13}C{^{1}H} NMR (100.61 MHz, thf-d$_8$, 300 K): δ = 157.5, 157.2, 156.8, 156.1, 155.0, 149.8, 149.6, 148.4, 147.8, 147.3 (Tip-*C*), 146.0 (m, *C*F$_{py}$), 145.4 (m, *C*F$_{py}$), 144.6 (Tip-*C*), 143.5 (m, *C*F$_{py}$), 143.0 (m, *C*F$_{py}$), 141.5, 141.4, 140.3, 140.2, 137.0, 136.9, 134.1 (Tip-*C*), 121.9, 121.7, 121.5, 121.2, 121.2 (Tip-*C*H), 69.8 (*s*, *C*H$_{2,12\text{-}c\text{-}4}$), 35.8, 35.8, 34.7, 34.6, 34.1, 33.4, 33.1, 33.0, 32.9 (*s*, *C*H(CH$_3$)$_2$), 26.5, 26.4, 25.8, 25.7, 25.3, 25.2, 25.2, 25.1, 25.0, 25.0 (*s*, CH(*C*H3)$_2$) ppm.

^{11}B NMR (128.38 MHz, thf-d$_8$, 300 K): δ = −32.7 (br d, J= 81.5 Hz) (**6b**·[Li(12-c-4)$_2$]) and −37.8 (t, J= 86.9 Hz) (**6a**·[Li(12-c-4)$_2$]) ppm.

^{29}Si{^{1}H} NMR (79.49 MHz, thf-d$_8$, 300 K): δ = 30.9 (br d, *Si*-F), −39.8 (br m, *Si*-H), −47.2 (m, *Si*-H) and −48.8 (m, *Si*-H) ppm.

^{19}F NMR (282.23 MHz, thf-d$_8$, 300 K): δ = −98.8 (m, 4F, Py-*F*), −127.0 (m, 2F, Py-*F*), −131.1 (m, 2F, Py-*F*) and −154.7 (br s, 1F, $^{1}J_{Si\text{-}F}$ = 339.4 Hz, Si-*F*) ppm. The signals for BF could not be identified due to broadening.

^{7}Li NMR (155.50 MHz, thf-d$_8$, 300K): δ = −0.65 (*s*) ppm.

Elemental Analysis: Calcd. for $C_{132}H_{208}B_2Si_4Li_2N_2O_{16}F_{10}$, [2416.94]: C, 65.60; H, 8.67; N, 1.16. Found: C, 65.34; H, 7.66; N, 0.81.

Mp: 140 - 143 °C (decomposed).

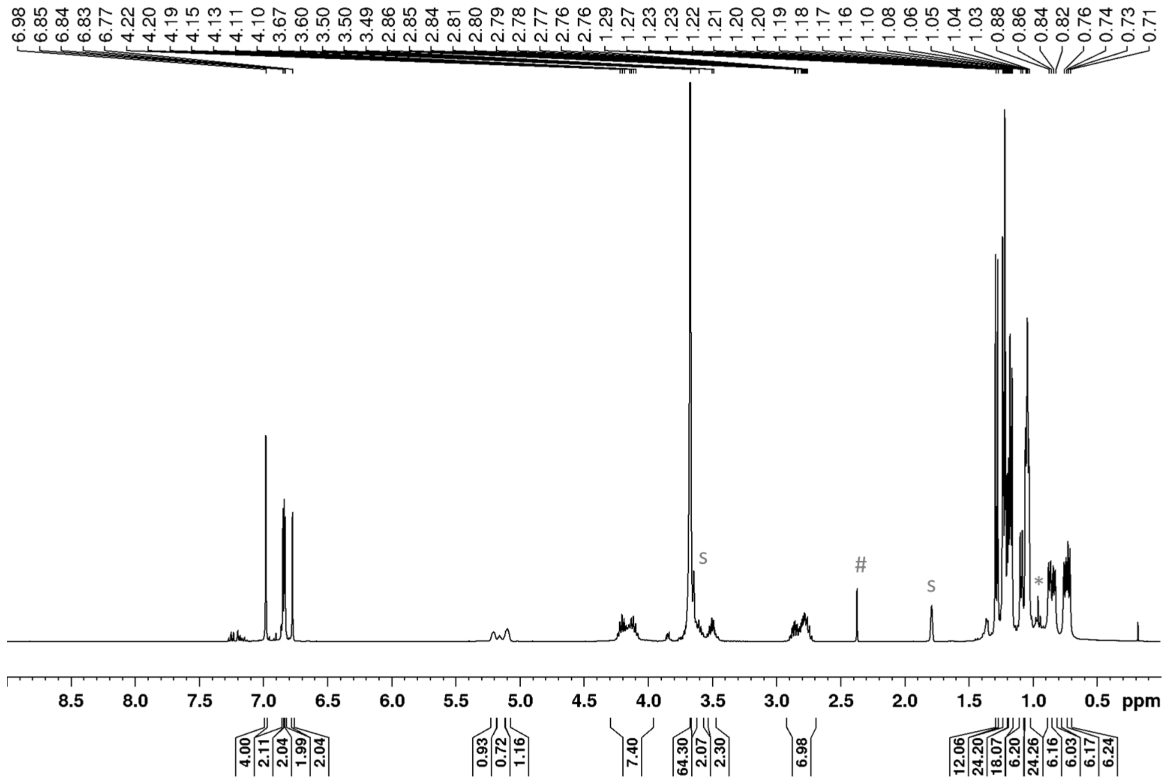

Fig S28: ^{1}H NMR of **6a**·[Li(12-c-4)$_2$] and **6b**·[Li(12-c-4)$_2$] in thf-d$_8$ (= s) at 300K (* = hexane, # = toluene).

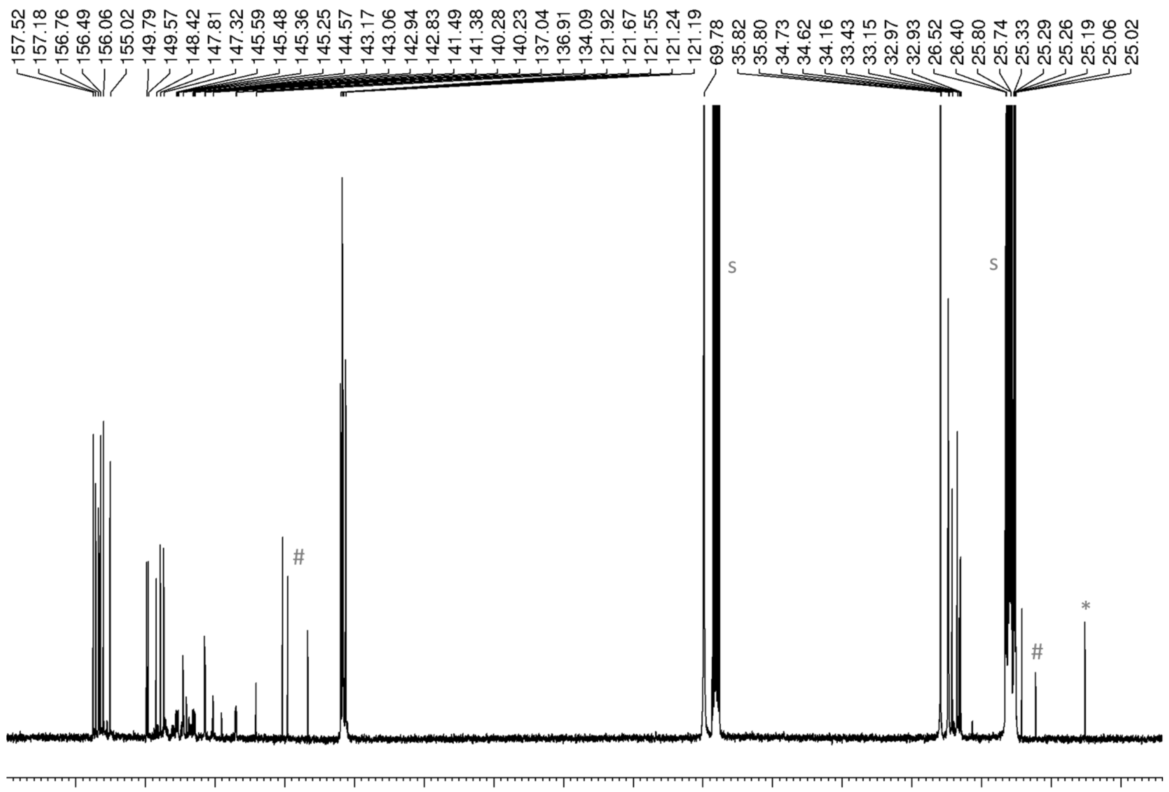

Fig S29: ^{13}C NMR of **6a**·[Li(12-c-4)$_2$] and **6b**·[Li(12-c-4)$_2$] in thf-d$_8$ (= s) at 300K (* = hexane, # = toluene).

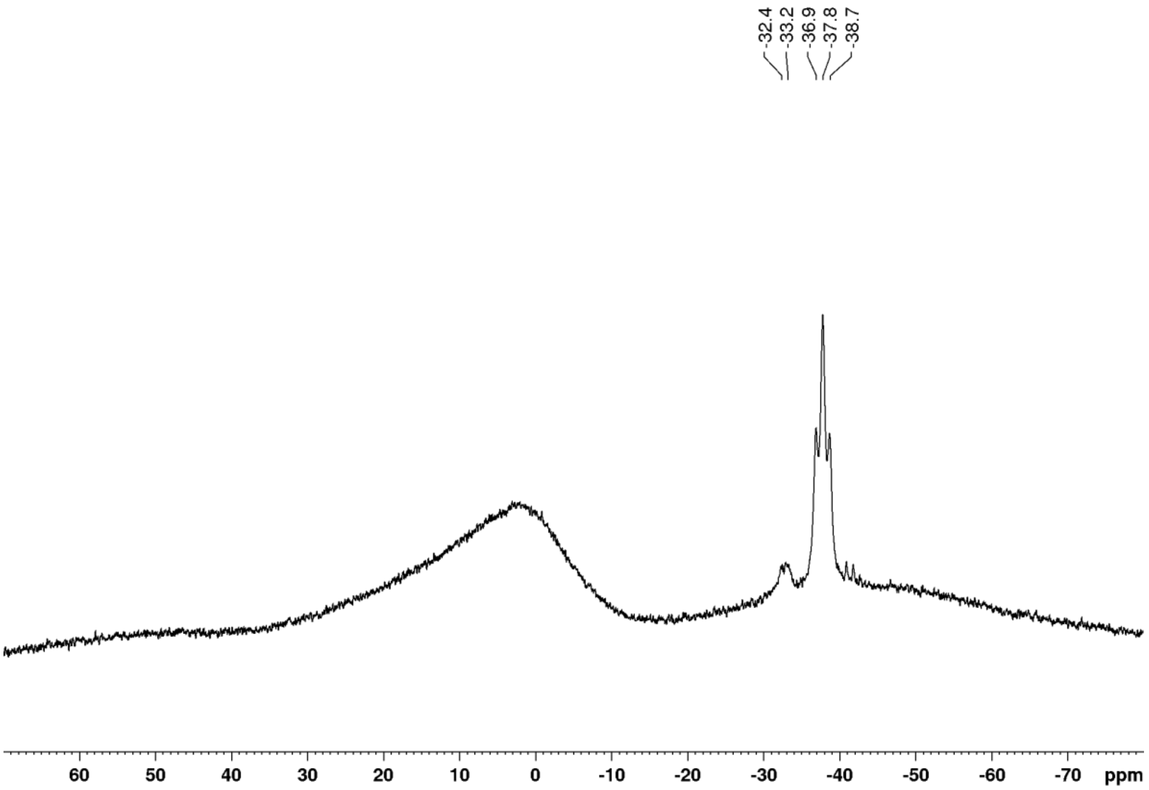

Fig S30: ^{11}B NMR of **6a**·[Li(12-c-4)$_2$] and **6b**·[Li(12-c-4)$_2$] in thf-d$_8$ at 300K.

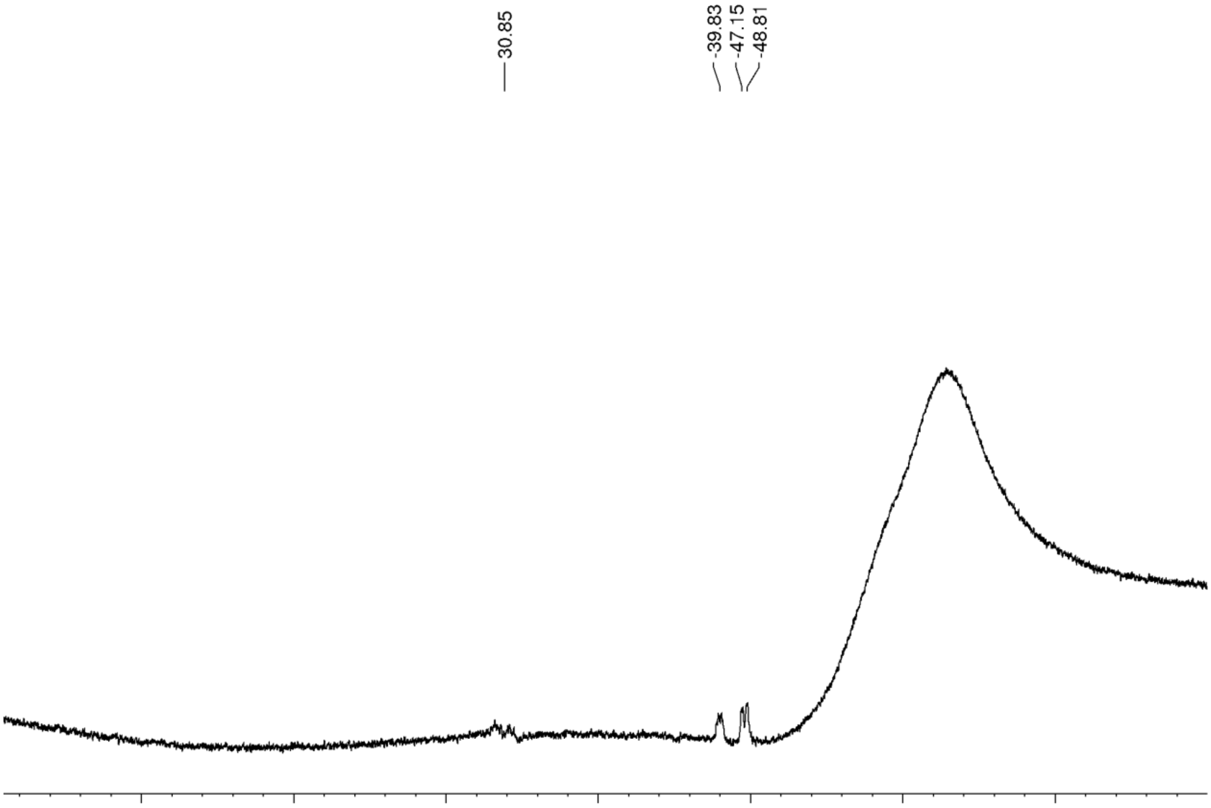

Fig S31: ^{29}Si NMR of **6a**·[Li(12-c-4)$_2$]and **6b**·[Li(12-c-4)$_2$] in thf-d$_8$ at 300K.

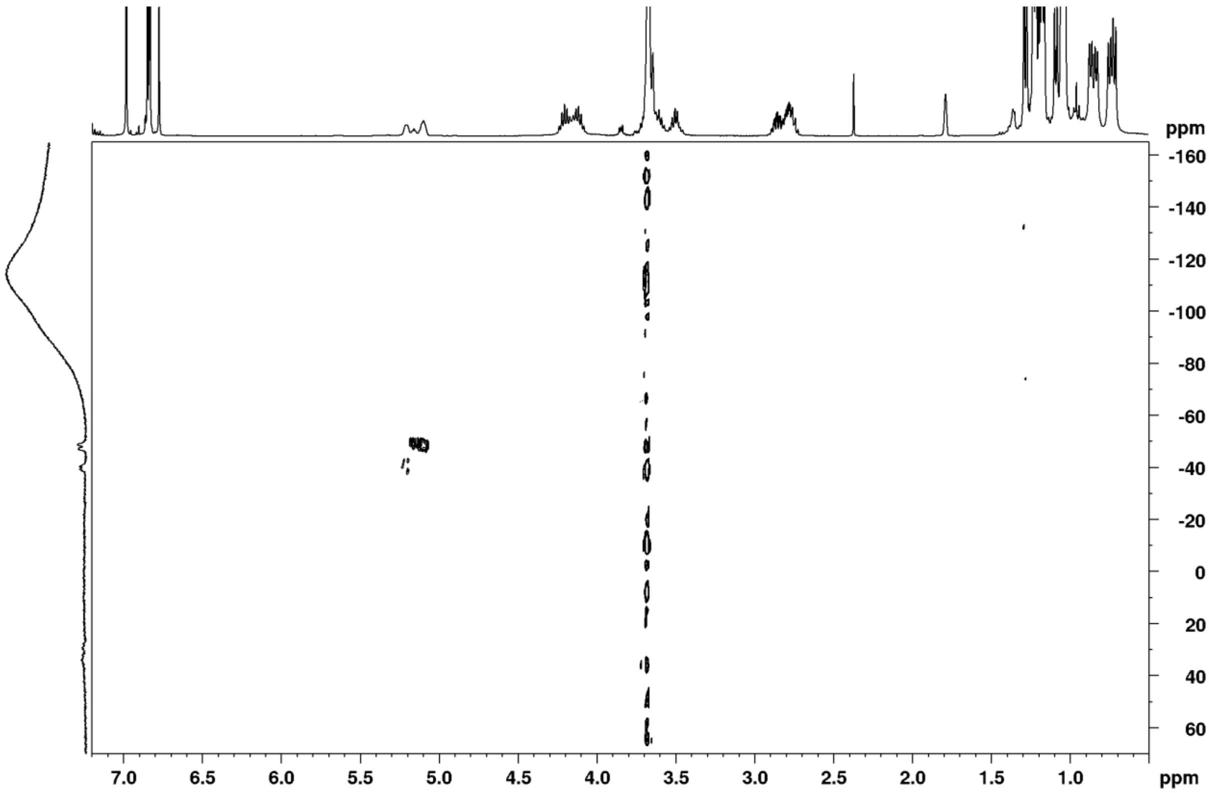

Fig S32: ^{29}Si-^{1}H HMQC NMR of **6a**·[Li(12-c-4)$_2$] and **6b**·[Li(12-c-4)$_2$] in thf-d_8 at 300K.

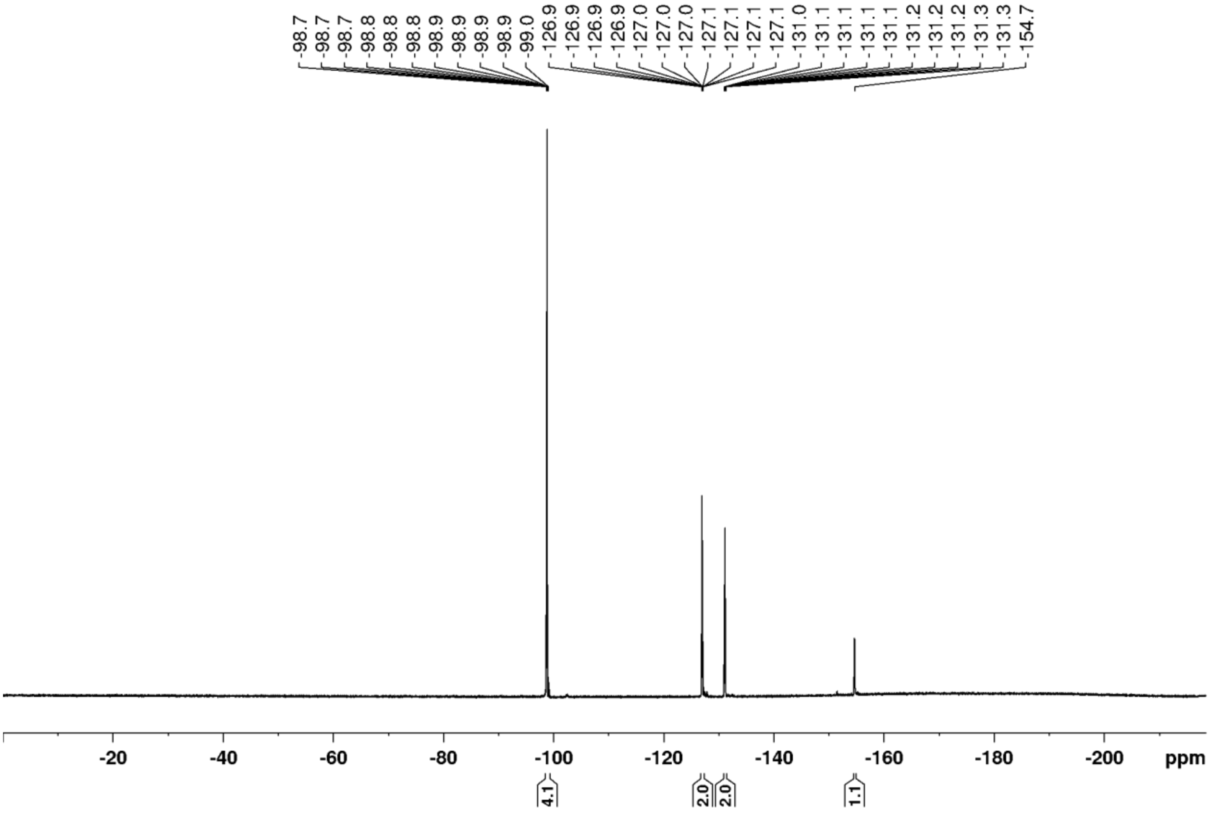

Fig S33: ^{19}F NMR of **6a**·[Li(12-c-4)$_2$] and **6b**·[Li(12-c-4)$_2$] in thf-d_8 at 300K.

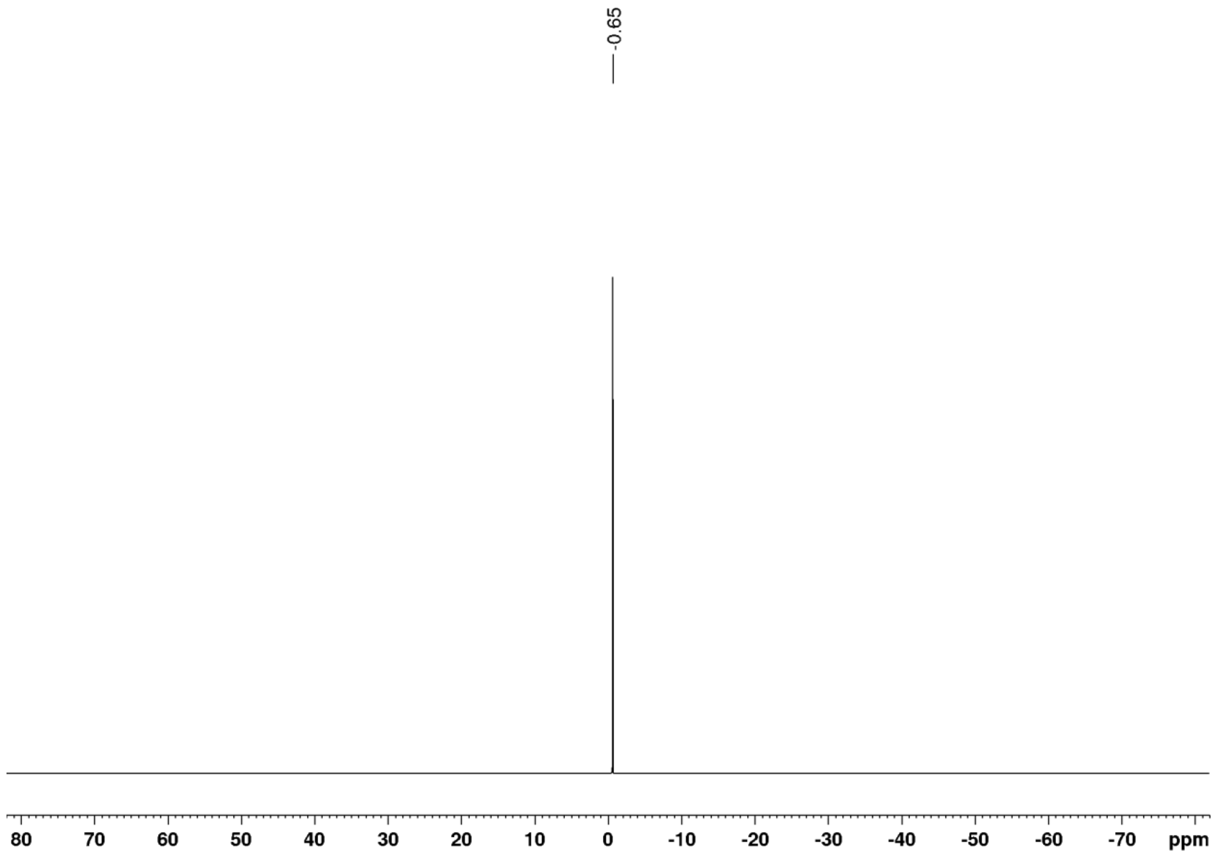

Fig S34: ^{7}Li NMR of **6a**·[Li(12-c-4)$_2$] and **6b**·[Li(12-c-4)$_2$] in thf-d$_8$ at 300K.

2. Crystallographic data

The data sets were acquired using a Bruker D8 Venture diffractometer (**2**·[Li(dme)$_2$], **3**·[Li(dme)$_2$], **4**·[Li(dme)$_2$]) with a microfocus sealed tube and a Photon II detector. Monochromated Mo$_{K\alpha}$ radiation (λ = 0.71073 Å) was used. Data were corrected for absorption effects using the multi-scan method. The structures were solved by direct methods using SHELXT[S3] and were refined by full matrix least squares calculations on F^2 (SHELXL2018[S4]) in the graphical user interface Shelxle[S5].

Acknowledgment: Instrumentation and technical assistance for this work were provided by the Service Center X-ray Diffraction, with financial support from Saarland University and German Science Foundation (project number INST 256/506-1).

2.1. Crystal structure of 2·[Li(dme)$_2$]

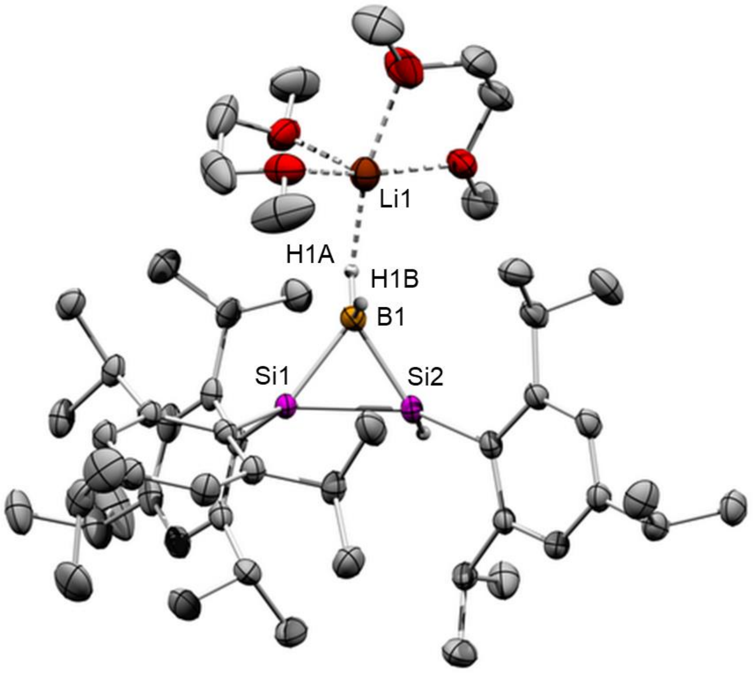

Refinement: All non H-atoms were located in the electron density maps and refined anisotropically. C-bound H atoms were placed in positions of optimized geometry and treated as riding atoms. Their isotropic displacement parameters were coupled to the corresponding carrier atoms by a factor of 1.2 (CH, CH_2) or 1.5 (CH_3). The B1 and Si2 bonded H atoms were located in the electron density maps and their positional parameters were refined using isotropic displacement parameters which were set at 1.2 times the Ueq value of their parent atoms.

Table S1. Crystal data and structure refinement for sh5035_a.

Identification code	sh5035_a
Empirical formula	$C_{53}H_{92}BLiO_4Si_2$
Formula weight	867.19
Temperature	153(2) K
Wavelength	0.71073 Å

Crystal system	Triclinic	
Space group	*P*-1	
Unit cell dimensions	*a* = 12.8941(3) Å	α = 88.9780(10)°.
	b = 13.7042(3) Å	β = 77.4200(10)°.
	c = 15.9370(4) Å	γ = 85.0640(10)°.
Volume	2738.32(11) $Å^3$	
Z	2	
Density (calculated)	1.052 Mg/m^3	
Absorption coefficient	0.104 mm^{-1}	
F(000)	956	
Crystal size	0.220 x 0.200 x 0.100 mm^3	
Theta range for data collection	1.984 to 27.911°.	
Index ranges	−16<=h<=16, −18<=k<=18, −20<=l<=20	
Reflections collected	107188	
Independent reflections	13050 [R(int) = 0.0547]	
Completeness to theta = 25.242°	100.0 %	
Absorption correction	Semi-empirical from equivalents	
Max. and min. transmission	0.7449 and 0.7258	
Refinement method	Full-matrix least-squares on F^2	
Data / restraints / parameters	13050 / 0 / 581	
Goodness-of-fit on F^2	1.023	
Final R indices [I>2sigma(I)]	R1 = 0.0475, wR2 = 0.1133	
R indices (all data)	R1 = 0.0622, wR2 = 0.1241	
Extinction coefficient	n/a	
Largest diff. peak and hole	0.846 and −0.379 e.$Å^{-3}$	

2.2. Crystal structure of 3·[Li(dme)$_2$]

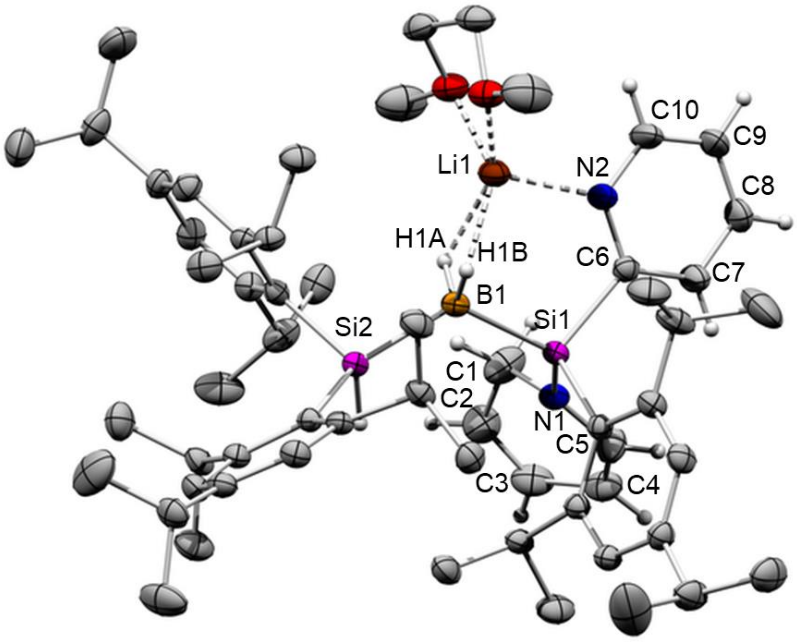

Refinement: All non H-atoms were located in the electron density maps and refined anisotropically. C-bound H atoms were placed in positions of optimized geometry and treated as riding atoms. Their isotropic displacement parameters were coupled to the corresponding carrier atoms by a factor of 1.2 (CH, CH_2) or 1.5 (CH_3). Both boron bonded H atoms and the Si2 bonded H2A were located in the electron density maps and their positional parameters were refined using isotropic displacement parameters which were set to 1.2 times the Ueq value of their parent atoms, respectively.

Table S2. Crystal data and structure refinement for sh5431_a.

Identification code	sh5431_a	
Empirical formula	$C_{59}H_{92}BLiN_2O_2Si_2$	
Formula weight	935.27	
Temperature	143(2) K	
Wavelength	0.71073 Å	
Crystal system	Monoclinic	
Space group	$P2_1/n$	
Unit cell dimensions	a = 14.9655(5) Å	α = 90°.
	b = 11.6763(4) Å	β = 97.4170(10)°
	c = 33.7137(11) Å	γ = 90°.
Volume	5841.9(3) Å^3	
Z	4	
Density (calculated)	1.063 Mg/m^3	
Absorption coefficient	0.101 mm^{-1}	
F(000)	2048	

Crystal size	0.400 x 0.200 x 0.100 mm^3
Theta range for data collection	2.128 to 27.135°.
Index ranges	−19<=h<=19, −14<=k<=14, −43<=l<=41
Reflections collected	88381
Independent reflections	12891 [R(int) = 0.0867]
Completeness to theta = 25.242°	99.9 %
Absorption correction	Semi-empirical from equivalents
Max. and min. transmission	0.7455 and 0.6728
Refinement method	Full-matrix least-squares on F^2
Data / restraints / parameters	12891 / 0 / 633
Goodness-of-fit on F^2	1.022
Final R indices [I>2sigma(I)]	R1 = 0.0495, wR2 = 0.1117
R indices (all data)	R1 = 0.0769, wR2 = 0.1290
Extinction coefficient	n/a
Largest diff. peak and hole	0.548 and −0.358 e. $Å^{-3}$

2.3. Crystal structure of 4·[Li(dme)$_2$]

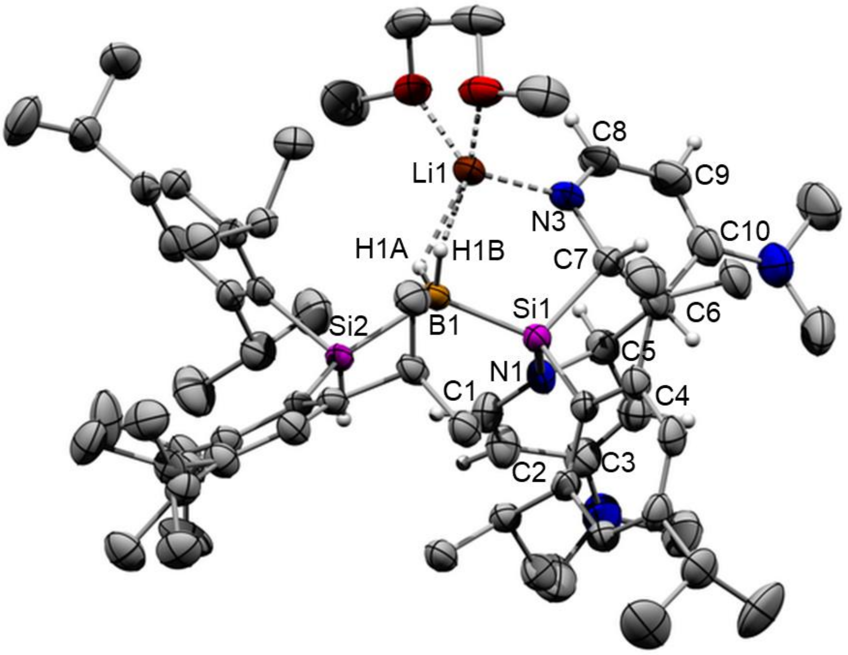

Refinement: All non H-atoms were located in the electron density maps and refined anisotropically. C-bound H atoms were placed in positions of optimized geometry and treated as riding atoms. Their isotropic displacement parameters were coupled to the corresponding carrier atoms by a factor of 1.2 (CH, CH_2) or 1.5 (CH_3). The B1 bonded H-atoms H1A and H1B were located in the electron density maps. Their positional parameters were refined using isotropic displacement parameters which were set at 1.2 times the Ueq value of B1. *Disorder:* One isopropyl-group and the n-hexane solvent molecule are split over two positions. Their occupancy factors refined to 0.75 and 0.55, respectively for the major component.

Table S3. Crystal data and structure refinement for sh5247_a.

Identification code	sh5247_a	
Empirical formula	$C_{69}H_{116}BLiN_4O_2Si_2$	
Formula weight	1107.58	
Temperature	143(2) K	
Wavelength	0.71073 Å	
Crystal system	Triclinic	
Space group	*P*-1	
Unit cell dimensions	*a* = 11.5846(5) Å	α = 77.111(2)°.
	b = 14.2042(6) Å	β = 76.532(2)°.
	c = 22.4962(10) Å	γ = 88.285(2)°.
Volume	3508.3(3) Å^3	
Z	2	
Density (calculated)	1.048 Mg/m^3	
Absorption coefficient	0.093 mm^{-1}	
F(000)	1220	

Crystal size	0.400 x 0.200 x 0.040 mm^3
Theta range for data collection	1.924 to 25.026°.
Index ranges	−13<=h<=13, −15<=k<=16, −26<=l<=26
Reflections collected	70189
Independent reflections	12381 [R(int) = 0.0718]
Completeness to theta = 25.026°	99.9 %
Absorption correction	Semi-empirical from equivalents
Max. and min. transmission	0.7458 and 0.6828
Refinement method	Full-matrix least-squares on F^2
Data / restraints / parameters	12381 / 268 / 830
Goodness-of-fit on F^2	1.020
Final R indices [I>2sigma(I)]	R1 = 0.0598, wR2 = 0.1473
R indices (all data)	R1 = 0.0901, wR2 = 0.1678
Extinction coefficient	n/a
Largest diff. peak and hole	0.352 and −0.313 e. $Å^{-3}$

2.4. Crystal structure of 5·[Li(dme)$_2$]

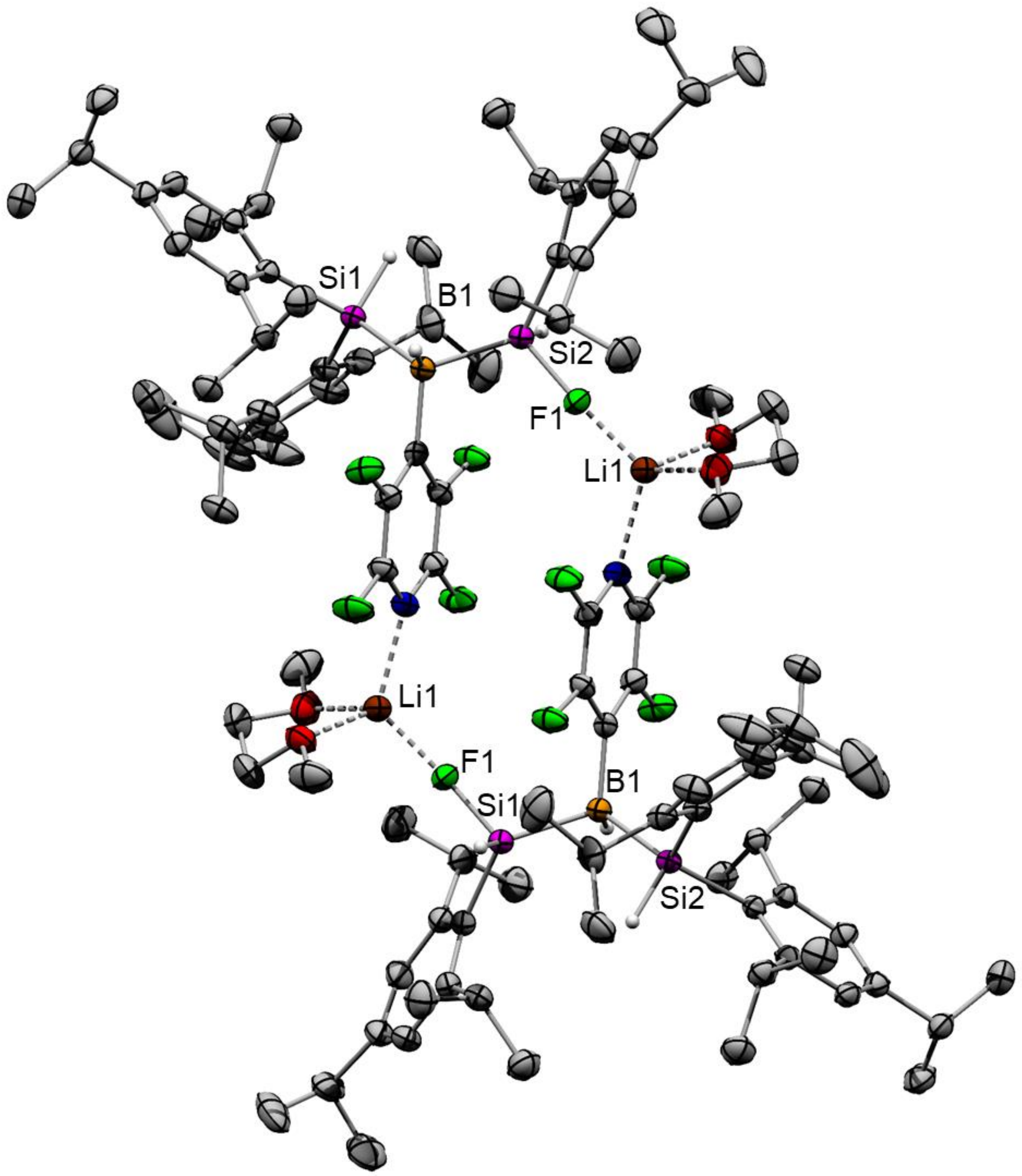

Refinement: All non H-atoms were located in the electron density maps and refined anisotropically. C-bound H atoms were placed in positions of optimized geometry and treated as riding atoms. Their isotropic displacement parameters were coupled to the corresponding carrier atoms by a factor of 1.2 (CH, CH_2) or 1.5 (CH_3). The Si1, Si2 and B1 bonded H-atoms were located in the electron density maps. Their positional parameters were refined using isotropic displacement parameters which were set at 1.2 times the Ueq value of their corresponding parent atoms. Disorder: One isopropyl-group is split over two positions. Its occupancy factors refined to 83 % for the major component.

Table S4. Crystal data and structure refinement for sh5423_a.

Identification code	sh5423_a
Empirical formula	$C_{108}H_{164}B_2F_{10}Li_2N_2O_4Si_4$
Formula weight	1892.26

Temperature	143(2) K	
Wavelength	0.71073 Å	
Crystal system	Monoclinic	
Space group	$P2_1/n$	
Unit cell dimensions	a = 18.5937(4) Å	α = 90°.
	b = 17.0002(3) Å	β = 103.2050(10)°.
	c = 18.7136(3) Å	γ = 90°.
Volume	5758.90(19) $Å^3$	
Z	2	
Density (calculated)	1.091 Mg/m^3	
Absorption coefficient	0.114 mm^{-1}	
F(000)	2040	
Crystal size	0.200 x 0.180 x 0.120 mm^3	
Theta range for data collection	2.127 to 27.891°.	
Index ranges	−24<=h<=24, −22<=k<=22, −24<=l<=24	
Reflections collected	111621	
Independent reflections	13766 [R(int) = 0.0642]	
Completeness to theta = 25.242°	99.9 %	
Absorption correction	Semi-empirical from equivalents	
Max. and min. transmission	0.7456 and 0.7102	
Refinement method	Full-matrix least-squares on F^2	
Data / restraints / parameters	13766 / 76 / 654	
Goodness-of-fit on F2	1.027	
Final R indices [I>2sigma(I)]	R1 = 0.0434, wR2 = 0.0956	
R indices (all data)	R1 = 0.0645, wR2 = 0.1082	
Extinction coefficient	n/a	
Largest diff. peak and hole	0.311 and -0.290 e.$Å^{-3}$	

2.5. Crystal structure of 6·[Li(12-c-4)$_2$]

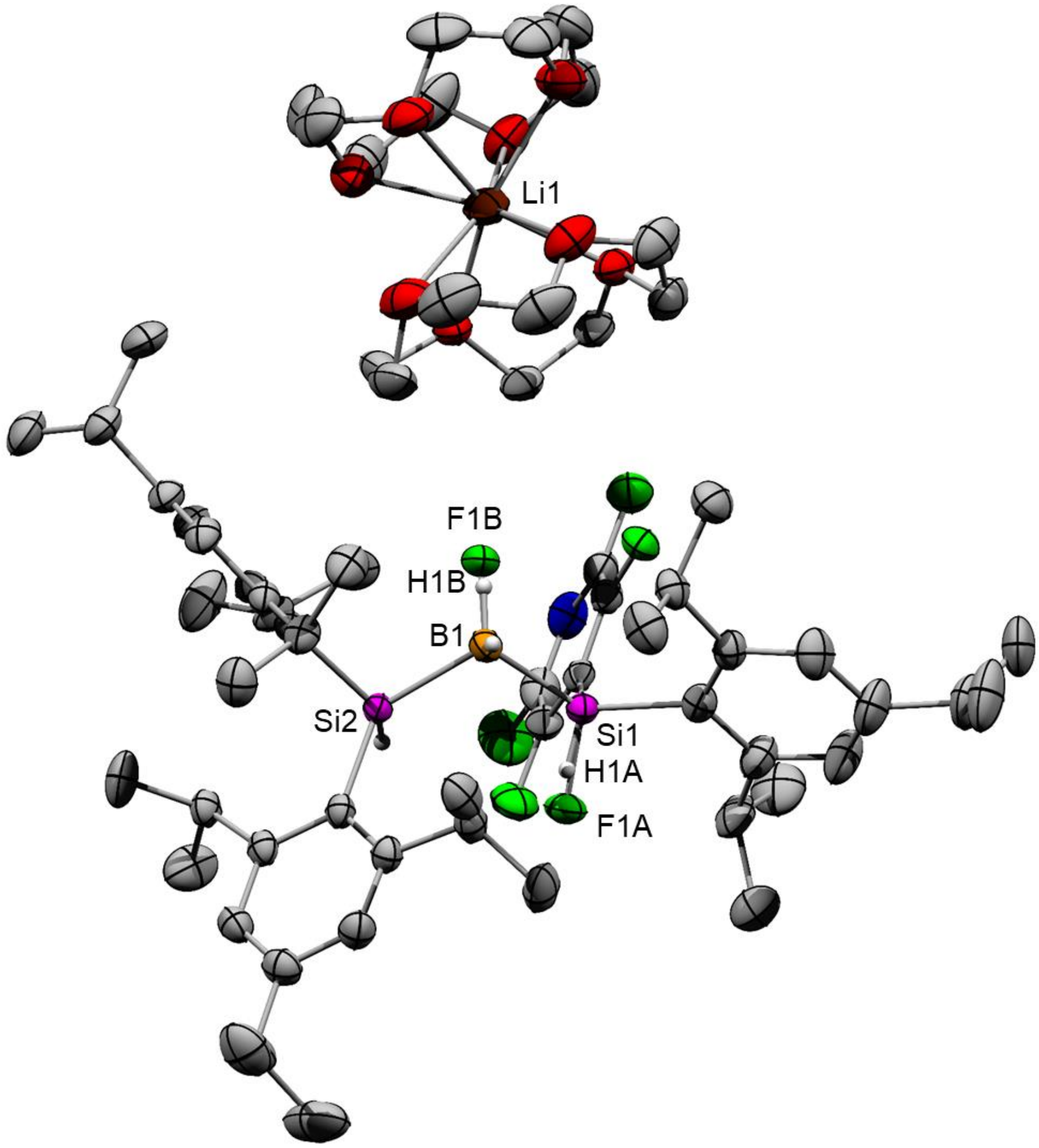

Refinement: All non H-atoms were located in the electron density maps and refined anisotropically. C-bound H atoms were placed in positions of optimized geometry and treated as riding atoms. Their isotropic displacement parameters were coupled to the corresponding carrier atoms by a factor of 1.2 (CH, CH2) or 1.5 (CH3). *Disorder:* A positional disorder was considered for F1a (bonded to Si1; fvar 2: 0.54) and F1b (bonded to B1; fvar 2: 0.46). One isopropyl-group (fvar 3: 0.60/0.40), one solvent toluene and n-pentane (fvar 4: 0.65/0.35 and fvar 6: 0.55/0.45) and one 12-crown-4 ether ligand of the Li(12-crown-4)$_2$ cation (fvar 0.69/0.31) is split over two positions.

Table S5. Crystal data and structure refinement for sh5735_b.

Identification code	sh5735_b
Empirical formula	$C_{151}H_{236}B_2F_{10}Li_2N_2O_{16}Si_4$
Formula weight	2673.26
Temperature	133(2) K

Wavelength	0.71073 Å	
Crystal system	Monoclinic	
Space group	*C*2/*c*	
Unit cell dimensions	a = 15.5037(7) Å	α = 90°.
	b = 22.7387(8) Å	β = 91.257(2)°.
	c = 43.976(2) Å	γ = 90°.
Volume	15499.4(11) $Å^3$	
Z	4	
Density (calculated)	1.146 Mg/m^3	
Absorption coefficient	0.109 mm^{-1}	
F(000)	5784	
Crystal size	0.360 x 0.320 x 0.140 mm^3	
Theta range for data collection	2.093 to 25.028°.	
Index ranges	−18<=h<=18, −27<=k<=25, −52<=l<=52	
Reflections collected	203210	
Independent reflections	13690 [R(int) = 0.0531]	
Completeness to theta = 25.028°	99.9 %	
Absorption correction	Semi-empirical from equivalents	
Max. and min. transmission	0.7455 and 0.7096	
Refinement method	Full-matrix least-squares on F^2	
Data / restraints / parameters	13690 / 1053 / 1147	
Goodness-of-fit on F^2	1.083	
Final R indices [I>2sigma(I)]	R1 = 0.0675, wR2 = 0.1797	
R indices (all data)	R1 = 0.0784, wR2 = 0.1887	
Extinction coefficient	n/a	
Largest diff. peak and hole	0.696 and -0.519 e.$Å^{-3}$	

3. Computational Details

Computations were carried out with the Gaussian 16 program package.[S6] Structural optimizations and frequency analyses of **2**, **3**, **4**, **Int1**, **Int2**, **Int3**, **TS1** and **TS2** were performed at the B3LYP-D3BJ/def2SVP level of theory. [S7,S8,S9] Structural optimization and frequency analysis to calculate ring-strain energy were performed at the B3LYP/def2SVP level of theory.[S7,S8,S9] Pictures of Kohn-Sham orbitals were displayed with ChemCraft.[S10] NBO calculations were run with the NBO 3.1 program[S11] package at the B3LYP-D3BJ/def2SVP level of theory. $[Li(dme)_2]^+$ was omitted in DFT and NBO calculations. The Multiwfn 3.8 was used to analyze the bonding properties of the compound 2.[S12] The ELF isosurface was rendered using the ChimeraX software.[S13]

3.1. Optimization of 2

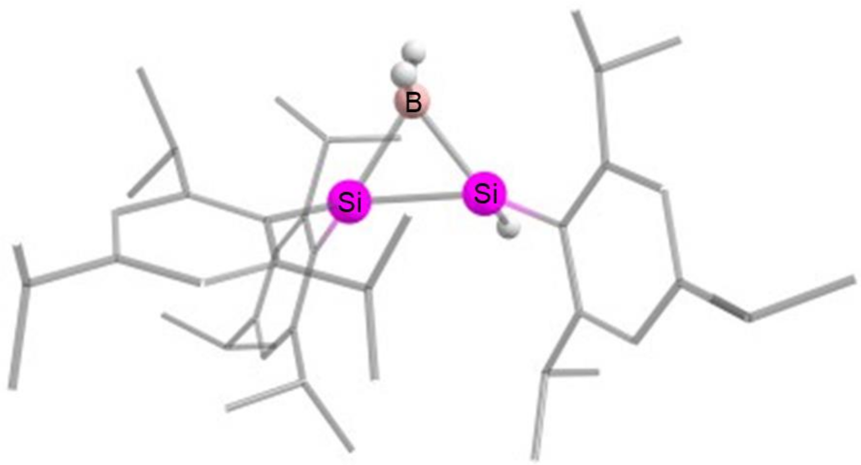

Figure S35. Optimized structure of **2**

Table S6. Atomic coordinates of the optimized structure **2**

NImag = 0

-2360.957492 Hartree

14	0.661797000	-0.397573000	0.882208000
14	-1.131308000	-1.874183000	0.827041000
1	-0.626560000	-3.146187000	0.210693000
5	-0.289433000	-1.157207000	2.514902000
1	0.162604000	-1.840516000	3.416128000
1	-0.973390000	-0.197544000	2.859299000
6	2.260809000	-1.059289000	0.026712000
6	3.211287000	-1.864835000	0.715631000
6	4.413473000	-2.225656000	0.096377000

1	5.126967000	-2.841472000	0.648855000
6	4.737807000	-1.806585000	-1.197337000
6	3.796262000	-1.036772000	-1.879972000
1	4.028206000	-0.709729000	-2.898177000
6	2.569853000	-0.673391000	-1.304038000
6	6.069638000	-2.164476000	-1.835053000
1	6.065381000	-1.734635000	-2.852344000
6	6.250546000	-3.683113000	-1.976048000
1	6.271324000	-4.172273000	-0.988634000
1	7.196424000	-3.925470000	-2.490056000
1	5.421521000	-4.127659000	-2.548200000
6	7.245339000	-1.534600000	-1.072246000
1	7.126607000	-0.442501000	-0.999012000
1	8.206123000	-1.747753000	-1.571573000
1	7.304642000	-1.929925000	-0.045098000
6	2.942301000	-2.375508000	2.125117000
1	2.175030000	-1.723204000	2.563153000
6	2.329400000	-3.782781000	2.076996000
1	2.076922000	-4.134237000	3.090989000
1	3.030672000	-4.504133000	1.621642000
1	1.403791000	-3.782593000	1.483477000
6	4.165423000	-2.331584000	3.048411000
1	4.628705000	-1.332091000	3.052073000
1	4.941969000	-3.060364000	2.759881000
1	3.865619000	-2.570655000	4.081960000
6	1.606215000	0.134957000	-2.166251000
1	0.665894000	0.225324000	-1.606393000
6	2.125763000	1.552535000	-2.444702000
1	1.376952000	2.139321000	-3.001811000
1	3.049897000	1.523916000	-3.047161000
1	2.341010000	2.087574000	-1.510187000
6	1.271121000	-0.595478000	-3.473858000
1	0.509700000	-0.037852000	-4.041863000
1	0.878884000	-1.603878000	-3.272039000

1	2.156987000	-0.702124000	-4.121253000
6	0.746843000	1.513482000	0.682926000
6	1.886599000	2.173932000	1.211795000
6	2.069469000	3.548025000	0.999409000
1	2.965117000	4.037081000	1.394954000
6	1.143746000	4.316791000	0.294265000
6	-0.005170000	3.674389000	-0.177668000
1	-0.756856000	4.260001000	-0.711307000
6	-0.220247000	2.303189000	0.004368000
6	1.381496000	5.796446000	0.043582000
1	2.336050000	6.056668000	0.534223000
6	1.536701000	6.093054000	-1.456011000
1	0.608965000	5.855547000	-2.001664000
1	2.342626000	5.485391000	-1.895716000
1	1.767865000	7.157798000	-1.631299000
6	0.286176000	6.671182000	0.671223000
1	0.503127000	7.743682000	0.528895000
1	0.196155000	6.476274000	1.751105000
1	-0.695397000	6.465056000	0.214381000
6	2.944800000	1.441834000	2.031345000
1	2.612616000	0.402063000	2.143827000
6	4.309153000	1.417291000	1.329412000
1	4.719616000	2.435112000	1.215408000
1	4.229682000	0.963754000	0.331406000
1	5.035369000	0.824758000	1.909738000
6	3.045999000	2.007934000	3.454754000
1	3.395196000	3.054189000	3.457020000
1	3.757269000	1.415955000	4.055057000
1	2.066115000	1.974812000	3.955110000
6	-1.530253000	1.702494000	-0.483238000
1	-1.358364000	0.623645000	-0.611151000
6	-2.608919000	1.854671000	0.599264000
1	-2.794749000	2.921577000	0.813185000
1	-2.286687000	1.371011000	1.532208000

1	-3.553162000	1.387361000	0.282637000
6	-2.023484000	2.256105000	-1.823331000
1	-2.912621000	1.694704000	-2.149562000
1	-1.252153000	2.169232000	-2.605349000
1	-2.317178000	3.316984000	-1.756142000
6	-2.914885000	-1.549161000	0.194876000
6	-3.193121000	-1.427314000	-1.196616000
6	-4.457095000	-1.003942000	-1.624425000
1	-4.657173000	-0.886669000	-2.691604000
6	-5.488905000	-0.720745000	-0.723991000
6	-5.234561000	-0.909215000	0.635263000
1	-6.033609000	-0.724247000	1.357985000
6	-3.979338000	-1.318599000	1.106735000
6	-3.829499000	-1.558135000	2.605670000
1	-2.760713000	-1.714653000	2.804004000
6	-4.568220000	-2.840925000	3.015782000
1	-5.654744000	-2.757441000	2.838928000
1	-4.414435000	-3.056104000	4.086959000
1	-4.201224000	-3.704400000	2.438803000
6	-4.269260000	-0.361541000	3.457264000
1	-5.349525000	-0.155068000	3.365015000
1	-3.723080000	0.547418000	3.165916000
1	-4.059287000	-0.553270000	4.522448000
6	-2.142431000	-1.814439000	-2.230936000
1	-1.163012000	-1.529306000	-1.808838000
6	-2.289339000	-1.120524000	-3.588398000
1	-3.212934000	-1.424578000	-4.108119000
1	-1.448666000	-1.393826000	-4.242811000
1	-2.299122000	-0.025953000	-3.488446000
6	-2.131773000	-3.340827000	-2.424945000
1	-1.991550000	-3.861673000	-1.468082000
1	-1.314761000	-3.643903000	-3.101513000
1	-3.085963000	-3.678342000	-2.864725000
6	-6.831505000	-0.206684000	-1.216134000

1	-6.795078000	-0.216141000	-2.319843000
6	-7.995940000	-1.109867000	-0.784983000
1	-8.952264000	-0.750079000	-1.201206000
1	-8.097429000	-1.130938000	0.312189000
1	-7.839118000	-2.145847000	-1.123615000
6	-7.063272000	1.246863000	-0.773960000
1	-8.009343000	1.643451000	-1.180797000
1	-6.240625000	1.896198000	-1.111032000
1	-7.110509000	1.318879000	0.324872000

3.2 Selected molecular orbitals of 2

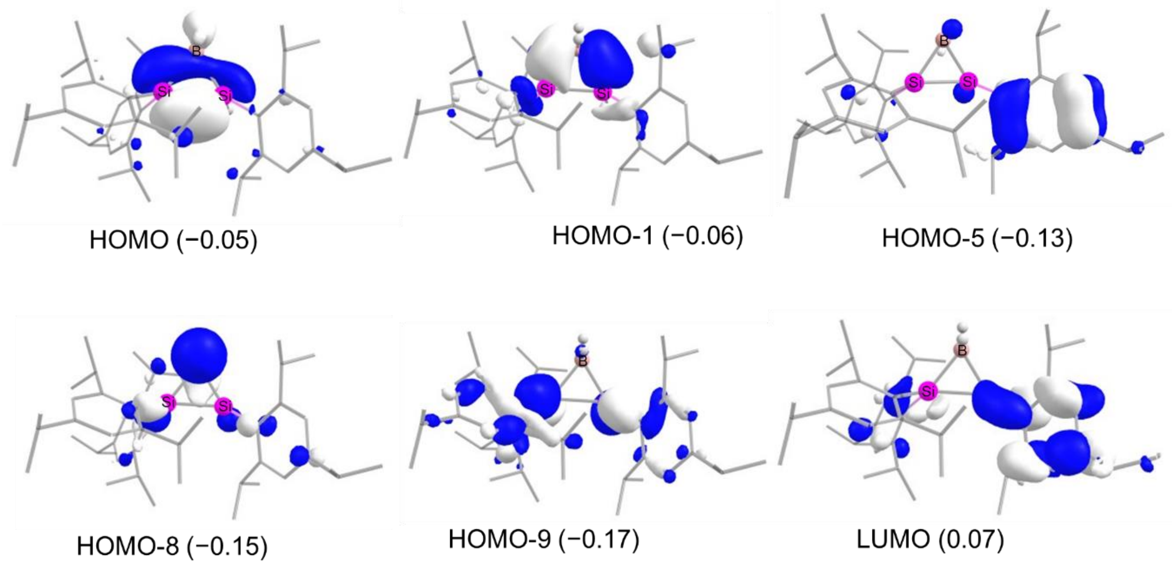

Figure S36. Selected molecular orbitals of **2** (energy in eV, contour value 0.04).

3.3 Natural bond orbital (NBO) analysis of 2

(Occupancy) Bond orbital/ Coefficients/ Hybrids

----

1. (1.87386) BD (1)Si 1 -Si 2

(51.43%) 0.7172*Si 1 s(18.58%)p 4.37(81.18%)d 0.01(0.24%)

-0.0000 0.0001 0.4304 -0.0251 -0.0002

-0.6906 0.0285 -0.0003 -0.3464 -0.0078

-0.0003 -0.4624 0.0151 0.0312 0.0294

0.0173 0.0141 -0.0083

(48.57%)   0.6969*Si   2 s(15.98%)p 5.23(83.49%)d 0.03(0.53%)

-0.0000 -0.0005 0.3982 -0.0345 0.0006

0.5096 -0.0040 -0.0000 0.6379 -0.0167

-0.0003 -0.4094 0.0198 0.0619 -0.0217

-0.0207 -0.0032 -0.0228

2. (1.88984) BD (1)Si   1 - B   4

(49.96%)   0.7068*Si   1 s(29.90%)p 2.34(69.91%)d 0.01(0.18%)

-0.0001 0.0004 0.5467 0.0103 0.0001

-0.0801 -0.0228 0.0003 -0.2322 0.0109

0.0004 0.7989 0.0049 0.0133 -0.0147

-0.0255 -0.0011 0.0279

(50.04%)   0.7074* B   4 s(21.55%)p 3.64(78.36%)d 0.00(0.10%)

-0.0002 0.4640 -0.0141 0.6369 0.0112

0.3409 0.0086 -0.5109 -0.0230 0.0080

-0.0234 -0.0123 0.0010 0.0137

6. (1.92794) BD (1)Si   2 - B   4

(50.50%)   0.7106*Si   2 s(33.05%)p 2.02(66.64%)d 0.01(0.31%)

-0.0001 0.0005 0.5749 0.0007 -0.0003

0.1196 0.0141 -0.0003 0.0623 0.0015

0.0003 0.8049 -0.0142 0.0329 0.0281

0.0204 0.0029 0.0291

(49.50%)   0.7036* B   4 s(21.07%)p 3.74(78.89%)d 0.00(0.05%)

0.0003 0.4584 -0.0235 -0.5076 -0.0215

-0.4875 0.0189 -0.5407 -0.0200 0.0050

0.0107 0.0112 0.0028 0.0137

Wiberg bond index matrix in the NAO basis:

Atom	1	2	3	4	5	6	7	8	9
----	------	------	------	------	------	------	------	------	------
1. Si	0.0000	0.9099	0.0048	0.9544	0.0101	0.0163	0.7554	0.0147	0.0107
2. Si	0.9099	0.0000	0.9206	1.0003	0.0100	0.0085	0.0126	0.0103	0.0006
3. H	0.0048	0.9206	0.0000	0.0072	0.0010	0.0119	0.0005	0.0000	0.0000
4. B	0.9544	1.0003	0.0072	0.0000	0.9627	0.9405	0.0140	0.0019	0.0003

3.4 Topological Study of 2

Bader's Quantum Theory of Atoms in Molecules (QTAIM) analysis was performed to examine the bonding nature of the BSi_2 subunit in compound **2**. The high electron density values (ρ(r) = 0.101 and 0.103 for B–Si, and 0.081 for Si–Si), along with the negative values of the Laplacian and total energy density, confirm the covalent nature of the bonding (Table S7). This further indicates that the B–Si bonds are stronger than the Si–Si bond in **2**. Additionally, the slightly lower electron density value for the Si-Si bond in **2** compared to literature reflects relatively lower bond strength (0.09 for H_3Si-SiH_3).[S14] To gain further insight, we performed Electron Localization Function (ELF) and Laplacian of electron density plot analyses. These analyses suggest that the electron densities are depleted along the internuclear axes and are not in a straight line for the B–Si and Si–Si bonds in **2**. Instead, the electron concentration in off-axis regions points to a bent-type σ bonding formulation for the related bonds (Figure S42). This was additionally affirmed by the bonding orbitals of the B–Si and Si–Si bonds, obtained from Natural Bond Orbital (NBO) analyses (Figure S43).

Table S7. QTAIM analysis of **2** carried out at B3LYP-D3(BJ)/def2-SVP level of theory. The topological parameters listed are in atomic units. ρ(r) - electron density, **∇**2ρ(r) - Laplacian of electron density, G(r) - Lagrangian kinetic energy, K(r) - Hamiltonian kinetic energy, V(r) - Potential energy density, H(r) - energy density, ELF - electron localization function.

	***ρ*(r):**	**∇^2*ρ*(r):**	**H(r)**	**G(r)**:	**K(r):**	**V(r):**	**ELF**
**B-Si1**	0.101	-0.034	-0.069	0.060	0.069	-0.130	0.96742
**B-Si2**	0.103	-0.027	-0.071	0.064	0.0717	-0.135	0.96558
**Si1-Si2**	0.081	-0.072	-0.050	0.032	0.050	-0.083	0.96512

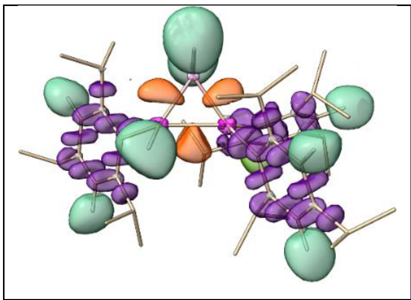	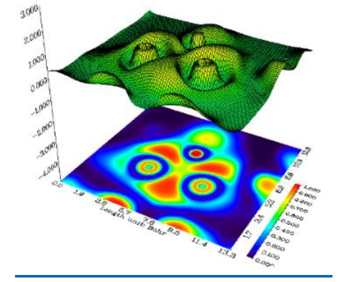	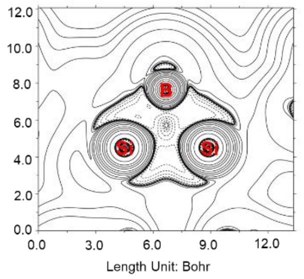
**ELF isosurface of 2 (Organe: B—Si and Si–Si bonds; Purple: C–C bonds; Green: H atoms)**	**3D ELF plot for BSi_2 subunit in 2**	**Laplacian of electron density for BSi_2 subunit in 2**

Figure S37. ELF and Laplacian contour plots for BSi_2 subunit in **2**. Hydrogen atoms except for Si–H and B–H are omitted for clarity.

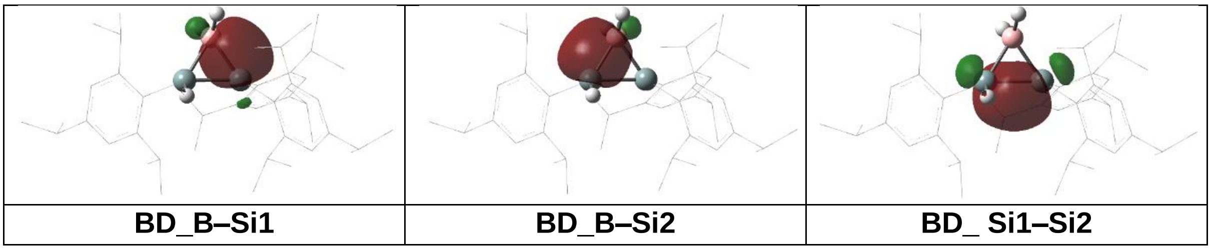

Figure S38. Bonding orbitals of B–Si and Si–Si bonds in **2**. Hydrogen atoms except for Si–H and B–H are omitted for clarity.

3.5 Ring Strain Energy Calculation

Table S8. Ring strain energies calculated for **2H**, C_3H_6, $BC_2H_6^-$, Si_3H_6 and BSi_2H_3 based on the designed isodesmotic equations. The relative energies are given in kcal mol^{-1}.

Compounds	Homodesmotic Equations	ΔH
**2H**	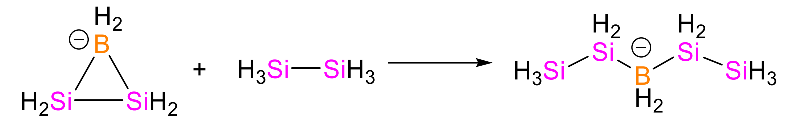	46.78
C_3H_6	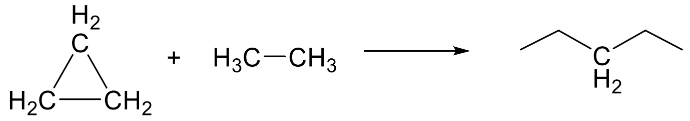	25.92
$BC_2H_6^-$	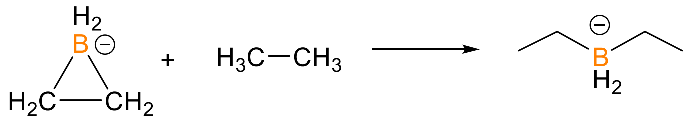	24.18
Si_3H_6	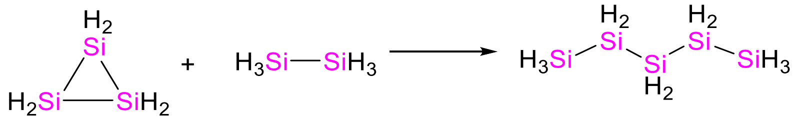	33.76
BSi_2H_3	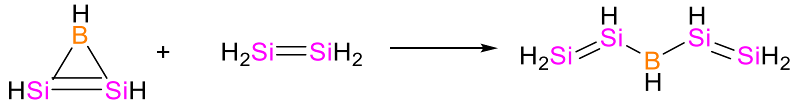	31.31

Atomic Coordinates

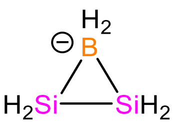

NImag = 0

-607.278789 Hartree

1 -1.879607000 -0.826988000 1.208856000

1 -1.879750000 -0.826899000 -1.208789000

1 0.000016000 2.055605000 -1.039002000

1	-0.000016000	2.055423000	1.039350000
1	1.879755000	-0.827097000	1.208650000
1	1.879603000	-0.826791000	-1.208995000
5	0.000000000	1.411327000	0.000117000
14	-1.161318000	-0.280710000	0.000012000
14	1.161318000	-0.280710000	-0.000059000

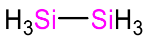

NImag = 0

-582.434630 Hartree

1	0.000000000	1.400748000	1.695934000
1	-1.213083000	-0.700374000	1.695934000
1	1.213083000	-0.700374000	1.695934000
1	0.000000000	-1.400748000	-1.695934000
1	1.213083000	0.700374000	-1.695934000
1	-1.213083000	0.700374000	-1.695934000
14	0.000000000	0.000000000	-1.178315000
14	0.000000000	0.000000000	1.178315000

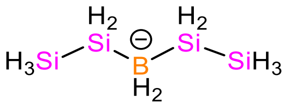

NImag = 0

-1189.790913 Hartree

1	1.194794000	1.456128000	3.841726000
1	-1.194794000	1.456128000	3.841726000
1	0.000000000	-0.283706000	4.952962000
1	-1.180546000	-1.602512000	1.872254000
1	1.180547000	-1.602511000	1.872253000
1	-1.016677000	1.153450000	0.000000000
1	1.016674000	1.153452000	0.000000000
1	-1.180546000	-1.602512000	-1.872254000
1	1.180547000	-1.602511000	-1.872253000
1	0.000000000	-0.283706000	-4.952962000

1	-1.194794000	1.456128000	-3.841726000
1	1.194794000	1.456128000	-3.841726000
5	-0.000001000	0.464332000	0.000000000
14	0.000000000	0.552865000	-3.697271000
14	0.000000000	-0.676994000	-1.666629000
14	0.000000000	-0.676994000	1.666629000
14	0.000000000	0.552865000	3.697271000

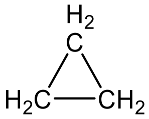

NImag = 0

-117.811159 Hartree

6	0.665714000	-0.560543000	0.000051000
6	0.152630000	0.856663000	0.000046000
6	-0.818366000	-0.296141000	-0.000026000
1	-1.379855000	-0.499131000	0.916828000
1	-1.379594000	-0.499604000	-0.916890000
1	0.257263000	1.444427000	-0.916811000
1	0.257579000	1.444445000	0.916870000
1	1.122346000	-0.945594000	0.916650000
1	1.122399000	-0.944413000	-0.917077000

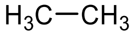

NImag = 0

-79.771636 Hartree

6	0.000000000	0.000000000	0.763894000
1	0.000000000	1.025500000	1.168858000
1	-0.888109000	-0.512750000	1.168858000
1	0.888109000	-0.512750000	1.168858000
6	0.000000000	0.000000000	-0.763894000
1	0.000000000	-1.025500000	-1.168858000
1	0.888109000	0.512750000	-1.168858000
1	-0.888109000	0.512750000	-1.168858000

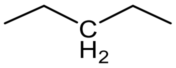

NImag = 0

-197.628323 Hartree

6	2.562109000	-0.321461000	0.000002000
1	2.613167000	-0.973401000	0.888416000
1	2.613171000	-0.973400000	-0.888412000
1	3.464525000	0.310821000	0.000004000
6	1.284673000	0.519517000	0.000000000
1	1.282929000	1.187560000	-0.880813000
1	1.282925000	1.187559000	0.880813000
6	0.000000000	-0.314608000	-0.000004000
1	0.000000000	-0.983648000	-0.881389000
1	0.000000000	-0.983655000	0.881375000
6	-1.284673000	0.519517000	0.000000000
1	-1.282928000	1.187561000	-0.880812000
1	-1.282925000	1.187558000	0.880814000
6	-2.562109000	-0.321461000	0.000002000
1	-3.464525000	0.310821000	0.000008000
1	-2.613173000	-0.973398000	-0.888414000
1	-2.613166000	-0.973404000	0.888414000

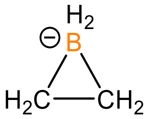

NImag = 0

-104.582055 Hartree

6	0.424192000	-0.765372000	0.000044000
6	0.424191000	0.765372000	0.000018000
1	0.812191000	1.256546000	-0.907789000
1	0.812183000	1.256629000	0.907785000
1	-1.666363000	-0.000042000	1.042203000
1	-1.666206000	0.000041000	-1.042465000
1	0.812345000	-1.256656000	-0.907638000

1	0.812026000	-1.256517000	0.907937000
5	-1.001295000	0.000000000	-0.000081000

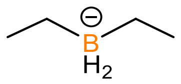

NImag = 0

-184.394581 Hartree

6	2.676755000	0.287654000	0.000000000
1	2.732724000	0.949965000	-0.883863000
1	2.732725000	0.949963000	0.883865000
1	3.597429000	-0.334897000	-0.000001000
6	1.368451000	-0.513203000	-0.000001000
1	1.375688000	-1.193215000	0.881160000
1	1.375688000	-1.193212000	-0.881163000
1	0.000000000	1.152688000	1.017517000
1	0.000000000	1.152693000	-1.017510000
6	-1.368451000	-0.513203000	-0.000001000
1	-1.375689000	-1.193215000	0.881159000
1	-1.375688000	-1.193212000	-0.881164000
6	-2.676755000	0.287654000	0.000000000
1	-3.597429000	-0.334897000	-0.000002000
1	-2.732725000	0.949962000	0.883865000
1	-2.732724000	0.949966000	-0.883863000
5	0.000000000	0.408799000	0.000002000

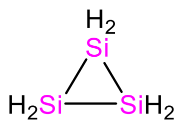

NImag = 0

-871.820615 Hartree

1	1.913146000	-1.045612000	-1.240588000
1	1.913563000	-1.045741000	1.240475000
1	-0.051355000	2.179950000	1.240244000
1	-0.050647000	2.179588000	-1.240729000
1	-1.862517000	-1.134261000	-1.240312000
1	-1.862148000	-1.133830000	1.240749000
14	-0.031726000	1.355849000	-0.000115000
14	-1.158534000	-0.705395000	0.000039000
14	1.190257000	-0.650461000	0.000088000

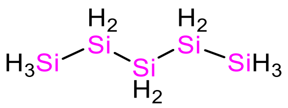

NImag = 0

-1454.312188 Hartree

1	3.942532000	1.387786000	-1.212595000
1	3.942536000	1.387785000	1.212590000
1	5.138967000	-0.341260000	-0.000005000
1	1.958166000	-1.685954000	1.208507000
1	1.958161000	-1.685953000	-1.208507000
1	0.000000000	1.394704000	1.206746000
1	0.000000000	1.394714000	-1.206728000
1	-1.958166000	-1.685955000	1.208505000
1	-1.958161000	-1.685951000	-1.208508000
1	-5.138967000	-0.341260000	-0.000010000
1	-3.942539000	1.387781000	1.212593000
1	-3.942530000	1.387790000	-1.212592000
14	-3.916206000	0.516710000	-0.000003000
14	-1.962711000	-0.804533000	0.000000000
14	0.000000000	0.510344000	0.000005000
14	1.962711000	-0.804533000	0.000000000
14	3.916206000	0.516710000	-0.000003000

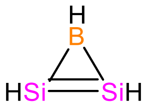

NImag = 0

-605.373499 Hartree

1	2.400911000	-0.939807000	-0.002637000
1	-0.001054000	2.542280000	0.000003000
1	-2.401360000	-0.938854000	0.003777000
5	-0.000149000	1.343290000	0.000094000
14	1.065816000	-0.263368000	0.000459000
14	-1.065656000	-0.263780000	-0.000574000

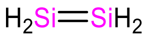

NImag = 0

-581.175485 Hartree

14	1.081214000	0.000002000	-0.067845000
1	1.832056000	1.236928000	0.280336000
1	1.831792000	-1.237110000	0.280280000
14	-1.081214000	0.000002000	0.067837000
1	-1.832015000	-1.236974000	-0.280261000
1	-1.831833000	1.237096000	-0.280248000

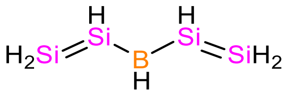

NImag = 0

-1186.601919 Hartree

14	2.934206000	-0.865090000	-0.000309000
1	4.416280000	-0.979576000	-0.000502000
1	2.255881000	-2.186868000	0.000481000
14	1.848511000	1.018363000	0.000209000
1	2.741174000	2.211386000	-0.000263000
5	-0.096396000	1.126074000	-0.000169000
1	-0.617968000	2.213909000	-0.000443000

14	-1.280486000	-0.412732000	0.000656000
1	-0.844396000	-1.836313000	0.000033000
14	-3.430203000	-0.106538000	-0.000512000
1	-4.454409000	-1.184601000	0.000705000
1	-4.022971000	1.255650000	0.000219000

3.6 Mechanism for the formation of 3 and IRC plot

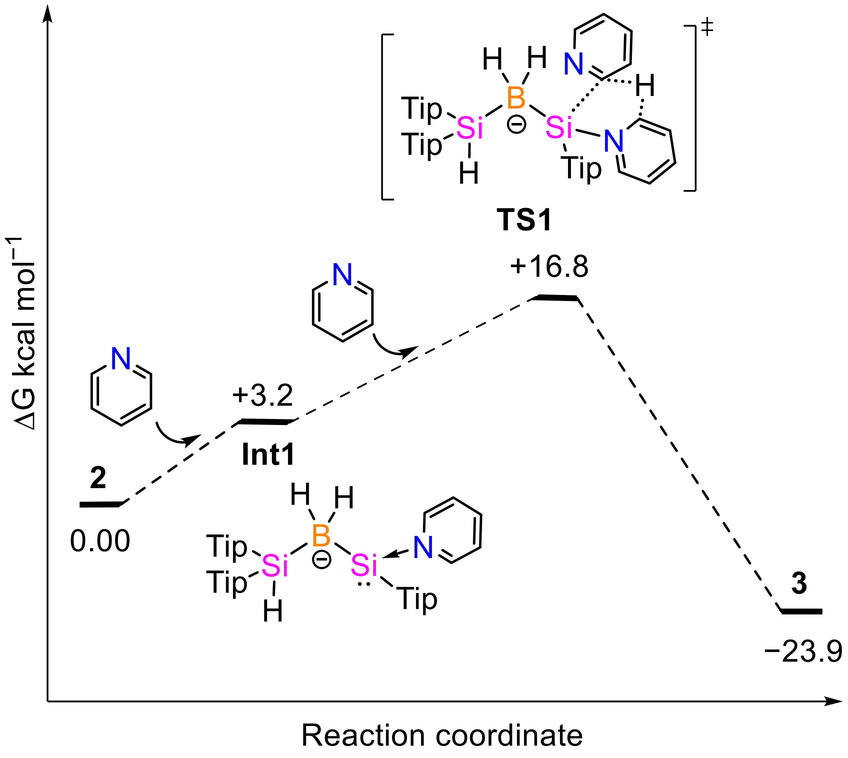

Figure S39. Computed energy profile for the formation of **3** from the reaction of **2** with two equivalent of pyridine.

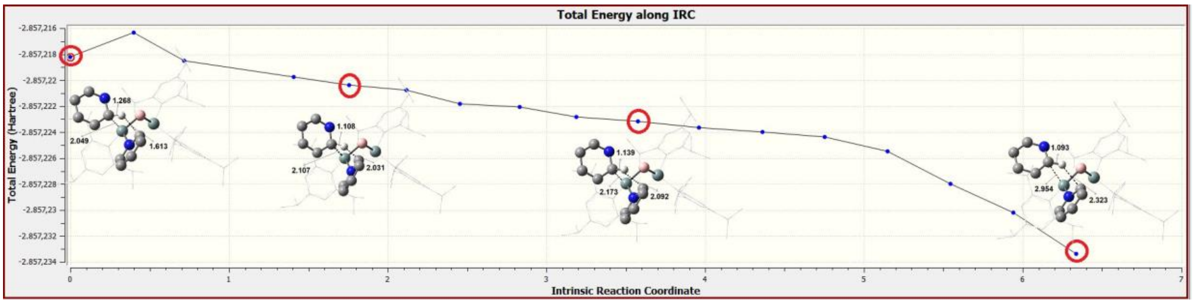

Figure S40. IRC plot of pyridine dissociation from TS1.

3.7 Optimization of 3

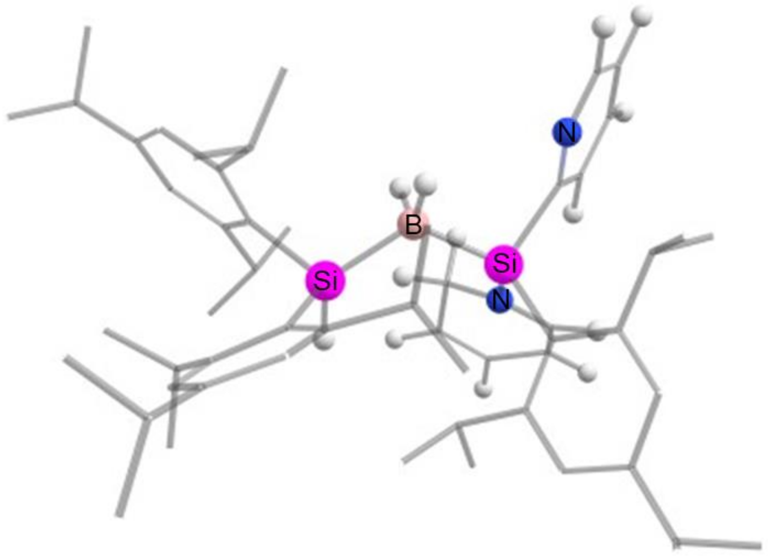

Figure S41. Optimized structure of **3**

Table S9. Atomic coordinates of the optimized structure **3**·[Li(dme)$_2$]

NImag = 0

-2857.287063 Hartree

14	-1.140006000	-0.274692000	0.354408000
1	-0.918867000	-0.416009000	1.832546000
14	2.104458000	-1.473602000	-0.244362000
7	2.571659000	-2.447184000	-2.858953000
7	2.347772000	-2.469555000	1.263042000
6	-1.518026000	1.627103000	0.193662000
6	-0.804126000	2.498098000	-0.670249000
6	-1.283817000	3.794116000	-0.915085000
6	-2.429848000	4.294388000	-0.299055000
6	-3.074109000	3.470409000	0.627746000
6	-2.638268000	2.165418000	0.887563000
6	-3.381483000	1.393480000	1.975708000
6	-3.164544000	2.047806000	3.349013000
6	-4.872697000	1.207285000	1.667341000
6	0.514133000	2.107852000	-1.333428000
6	0.382652000	1.937983000	-2.852945000

6	1.633013000	3.105776000	-0.995768000
6	-2.953868000	5.682665000	-0.624313000
6	-2.998739000	6.589790000	0.613827000
6	-4.326269000	5.611166000	-1.311741000
6	-2.864174000	-1.097409000	0.029253000
6	-3.570019000	-0.775467000	-1.159541000
6	-4.862777000	-1.268498000	-1.373812000
6	-5.492784000	-2.112844000	-0.454864000
6	-4.765974000	-2.491649000	0.674254000
6	-3.470117000	-2.014994000	0.923869000
6	-2.741588000	-2.568179000	2.145815000
6	-2.382353000	-4.047348000	1.935776000
6	-3.512714000	-2.353322000	3.455306000
6	-2.940580000	0.064126000	-2.265100000
6	-2.713870000	-0.778351000	-3.528386000
6	-3.723600000	1.348789000	-2.562527000
6	-6.913474000	-2.604598000	-0.674849000
6	-7.049888000	-3.417500000	-1.970600000
6	-7.918052000	-1.442440000	-0.640210000
6	3.255035000	0.053070000	0.092085000
6	3.110304000	0.749059000	1.326960000
6	3.970127000	1.813083000	1.638034000
6	4.963312000	2.253952000	0.767213000
6	5.063763000	1.610964000	-0.467873000
6	4.226146000	0.546660000	-0.823022000
6	4.397070000	-0.044283000	-2.215773000
6	5.529119000	-1.081481000	-2.237581000
6	4.606576000	1.009703000	-3.312168000
6	2.022568000	0.446520000	2.357887000
6	2.606365000	-0.040468000	3.691766000
6	1.108267000	1.658976000	2.589557000
6	5.851136000	3.433994000	1.122739000
6	7.343340000	3.079551000	1.057019000
6	5.528574000	4.651995000	0.242581000

6	2.781869000	-2.738831000	-1.556283000
6	3.284327000	-4.003957000	-1.193751000
6	3.583547000	-4.947023000	-2.178934000
6	3.366196000	-4.618818000	-3.516432000
6	2.854289000	-3.347330000	-3.795862000
6	3.587294000	-2.717314000	1.776947000
6	3.800167000	-3.421611000	2.931882000
6	2.640329000	-3.722613000	3.750161000
6	1.398619000	-3.580995000	3.239603000
6	1.246009000	-3.307773000	1.763531000
5	0.201237000	-1.251630000	-0.762025000
1	-0.216752000	-2.410763000	-0.764947000
1	0.222215000	-0.847563000	-1.914324000

3.8 Optimization of Int1

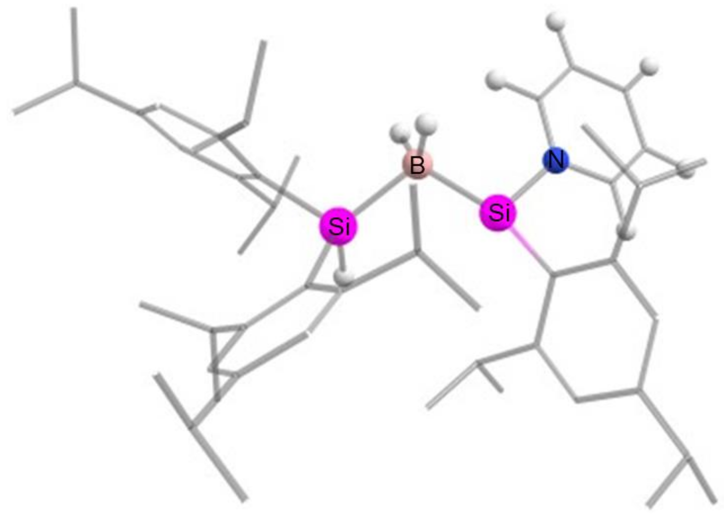

Figure S42. Optimized structure of **Int1**

Table S10. Atomic coordinates of the optimized structure **Int1**

NImag = 0

-2609.097592 Hartree

Si	0.65155200	-0.25242900	-0.44209000
Si	-2.41154900	0.05379000	-1.93038300
B	-0.40410400	-0.14380400	-2.15068800
H	0.02223900	0.66687300	-2.96273800
H	-0.23735500	-1.28245800	-2.58959200
C	1.31553800	1.25530300	0.60099500
C	1.13482600	2.61499600	0.23063000
C	1.81736800	3.63065600	0.91418500
H	1.68167200	4.66440300	0.58701100
C	2.65701800	3.36709900	1.99773400
C	2.76650300	2.04100600	2.41494000
H	3.38809600	1.81588700	3.28678700
C	2.11445300	0.99387700	1.74830900
C	3.42256000	4.47743300	2.69668300
H	3.97524200	4.01152400	3.53154500
C	2.48573300	5.53655400	3.29577300
H	1.90997300	6.04816200	2.50758400
H	3.05504900	6.30464500	3.84637400
H	1.76331400	5.07802100	3.98872000
C	4.45631300	5.11685600	1.75684000

H	5.14115700	4.35696500	1.34991300
H	5.05504400	5.88076300	2.28172600
H	3.96049200	5.60560600	0.90238000
C	0.18579300	3.05608600	-0.88108500
H	-0.39368600	2.17743300	-1.18726800
C	-0.81060700	4.11813400	-0.38686200
H	-1.61010800	4.26425600	-1.12800800
H	-0.32062000	5.09260900	-0.22383500
H	-1.27505200	3.81932000	0.56265000
C	0.93394500	3.56909000	-2.11996200
H	1.53699800	2.77678300	-2.58288500
H	1.60536100	4.40519400	-1.86017300
H	0.21951300	3.93137500	-2.87576900
C	2.27271800	-0.40152100	2.34650300
H	1.72831000	-1.10169400	1.70838100
C	3.73147400	-0.87524700	2.38279400
H	3.78952600	-1.91470300	2.74527200
H	4.34578500	-0.25123800	3.05357800
H	4.17744000	-0.84633200	1.37906600
C	1.61802600	-0.48069500	3.73447200
H	1.66630100	-1.51037100	4.12795900
H	0.55979500	-0.18225700	3.68683400
H	2.12173300	0.17888000	4.46056400
C	2.25453900	-1.30401100	-0.74494100
C	3.41064300	-0.68567000	-1.28282500
C	4.62248000	-1.38686700	-1.35883600
H	5.50962400	-0.88386000	-1.75591300
C	4.73541200	-2.71261200	-0.93794900
C	3.57230000	-3.34872000	-0.49465000
H	3.62118700	-4.40163500	-0.20301000
C	2.34233300	-2.68239100	-0.41569700
C	6.07234000	-3.43389000	-0.96453400
H	6.80798200	-2.73163200	-1.39477200
C	6.54354900	-3.78420600	0.45552200
H	5.84702600	-4.48994100	0.93704800
H	6.58983100	-2.88405700	1.08767600
H	7.54267400	-4.25251000	0.44174700

C	6.04295000	-4.67920100	-1.86259900
H	7.03673100	-5.15580700	-1.91383600
H	5.72972100	-4.42051100	-2.88592600
H	5.33275400	-5.42922600	-1.47811400
C	3.37096100	0.72934000	-1.84494300
H	2.35805500	1.11434700	-1.68925800
C	4.33157700	1.68976900	-1.13361800
H	5.38418800	1.39903500	-1.29054700
H	4.13675800	1.71046100	-0.05229700
H	4.20547600	2.71533400	-1.51718800
C	3.59232600	0.71932400	-3.36440300
H	4.59676500	0.34654800	-3.62626000
H	3.49333200	1.73750000	-3.77725400
H	2.84745800	0.07673800	-3.85752500
C	1.12371600	-3.50757500	-0.00691700
H	0.24488100	-2.85417500	-0.05510900
C	0.87860700	-4.65586700	-0.99710700
H	1.70819200	-5.38238700	-0.99346100
H	0.77209500	-4.26850900	-2.02155500
H	-0.04539700	-5.19863500	-0.74064400
C	1.21768000	-4.02612100	1.43506600
H	0.30880000	-4.58813000	1.70742800
H	1.32711500	-3.19699800	2.14956200
H	2.08151100	-4.69912300	1.56721000
C	-3.33570800	-0.59987300	-0.36000700
C	-3.46889000	0.09661800	0.87351900
C	-4.17972400	-0.48098800	1.93620400
H	-4.27342800	0.07106100	2.87625000
C	-4.75090900	-1.75017400	1.85254000
C	-4.59325300	-2.45180700	0.65313700
H	-5.01617500	-3.45511200	0.56628500
C	-3.91000700	-1.90605300	-0.43820600
C	-3.73866600	-2.75727300	-1.69066500
H	-3.60016300	-2.04319100	-2.52711700
C	-4.94749800	-3.63579100	-2.02886700
H	-5.08026700	-4.45977800	-1.30737100
H	-4.81507700	-4.09717900	-3.02128200

H	-5.87982300	-3.04930300	-2.04531300
C	-2.45295300	-3.59162600	-1.59915200
H	-2.49943700	-4.28879700	-0.74514600
H	-1.57496700	-2.94580900	-1.46405600
H	-2.29430500	-4.18188700	-2.51725600
C	-2.80308300	1.44211900	1.14289800
H	-2.32657700	1.76707000	0.20797600
C	-1.69762400	1.28931900	2.20043900
H	-2.12813700	1.04265500	3.18543700
H	-1.11716500	2.21911500	2.30543100
H	-0.99838900	0.49112700	1.92463300
C	-3.79567800	2.53164500	1.57790000
H	-4.61413900	2.65433700	0.85608900
H	-3.28285600	3.50283800	1.66783300
H	-4.23569100	2.30013600	2.56249200
C	-5.49881700	-2.35266700	3.02988000
H	-5.48824300	-1.59920700	3.83703800
C	-6.96792700	-2.64195600	2.68696500
H	-7.51345400	-3.02728900	3.56526200
H	-7.04599900	-3.39609800	1.88688400
H	-7.47773100	-1.73162600	2.33507700
C	-4.79658400	-3.61066900	3.56353800
H	-5.31347900	-4.00828200	4.45363500
H	-3.75275100	-3.39291400	3.83714100
H	-4.78012300	-4.40666700	2.80128900
H	-4.81114600	1.50504300	-1.09185700
N	-3.06401400	1.71799400	-2.24094200
C	-2.41120000	2.56904300	-3.13745400
C	-4.30087900	2.17321600	-1.78286900
C	-2.95766200	3.74965400	-3.58113300
H	-1.43660000	2.21710700	-3.47159600
C	-4.86653000	3.35594600	-2.20356500
H	-0.10946000	-1.02736400	0.59361500
C	-4.21666900	4.19595700	-3.12761500
H	-2.36980100	4.34599100	-4.28435600
H	-5.84166400	3.62606900	-1.78847500
H	-4.65424800	5.13606100	-3.46396000

3.9 Optimization of TS1

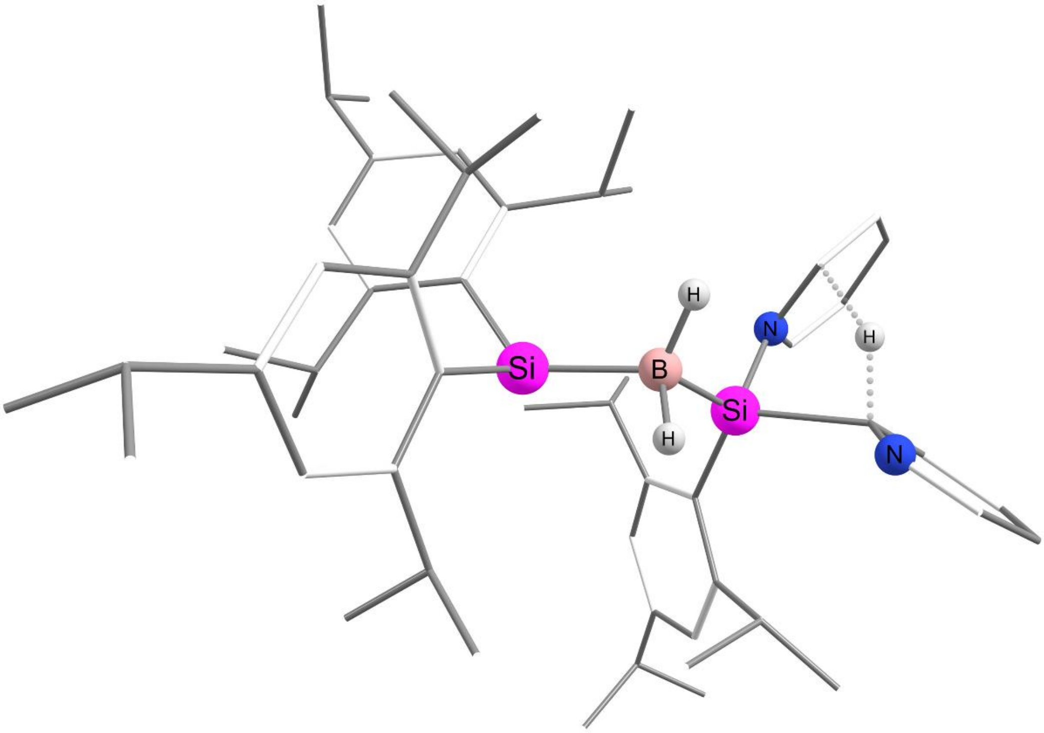

Figure S43. Optimized structure of **TS1**

Table S11. Atomic coordinates of the optimized structure **TS1**

NImag = 1

-2857.218197 Hartree

Si	1.02625400	-0.29469800	-0.10561600
Si	-2.22380000	0.20873200	-1.08349300
B	-0.37721500	-0.34548100	-1.52009400
H	-0.02792700	0.13334200	-2.59058400
H	-0.51855700	-1.56260300	-1.64208400
C	1.97142600	1.29518800	0.51330300
C	1.80380900	2.59103000	-0.04814100
C	2.65094600	3.64143300	0.33162200
H	2.51511100	4.61900900	-0.13745700
C	3.65699900	3.48241600	1.28567300
C	3.77577600	2.23018600	1.88742600
H	4.53772000	2.09105800	2.66013100
C	2.95994100	1.14872400	1.52612700
C	4.58914700	4.62394600	1.65391300
H	5.25916500	4.24923100	2.44769500
C	3.82706200	5.82988200	2.22242800

H	3.14470800	6.25806800	1.47037000
H	4.52220000	6.62711900	2.53595100
H	3.21944700	5.53834800	3.09315400
C	5.46755400	5.03302200	0.46151700
H	6.03200900	4.17085100	0.07414200
H	6.18607200	5.82033200	0.74657800
H	4.85131900	5.42263000	-0.36507300
C	0.70780600	2.93281800	-1.05613500
H	0.04901400	2.06143700	-1.13220900
C	-0.14582000	4.12089600	-0.58437500
H	-1.03198200	4.23561800	-1.22598200
H	0.41887200	5.06734500	-0.61820800
H	-0.49262300	3.97960100	0.44843400
C	1.26304800	3.21238100	-2.46078500
H	1.74759300	2.32530500	-2.88853600
H	2.00200000	4.03121400	-2.44276500
H	0.44946700	3.50956900	-3.14174400
C	3.17474700	-0.15311000	2.29415400
H	2.47683200	-0.89423500	1.89758100
C	4.58533700	-0.72737000	2.10675700
H	4.67300500	-1.70391200	2.61065200
H	5.35585900	-0.06072200	2.52937800
H	4.80726400	-0.88066500	1.04160900
C	2.82744800	0.01749900	3.78109500
H	2.90809600	-0.94694000	4.31076800
H	1.79928400	0.39008500	3.90434000
H	3.50584000	0.72964000	4.27965200
C	2.46771800	-1.51076100	-0.56088200
C	3.51965900	-1.07934600	-1.40589700
C	4.65162500	-1.88348900	-1.60118400
H	5.46291000	-1.52202700	-2.24047600
C	4.77891700	-3.13610300	-1.00008500
C	3.70016200	-3.59850400	-0.24095600
H	3.75193900	-4.59693200	0.20214200
C	2.54937100	-2.82676700	-0.03381600
C	6.04016200	-3.96550400	-1.17234400
H	6.71157100	-3.39493300	-1.83795300

C	6.76937400	-4.15591700	0.16670200
H	6.14705400	-4.72686500	0.87507900
H	6.99426900	-3.18472600	0.63393600
H	7.71671000	-4.70537100	0.03155200
C	5.75812500	-5.31544600	-1.84791500
H	6.69204500	-5.87824500	-2.01630300
H	5.25895000	-5.17420000	-2.81898200
H	5.09911900	-5.94118900	-1.22433100
C	3.44098400	0.23499700	-2.17001400
H	2.49987600	0.71858200	-1.88924500
C	4.57243000	1.20726900	-1.81458300
H	5.55565900	0.81018700	-2.11871800
H	4.59853600	1.40177400	-0.73325500
H	4.42717800	2.17260800	-2.32608800
C	3.35579000	-0.01634700	-3.68250600
H	4.26770000	-0.50461500	-4.06477500
H	3.23116600	0.93452600	-4.22764600
H	2.49557600	-0.66066900	-3.91841700
C	1.40027000	-3.47679000	0.73378100
H	0.56644400	-2.76549300	0.75140800
C	0.89507300	-4.73479700	0.01186300
H	1.67026700	-5.51758100	-0.03505800
H	0.59135500	-4.49475300	-1.01817800
H	0.02163600	-5.15725700	0.53445700
C	1.76300800	-3.77908900	2.19455200
H	0.90136600	-4.21341300	2.72842200
H	2.06367000	-2.86488200	2.72743200
H	2.59747300	-4.49699600	2.26357100
C	-2.96373900	-0.19697200	0.64865700
C	-2.84025500	0.65264500	1.78415700
C	-3.50160800	0.32226100	2.97688400
H	-3.41254800	0.99025300	3.83799100
C	-4.24836600	-0.84673200	3.11906400
C	-4.28431800	-1.72504700	2.03345900
H	-4.81017600	-2.67707900	2.13432800
C	-3.64883700	-1.43106500	0.82153200
C	-3.60553200	-2.52448700	-0.23881000

H	-3.29084700	-2.08544300	-1.19061500
C	-4.95893300	-3.19785800	-0.49290000
H	-5.28664200	-3.80522300	0.36782900
H	-4.88968800	-3.86564900	-1.36512700
H	-5.73484000	-2.44974400	-0.70902700
C	-2.52471600	-3.55301300	0.12842700
H	-2.76226800	-4.06301500	1.07766900
H	-1.54816100	-3.06230000	0.23834700
H	-2.42961000	-4.31550000	-0.66129000
C	-1.91338300	1.86873200	1.83104700
H	-1.55289400	2.06318600	0.81269200
C	-0.68291500	1.55614900	2.70031800
H	-0.97352300	1.43877300	3.75774600
H	0.05930600	2.36658200	2.63310600
H	-0.19537500	0.62945400	2.37618500
C	-2.58092000	3.15231300	2.35133800
H	-3.47932800	3.42716000	1.78196100
H	-1.87589500	3.99648600	2.28679000
H	-2.86939200	3.05178200	3.41009000
C	-4.95724000	-1.16995700	4.42353500
H	-4.77964800	-0.32007900	5.10575100
C	-6.47527100	-1.29850500	4.22844400
H	-6.98541700	-1.47841000	5.18992500
H	-6.71764100	-2.13862800	3.55754000
H	-6.89497100	-0.38481500	3.77973900
C	-4.37484100	-2.42603500	5.08929200
H	-4.85647800	-2.61907500	6.06287000
H	-3.29158900	-2.31906700	5.25379300
H	-4.52806500	-3.31557000	4.45695700
H	-3.96724800	2.30616800	0.16320800
N	-2.65373700	1.97120300	-1.41577000
C	-2.36042300	2.23837900	-2.77778000
C	-3.69561000	2.64370900	-0.83441300
C	-3.02664900	3.31935300	-3.42393400
H	-1.35432800	1.95670300	-3.09418800
C	-4.38273400	3.65264800	-1.45290100
H	0.48839500	-0.84915900	1.18001900

C	-4.03732400	4.00622800	-2.79808800
H	-2.72055200	3.57270200	-4.44203700
H	-5.18624600	4.16145600	-0.91857500
H	-4.56124700	4.81800100	-3.30828600
H	-2.90968700	0.78206600	-3.20226700
C	-3.42930200	-0.24936700	-2.67651200
C	-4.85505700	0.00327500	-2.51178200
N	-3.05007000	-1.39155700	-3.37433200
C	-5.78472500	-0.81481100	-3.11456400
H	-5.18289400	0.86181900	-1.92137800
C	-3.97479500	-2.14346700	-3.94928000
C	-5.35640200	-1.91801900	-3.88262300
H	-6.85382000	-0.61536300	-2.98327200
H	-3.60231100	-3.02053600	-4.50096900
H	-6.06224600	-2.58902500	-4.37631600

3.10 Mechanism for the formation of 4

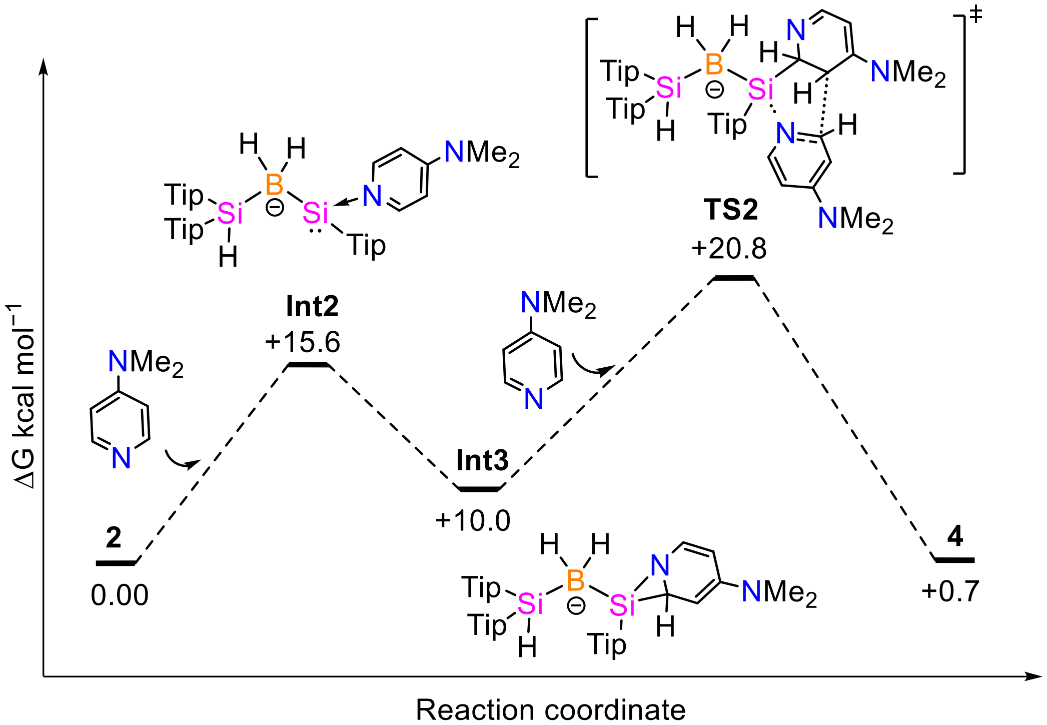

Figure S44. Computed energy profile for the formation of **4** from the reaction of **2** with two equivalent of DMAP.

3.11 Optimization of 4

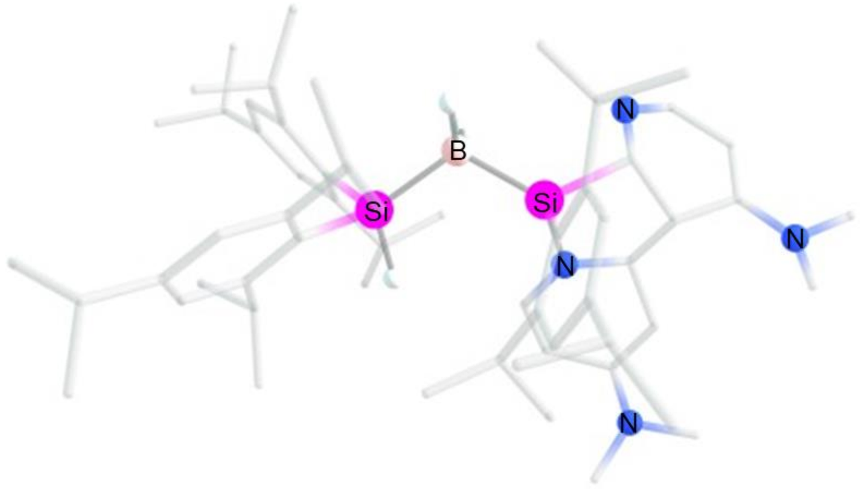

Figure S45. Optimized structure of **4**·[Li(dme)$_2$]

Table S12. Atomic coordinates of the optimized structure **4**·[Li(dme)$_2$]

NImag = 0

-3125.917888 Hartree

5	-0.127979000	-0.606530000	-1.590540000

1	-0.290814000	0.143447000	-2.546892000
1	-0.258281000	-1.773529000	-1.941606000
14	1.752775000	-0.400008000	-0.971993000
14	-1.545367000	-0.412029000	-0.157999000
1	-1.011418000	-0.754340000	1.199281000
7	2.303341000	-1.479071000	0.409152000
7	4.245751000	-2.941226000	3.845733000
7	2.922105000	-2.206868000	-2.845183000
7	6.742966000	-1.987635000	-0.909870000
6	1.606094000	-1.937280000	1.483080000
6	2.191907000	-2.590526000	2.531806000
6	3.646337000	-2.621542000	2.610556000
6	4.354438000	-2.307366000	1.488538000
6	3.595516000	-2.161754000	0.196011000
6	4.316081000	-1.372853000	-0.912605000
6	3.265655000	-1.070985000	-1.999037000
6	3.839131000	-3.088768000	-3.008118000
6	5.193814000	-3.034614000	-2.466245000
6	5.488657000	-2.140741000	-1.473985000
6	7.107578000	-0.755338000	-0.231189000
6	7.833521000	-2.745473000	-1.480092000
6	5.680058000	-3.081003000	3.863692000
6	3.726570000	-2.296424000	5.036788000
6	2.230074000	1.452548000	-0.618235000
6	2.622672000	2.298420000	-1.692212000
6	3.048850000	3.610809000	-1.438491000
6	3.070979000	4.151333000	-0.153483000
6	2.606377000	3.346881000	0.889184000
6	2.165919000	2.034538000	0.676478000
6	1.580995000	1.294195000	1.874465000
6	2.669878000	0.901830000	2.880478000
6	0.440798000	2.072339000	2.548202000
6	2.542437000	1.880173000	-3.162862000
6	3.908316000	1.947202000	-3.863405000

6	1.499045000	2.703997000	-3.929424000
6	3.499721000	5.589160000	0.084591000
6	4.631872000	5.695976000	1.115746000
6	2.300048000	6.463947000	0.482513000
6	-2.926027000	-1.737677000	-0.476056000
6	-2.985041000	-2.990617000	0.192464000
6	-4.063060000	-3.856575000	-0.037122000
6	-5.096423000	-3.543462000	-0.923114000
6	-5.006641000	-2.337003000	-1.619158000
6	-3.940881000	-1.449019000	-1.421149000
6	-3.905704000	-0.189636000	-2.277923000
6	-3.775623000	-0.534027000	-3.768365000
6	-5.097004000	0.737064000	-2.001263000
6	-1.864344000	-3.456172000	1.116765000
6	-2.322092000	-4.345270000	2.278359000
6	-0.756074000	-4.141417000	0.299144000
6	-6.274492000	-4.483962000	-1.114458000
6	-6.401598000	-4.962529000	-2.568134000
6	-7.583906000	-3.847344000	-0.623316000
6	-2.563892000	1.170747000	0.356242000
6	-3.515087000	1.031631000	1.411398000
6	-4.363387000	2.092361000	1.748791000
6	-4.310371000	3.324315000	1.092146000
6	-3.347940000	3.475561000	0.097928000
6	-2.476558000	2.438159000	-0.273309000
6	-1.456985000	2.769360000	-1.359635000
6	-2.140550000	3.080728000	-2.701098000
6	-0.548311000	3.932706000	-0.933564000
6	-3.659136000	-0.236619000	2.251930000
6	-5.051435000	-0.869605000	2.126840000
6	-3.278405000	0.025437000	3.716705000
6	-5.261263000	4.454560000	1.446735000
6	-6.718861000	4.078214000	1.140093000
6	-5.098592000	4.904416000	2.906129000

3.12 Optimization of Int2

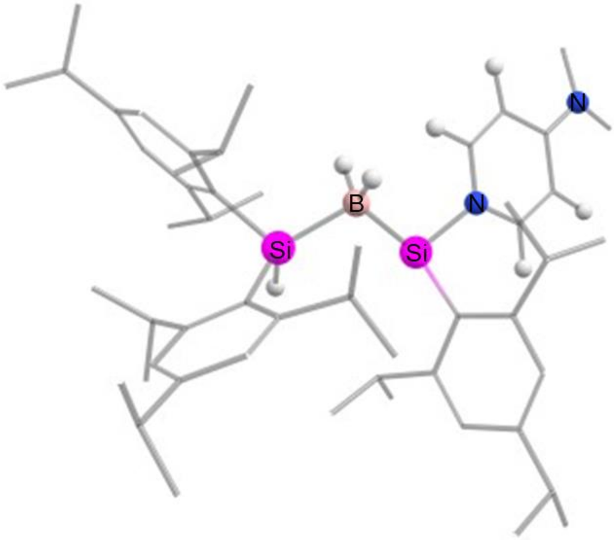

Figure S46. Optimized structure of **Int2**

Table S13. Atomic coordinates of the optimized structure **Int2**

NImag = 0

-2742.968636 Hartree

5	0.434281000	-1.230518000	-0.483119000
14	-1.165774000	-0.447901000	0.464422000
6	2.902825000	0.999688000	0.300809000
6	-1.979905000	1.282456000	0.073364000
6	-2.658357000	-1.679327000	0.321586000
6	-3.171893000	1.650807000	0.758659000
6	-3.882915000	2.799869000	0.385348000
6	-3.455220000	3.633426000	-0.647657000
6	-2.252197000	3.308648000	-1.275154000
6	-1.506221000	2.172931000	-0.928361000
6	-3.089725000	-2.528206000	1.378268000
6	-4.206859000	-3.355694000	1.198301000
6	-4.926860000	-3.388611000	0.001157000
6	-4.477286000	-2.583642000	-1.046326000
6	-3.358638000	-1.750788000	-0.907984000
6	2.596564000	1.912662000	1.365064000
6	3.182651000	3.176476000	1.438937000

6	4.091766000	3.624781000	0.474678000
6	4.346743000	2.770692000	-0.592768000
6	3.756880000	1.497889000	-0.730737000
6	1.612297000	1.548391000	2.476012000
6	4.712340000	5.008663000	0.546242000
6	2.335223000	1.348271000	3.813251000
6	0.451789000	2.543485000	2.594320000
6	3.649733000	6.107284000	0.383675000
6	5.524848000	5.211792000	1.833625000
6	4.081118000	0.857111000	-2.093308000
6	5.482759000	0.224780000	-2.147765000
6	3.031387000	-0.069070000	-2.705923000
6	-6.161169000	-4.263254000	-0.143791000
6	-3.727919000	0.861430000	1.941206000
6	-4.267581000	4.846707000	-1.067303000
6	-0.183603000	1.978037000	-1.665961000
6	-2.336453000	-2.584376000	2.705654000
6	-2.920555000	-0.949901000	-2.128495000
6	-6.010000000	-5.294410000	-1.271768000
6	-7.428900000	-3.415988000	-0.333939000
6	-4.784126000	4.702689000	-2.507117000
6	-3.483429000	6.154862000	-0.887136000
1	0.255128000	-2.432165000	-0.260160000
1	0.442507000	-1.033206000	-1.692964000
1	-0.915729000	-0.325144000	1.939671000
6	-1.060739000	-3.431036000	2.565994000
6	-3.175453000	-3.054788000	3.898264000
6	-2.513160000	-1.871841000	-3.285778000
6	-3.968877000	0.085027000	-2.557014000
6	0.746596000	3.187214000	-1.485170000
6	-0.401054000	1.684502000	-3.158162000
6	-3.690156000	1.695843000	3.230561000
6	-5.130761000	0.300650000	1.671373000
14	2.129608000	-0.777417000	0.559515000

7	3.516259000	-1.925194000	-0.060814000
6	3.232083000	-3.054832000	-0.783701000
6	4.787099000	-1.834385000	0.434187000
6	4.128971000	-4.101705000	-0.934830000
6	5.739278000	-2.812275000	0.280859000
6	5.436918000	-4.019960000	-0.415574000
6	5.932863000	-6.312426000	-1.080619000
6	7.592257000	-4.992753000	0.202704000
7	6.375744000	-5.034447000	-0.574620000

3.13 Optimization of Int3

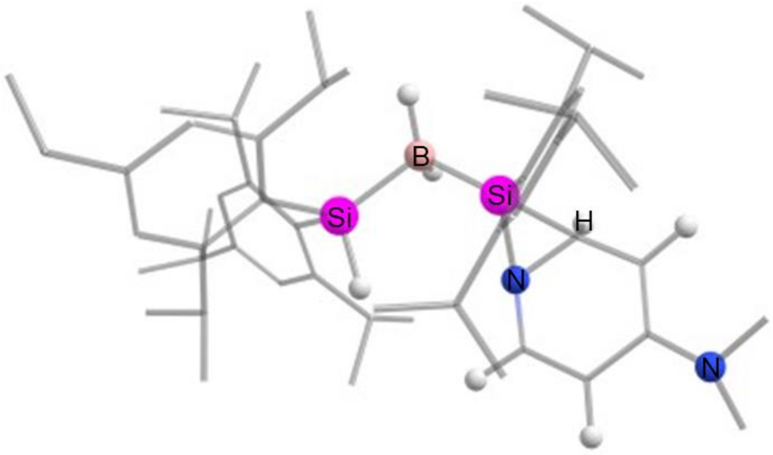

Figure S47. Optimized structure of **Int3**

Table S14. Atomic coordinates of the optimized structure **Int3**

NImag = 0

-2742.978070 Hartree

5	0.096993000	-0.973535000	1.813517000
14	1.199356000	-0.440691000	0.213321000
6	-3.020097000	0.031143000	0.764113000
6	1.559609000	1.387223000	-0.342050000
6	2.927430000	-1.276030000	0.378097000
6	2.436855000	1.612120000	-1.438866000
6	2.803923000	2.917606000	-1.795209000
6	2.330074000	4.035289000	-1.108374000
6	1.423921000	3.815380000	-0.068951000
6	1.017740000	2.528417000	0.308674000
6	3.165490000	-2.553954000	-0.185877000
6	4.465645000	-3.079083000	-0.194041000
6	5.541581000	-2.405344000	0.390054000
6	5.273812000	-1.201497000	1.049411000
6	3.990617000	-0.640103000	1.064515000
6	-3.156845000	0.604881000	-0.528792000
6	-4.049969000	1.661705000	-0.754097000
6	-4.832959000	2.198826000	0.264166000

6	-4.703606000	1.633540000	1.532818000
6	-3.823093000	0.576847000	1.812252000
6	-2.368514000	0.123734000	-1.742238000
6	-5.758444000	3.377945000	0.019772000
6	-3.309224000	-0.456915000	-2.810612000
6	-1.479929000	1.225308000	-2.336344000
6	-4.958420000	4.646167000	-0.317427000
6	-6.807095000	3.077703000	-1.060660000
6	-3.799926000	0.154859000	3.287860000
6	-4.148692000	-1.313383000	3.562024000
6	-2.505715000	0.575312000	3.999083000
6	6.949365000	-2.973853000	0.323589000
6	2.977868000	0.481707000	-2.312134000
6	2.781182000	5.436755000	-1.482981000
6	-0.023252000	2.426661000	1.420441000
6	2.030960000	-3.394956000	-0.770217000
6	3.757249000	0.611653000	1.903345000
6	7.518864000	-3.274240000	1.717904000
6	7.887047000	-2.052620000	-0.472073000
6	3.578645000	6.089633000	-0.343455000
6	1.605018000	6.323538000	-1.917376000
1	0.526141000	-2.097373000	2.062014000
1	0.205674000	-0.224699000	2.765123000
1	0.647204000	-0.995456000	-1.067891000
6	2.024909000	-4.827269000	-0.218798000
6	2.044600000	-3.390908000	-2.305348000
6	3.916309000	0.293533000	3.397618000
6	4.619484000	1.806467000	1.479684000
6	-1.260303000	3.293525000	1.143819000
6	0.584388000	2.752430000	2.791841000
6	2.365210000	0.538786000	-3.719243000

6	4.510582000	0.438526000	-2.365512000
14	-1.718770000	-1.354122000	1.110751000
7	-1.661539000	-2.723504000	-0.049384000
6	-2.588304000	-3.050428000	1.056498000
6	-1.932403000	-3.163531000	-1.311380000
6	-3.997215000	-3.220196000	0.595199000
1	-2.210258000	-3.862016000	1.710928000
6	-3.139623000	-3.688232000	-1.651423000
1	-1.133639000	-3.037968000	-2.053501000
6	-4.252184000	-3.546826000	-0.700401000
1	-4.807271000	-3.066477000	1.308458000
1	-3.320223000	-4.014590000	-2.674951000
6	-6.640120000	-3.129645000	-0.420983000
6	-5.901874000	-4.820605000	-1.970499000
7	-5.561681000	-3.614584000	-1.245669000

3.14 Optimization of TS2

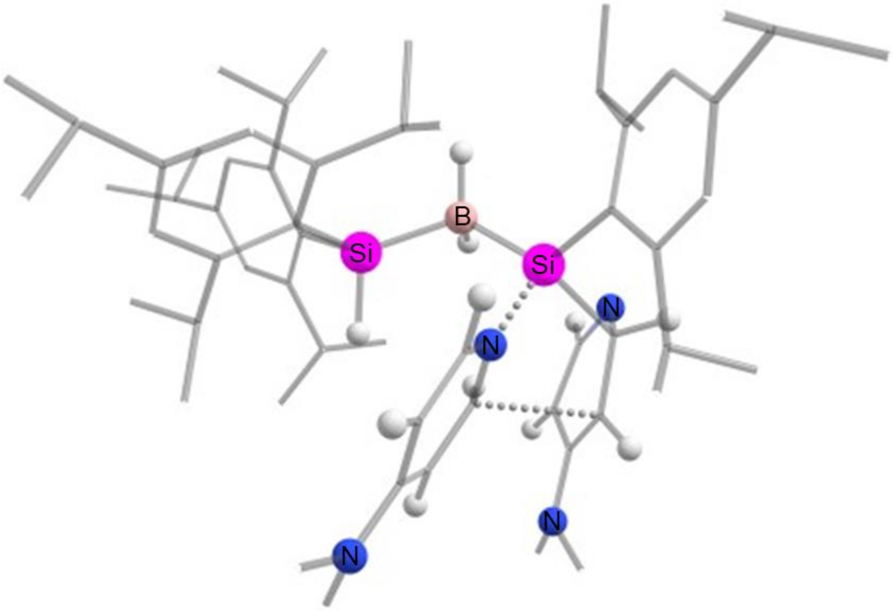

Figure S48. Optimized structure of **TS2**

Table S15. Atomic coordinates of the optimized structure **TS2**

NImag = 1

-3125.005037 Hartree

5	-0.080643000	0.282103000	-1.914725000
1	0.007581000	-0.678940000	-2.658577000
1	0.272952000	1.291187000	-2.512580000
14	-1.926140000	0.704720000	-1.194554000
14	1.281713000	0.117378000	-0.446242000
1	1.186945000	1.240224000	0.523581000
7	-1.554441000	1.074896000	0.631103000
7	-0.413812000	2.337226000	4.481580000
7	-2.299590000	2.950497000	-2.855201000
7	-1.815407000	5.530719000	0.525786000
6	-1.739106000	0.290892000	1.710251000
1	-2.171946000	-0.688338000	1.512717000
6	-1.394970000	0.662764000	2.986821000
1	-1.537412000	-0.055430000	3.789746000
6	-0.803388000	1.951034000	3.211719000

6	-0.601795000	2.761534000	2.092545000
1	-0.098826000	3.723049000	2.155363000
6	-1.001309000	2.342659000	0.811377000
1	-0.539769000	2.790279000	-0.066277000
6	-2.775325000	3.514659000	-0.449251000
1	-3.564502000	3.475871000	0.299271000
6	-2.830689000	2.521336000	-1.580842000
1	-3.865622000	2.173070000	-1.735036000
6	-1.480771000	3.953064000	-2.837689000
1	-1.014568000	4.215749000	-3.801810000
6	-1.163556000	4.787323000	-1.715855000
1	-0.465121000	5.608878000	-1.857180000
6	-1.939211000	4.640172000	-0.551039000
6	-2.785764000	5.468901000	1.593404000
6	-1.184589000	6.809988000	0.300327000
6	0.351938000	3.554410000	4.633154000
6	-0.396360000	1.367236000	5.556171000
6	-3.387066000	-0.570721000	-1.167149000
6	-3.471814000	-1.578410000	-2.177143000
6	-4.421555000	-2.602273000	-2.067067000
6	-5.334374000	-2.664412000	-1.011820000
6	-5.342057000	-1.604550000	-0.107013000
6	-4.422142000	-0.547886000	-0.187464000
6	-4.699931000	0.650722000	0.722805000
6	-5.880033000	1.462073000	0.157695000
6	-4.983615000	0.280530000	2.187018000
6	-2.596804000	-1.514616000	-3.425350000
6	-2.945900000	-0.275693000	-4.272368000
6	-2.633465000	-2.766095000	-4.306795000
6	-6.284584000	-3.840689000	-0.868932000
6	-7.757456000	-3.407409000	-0.860226000
6	-5.942721000	-4.674189000	0.376301000
6	3.078030000	0.301668000	-1.153785000
6	3.800906000	1.525164000	-1.096218000

6	5.135906000	1.568928000	-1.522438000
6	5.797811000	0.445291000	-2.022813000
6	5.068693000	-0.740756000	-2.128187000
6	3.730683000	-0.821035000	-1.720423000
6	3.000855000	-2.139052000	-1.941382000
6	2.884749000	-2.466150000	-3.436628000
6	3.612837000	-3.299238000	-1.146539000
6	3.145919000	2.825340000	-0.637004000
6	4.076101000	3.757428000	0.148435000
6	2.508327000	3.549472000	-1.833967000
6	7.259345000	0.516872000	-2.432653000
6	7.461650000	0.169493000	-3.914796000
6	8.136946000	-0.365319000	-1.531203000
6	1.438542000	-1.365987000	0.808679000
6	2.469761000	-1.292451000	1.790066000
6	2.753001000	-2.391581000	2.609520000
6	2.043886000	-3.591509000	2.509957000
6	0.989280000	-3.637851000	1.600483000
6	0.659504000	-2.551310000	0.774204000
6	-0.575653000	-2.711527000	-0.109177000
6	-0.332203000	-3.618879000	-1.323019000
6	-1.787593000	-3.230462000	0.685165000
6	3.282011000	-0.023065000	2.044535000
6	4.793523000	-0.240911000	1.904393000
6	2.903898000	0.597375000	3.398452000
6	2.402658000	-4.798192000	3.360134000
6	3.801757000	-5.329572000	3.012458000
6	2.277765000	-4.503709000	4.862188000

4. References:

[S1] D. Scheschkewitz, *Angew. Chem.* **2004**, *116*, 3025; *Angew. Chem. Int. Ed.* **2004**, *43*, 2965.

[S2] G. R. Fulmer, A. J. M. Miller, N. H. Sherden, H. E. Gottlieb, A. Nudelman, B. M. Stoltz, J. E. Bercaw, K. I. Goldberg, *Organometallics* **2010**, *29*, 2176

[S3] Sheldrick, G. M. (2015). Acta Cryst. A71, 3-8.

[S4] Sheldrick, G. M. (2015). Acta Cryst. C71, 3-8.

[S5] C. B. Hübschle, G. M. Sheldrick, B.Dittrich, *J. Appl. Crystallogr.* **2011**, *44*, 1281-1284.

[S6] M. J. Frisch, G. W. Trucks, H. B. Schlegel, G. E. Scuseria, M. A. Robb, J. R. Cheeseman, G. Scalmani, V. Barone, G. A. Petersson, H. Li, X. Nakatsuji, M. Caricato, A. V. Marenich, J. Bloino, B. G. Janesko, R. Gomperts, B. Mennucci, H. P. Hratchian, J. V. Ortiz, A. F. Izmaylov, J. L. Sonnenberg, D. Williams-Young, F. Ding, F. Lipparini, F. Egidi, J. Goings, B. Peng, A. Petrone, T. Henderson, D. Ranasinghe, V. G. Zakrzewski, J. Gao, N. Rega, G. Zheng, W. Liang, M. Hada, M. Ehara, K. Toyota, R. Fukuda, J. Hasegawa, M. Ishida, T. Nakajima, Y. Honda, O. Kitao, H. Nakai, T. Vreven, K. Throssell, J. A. Montgomery, Jr., J. E. Peralta, F. Ogliaro, M. J. Bearpark, J. J. Heyd, E. N. Brothers, K. N. Kudin, V. N. Staroverov, T. A. Keith, R. Kobayashi, J. Normand, K. Raghavachari, A. P. Rendell, J. C. Burant, S. S. Iyengar, J. Tomasi, M. Cossi, J. M. Millam, M. Klene, C. Adamo, R. Cammi, J. W. Ochterski, R. L. Martin, K. Morokuma, O. Farkas, J. B. Foresman, D. J. Fox, Gaussian 16, Revision C.01, Gaussian, Inc., Wallingford CT, **2019**.

[S7] a) J. P. Perdew, *Phys. Rev. B* **1986**, *33*, 8822 – 8824; b) A. D. Becke, *Phys. Rev. A* **1988**, *38*, 3098.

[S8] a) A. Schäfer, H. Horn, R. Ahlrichs, *J. Chem. Phys.* **1992**, *97*, 2571; b) A. Schäfer, C. Huber, R. Ahlrichs, *J. Chem. Phys.* **1994**, *100*, 5829; c) F. Weigend, R. Ahlrichs, *Phys. Chem. Chem. Phys.* **2005**, *7*, 3297; d) F. Weigend, *Phys. Chem. Chem. Phys.* **2006**, *8*, 1057.

[S9] S. Grimme, J. Antony, S. Ehrlich, H. J. Krieg, Chem. Phys. **2010**, *132*, 154104.

[S10] Chemcraft - graphical software for visualization of quantum chemistry computations. https://www.chemcraftprog.com

[S11] E. D. Glendening, J, K. Badenhoop, A. E. Reed, J. E. Carpenter, F. Weinhold, NBO Version 3.1, Theoretical Chemistry Institute, University of Wisconsin, Madison, **2010**.

[S12] T. Lu, F. Chen, *J. Comp. Chem.* **2010**, 33, 580-590.

[S13] E. C. Meng, T. D. Goddard, E. F. Pettersen, G. S. Couch, Z. J. Pearson, J. H. Morris, T. E. Ferrin, *Protein Science* **2023**, *32,* e4792.

[S14] a) C. L. Firme,O.A.C. Antunes, P. M. Esteves, *Chem. Phys. Letter* **2009**, *468,* 129-133; b) O. Sichevych, L. Akselrud, B. Böhme, M. Bobnar, M. Baitinger, F. R. Wagner, Y. Grin, *Z. Anorg. Allg. Chem.* **2023**, *649*, e202300116; c) M. Nazish, C. M. Legendre, N. Graw, R. Herbst-Irmer, S. Muhammed, P. Parameswaran, D. Stalke, H. W. Roesky, *Inorg. Chem.* **2023,** *62*, *24*, 9306–9313.
